# Naturally Occurring Isocoumarins Derivatives from Endophytic Fungi: Sources, Isolation, Structural Characterization, Biosynthesis, and Biological Activities

**DOI:** 10.3390/molecules25020395

**Published:** 2020-01-17

**Authors:** Ahmad Omar Noor, Diena Mohammedallam Almasri, Alaa Abdullah Bagalagel, Hossam Mohamed Abdallah, Shaimaa Gamal Abdallah Mohamed, Gamal Abdallah Mohamed, Sabrin Ragab Mohamed Ibrahim

**Affiliations:** 1 Pharmacy Practice Department, Faculty of Pharmacy, King Abdulaziz University, Jeddah 21589, Saudi Arabia; Aonoor@kau.edu.sa (A.O.N.); dalmasri@kau.edu.sa (D.M.A.); abagalagel@kau.edu.sa (A.A.B.); 2 Department of Natural Products and Alternative Medicine, Faculty of Pharmacy, King Abdulaziz University, Jeddah 21589, Saudi Arabia; hmafifi2013@gmail.com (H.M.A.); gamals2001@yahoo.com (G.A.M.); 3 Department of Pharmacognosy, Faculty of Pharmacy, Cairo University, Cairo 11562, Egypt; 4 Faculty of Dentistry, British University, El Sherouk City, Suez Desert Road, Cairo 11837, Egypt; shaimaag1973@gmail.com; 5 Pharmacognosy Department, Faculty of Pharmacy, Al-Azhar University, Assiut Branch, Assiut 71524, Egypt; 6 Department of Pharmacognosy and Pharmaceutical Chemistry, College of Pharmacy, Taibah University, Al Madinah Al-Munawwarah 30078, Saudi Arabia; 7 Department of Pharmacognosy, Faculty of Pharmacy, Assiut University, Assiut 71526, Egypt

**Keywords:** endophytes, isocoumarins, dihydroisocoumarins, biosynthesis, biological activities

## Abstract

Recently, the metabolites separated from endophytes have attracted significant attention, as many of them have a unique structure and appealing pharmacological and biological potentials. Isocoumarins represent one of the most interesting classes of metabolites, which are coumarins isomers with a reversed lactone moiety. They are produced by plants, microbes, marine organisms, bacteria, insects, liverworts, and fungi and possessed a wide array of bioactivities. This review gives an overview of isocoumarins derivatives from endophytic fungi and their source, isolation, structural characterization, biosynthesis, and bioactivities, concentrating on the period from 2000 to 2019. Overall, 307 metabolites and more than 120 references are conferred. This is the first review on these multi-facetted metabolites from endophytic fungi.

## 1. Introduction

**Figure 1:**
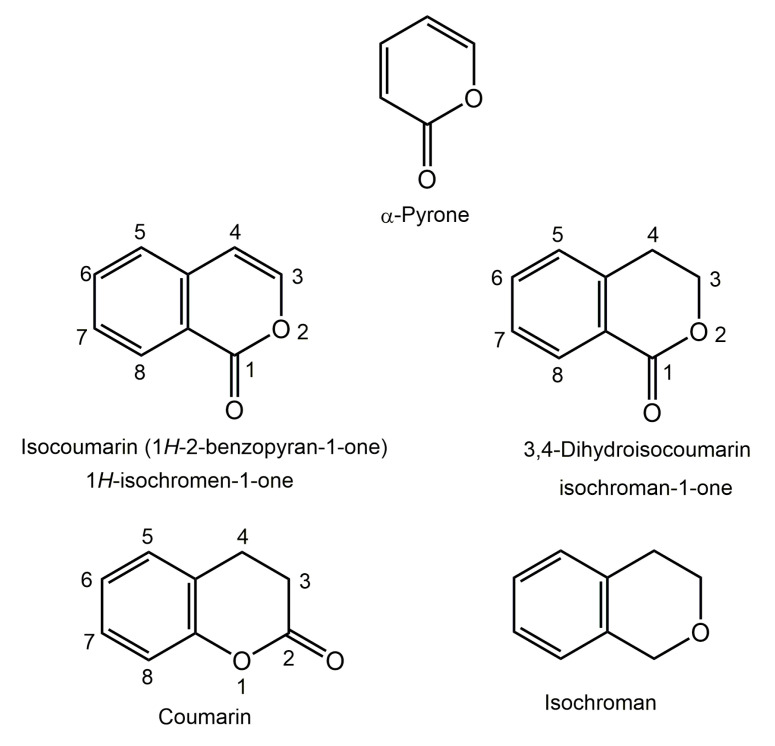
Isocoumarin, 3,4-dihydroisocoumarin, coumarin, and isochroman skeletons.

The search for new metabolites for the agrochemical and pharmaceutical industries is an on-going work that needs continual optimization. Fungi are eukaryotic microorganisms that reside in almost all environmental types in nature where they have key roles in preserving the ecological balance [1,2]. Endophytes primarily inhabit their hosts without causing any harm to the hosts [3,4,5,6]. These endophytic fungi have played pivotal roles in their host’s survival through supplying nutrients and producing plenty of bioactive metabolites to prevent the danger of phytopathogenic bacteria on the host [7,8]. Endophytic fungi have gained loads of attention in natural products chemistry field due to their sustainability to biosynthesize structurally diverse and bioactive molecules, some of which are important agrochemicals and pharmaceuticals [9,10]. Isocoumarins (1*H*-2-benzopyran-1-ones or isochromene derivatives) are a class of biosynthetically, structurally, and pharmacologically intriguing natural products, which are coumarins isomers with a reversed lactone moiety that could possess 6,8-dioxygenated pattern, 3-(un)substituted phenyl ring or 3-alkyl chain (C_1_-C_17_) [11,12]. The oxygenation could exist at one or more of the six free positions of the isocoumarin skeleton. The oxygen atoms may be in the form of ethereal, phenolic, or glycosidic functionalities. Additionally, C-3 substituents are found more commonly on both natural and synthetic isocoumarins derivatives. Substituents that exist on the isocoumarin ring may involve alkyl, halogen, heterocyclic, aryl, or other groups [13]. Furthermore, the saturation of C-3/C-4 in isocoumarins will give 3,4-dihydroisocoumarins (DHICs) analogs (Figure 1).

**Figure 2:**
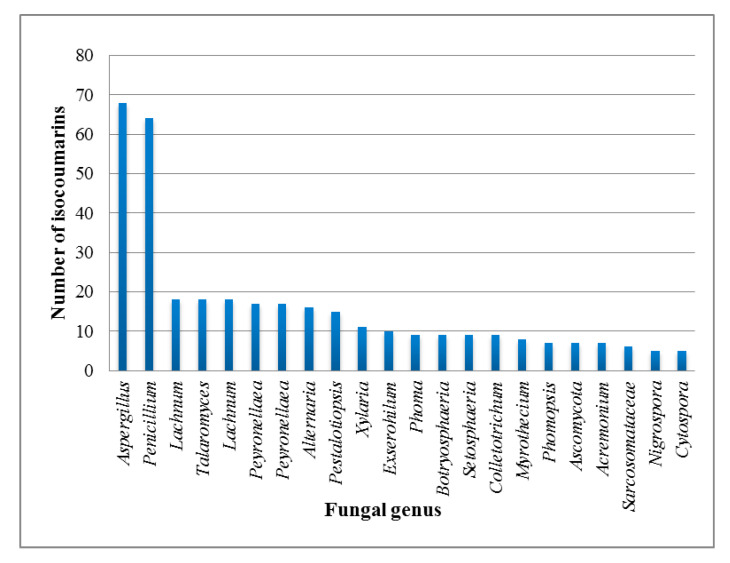
Distribution of isocoumarin derivatives in different fungal genus.

Moreover, isocoumarins and DHICs possess a close relation with isochromans, they are known as isochromen-1-one and isochroman-1-ones, respectively, since the C-1 active methylene in isochromans can be easily oxidized to the related isocoumarins derivatives. Most of the natural isocoumarins and DHICs are given trivial names, which are derived mainly from the name of the species or genus of the host organisms. They have been reported from a broad scope of natural sources, including plants, microbes, marine organisms, bacteria, insects, liverworts, and fungi (e.g., soil, endophytic, and marine fungi) [14,15]. Isocoumarins are considered as important intermediates in the synthesis of a wide range of carbo- and heterocyclic compounds such as isoquinolines, isochromenes, and different aromatic compounds [16]. Thus, isocoumarin framework has been explored in various areas, including drug discovery, pharmaceutical and medicinal chemistry, and organic synthesis [13]. It has been reported that these metabolites possess various bioactivities: antimicrobial, cytotoxic, algicidal, antiallergic, immunomodulatory, antimalarial, plant growth regulatory, and acetylcholinesterase and protease inhibitors [11,17,18,19,20]. This review aims to give a highlight on the naturally occurring isocoumarins derivatives reported from endophytic fungi, focusing on the period from 2000 to July 2019. Herein, 307 naturally occurring isocoumarins derivatives have been listed most of them are reported from *Aspergillus* and *Penicillium* genera (Figure 2). 

The reported fungal isocoumarin derivatives are drawn according to their similarity in the isocoumarin skeleton, as well as nomenclature (Figure 3, Figure 4, Figure 5, Figure 6, Figure 7, Figure 8, Figure 9, Figure 10, Figure 11, Figure 12, Figure 13, Figure 14, Figure 15, Figure 16, Figure 17, Figure 18, Figure 19, Figure 20, Figure 21, Figure 22, Figure 23, Figure 24, Figure 25, Figure 26, Figure 27, Figure 28, Figure 29 and Figure 30). 

It is hoped that by using these figures in conjunction with the trivial name, fungal source, host, and place (Table 1) the readers will be able to locate key references in the literature and gain much understanding of the fascinating chemistry of these metabolites. Many of these derivatives have substituents at C-3, which could be one carbon or more. The majority of them have an oxygen atom at C-8 and some have the C-6 oxygen. Further alkylation or oxygenation may occur at the remaining positions of the isocoumarin skeleton. Isocoumarins with 3,4-, 4,5-, 5,6-, 6,7-, and 7,8-fused carbocyclic rings are reported. Some of the reported derivatives have chlorine (e.g., **9**, **12**, **22**, and **28**–**31**) or bromine (e.g., **23**, **27**, **32**, and **33**) atom at C-5 and/or C-7. Some show sugar moieties such as glucose (e.g., **15**, **77**–**79**, and **151**) and ribose moiety (e.g., **78** and **79**). In addition, some isocoumarins dimers are reported (e.g., **259**, **260**, and **266**–**268**). Moreover, some linked to other moieties such as anthraquinone and indole diketopiperazine (e.g., **285** and **296**) or contain sulphur (e.g., **278** and **279**) or nitrogen (e.g., **269**–**271**) substituents. This review also mentions briefly their isolation, structural characterization, biosynthesis, and bioactivities (Figure 31, Figure 32, Figure 33, Figure 34 and Figure 35, Table 2 and Table 3). Strengthening of their bioactivities may draw the attention of medicinal and synthetic chemists for designing new agents using the known isocoumarins derivatives as raw materials and the discovery of new therapeutic properties not yet attributed to known compounds. The published literature search was conducted over various databases: Web of Science, PubMed, Google Scholar, Scopus, SpringerLink, ACS Publications, Wiley, Taylor and Francis, and Sci-Finder using the keywords (isocoumarin, endophytes, and biological activities). 

## 2. Biosynthesis

Isocoumarin was originated of the acetate-malonate or the polyketide synthase (PKS) pathway [21,22]. Kurosaki et al. stated that **11** is biosynthesized from malonyl-CoA and acetyl-CoA through a pentaketide [23]. 3,4-Dihydro-6-hydroxymellein (**III**) is considered as an intermediate which would be transformed to **11** by *O*-methyltransferase which methylates the 6-OH group of the isocoumarin [23]. The loss of the OH group at C-6 gives rise to mellein [24]. A heptaketide **II**, a longer polyketone chain is implicated in **165** biosynthesis [25] (Figure 31). 

Krohn et al. reported that the existence of a biosynthetic relationship between **56** and **125** [27]. They assumed that the open-chain precursor **A** can be directly closed to a six-membered lactone (pathway **I**) or cyclized after the side chain rotation through the acetyl enol tautomer to produce **56** (pathway **II**) [27] (Figure 32). 

It was postulated that **273** is also derived from the malonate-acetate pathway [28]. The pentaketide (**I**) cyclization and enolization produce **88**. A Claisen condensation occurs between **88** and tetraketide (**II**) to yield **III**. The side chain enolization, along with the hemiketal formation by the side chain ketone carbonyl and C-6 phenolic OH of the isocoumarin nucleus, forms a hemiketal **IV**. Then, the ketal formation and methylation in the side chain by S-adenosyl methionine (SAM) yield **V** and finally **273** [28] (Figure 33). 

**Figure 6:**
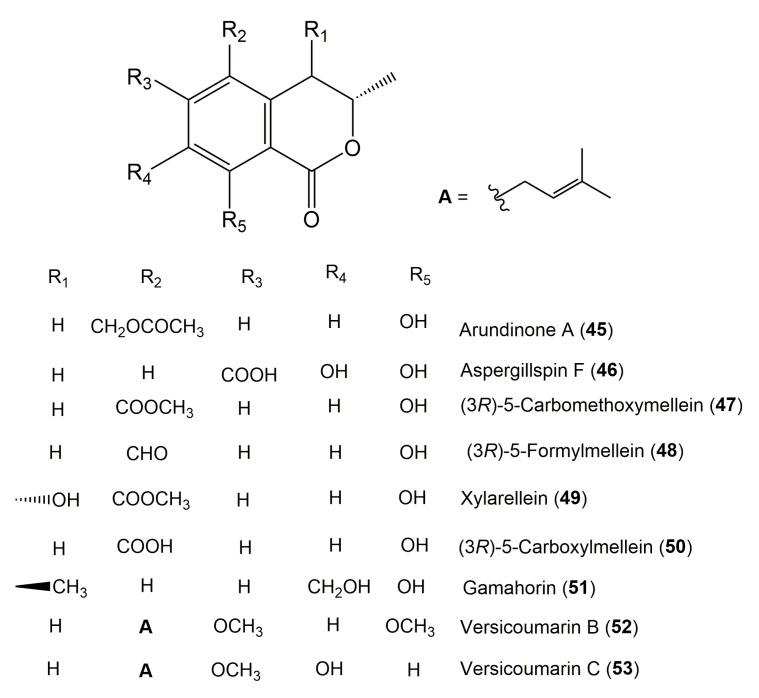
Structures of isocoumarin derivatives **45**–**53**.

**Figure 31:**
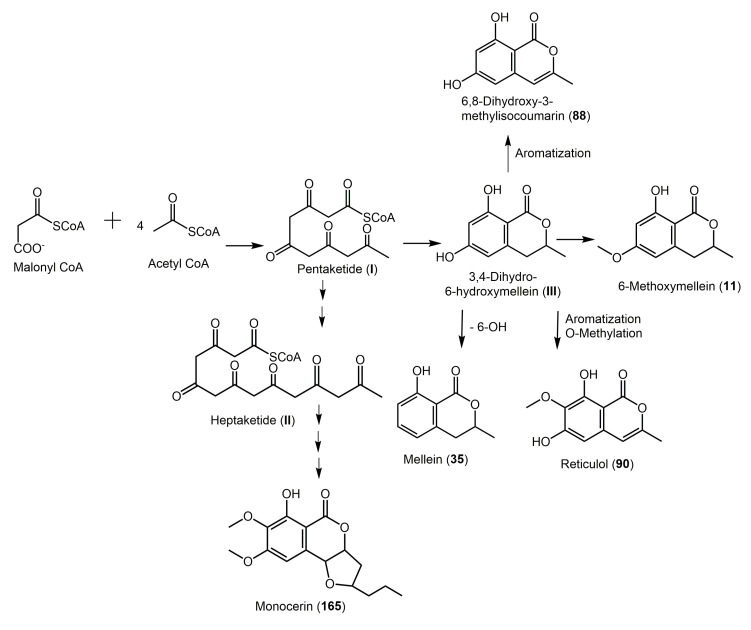
Proposed biosynthetic pathway of **11**, **35**, **88**, **90**, and **165** [21,23,24,25,26].

**Figure 32:**
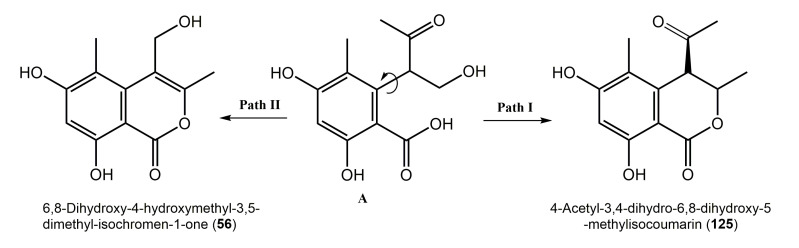
Proposed biosynthetic pathway of **56** and **125** [27].

**Figure 33:**
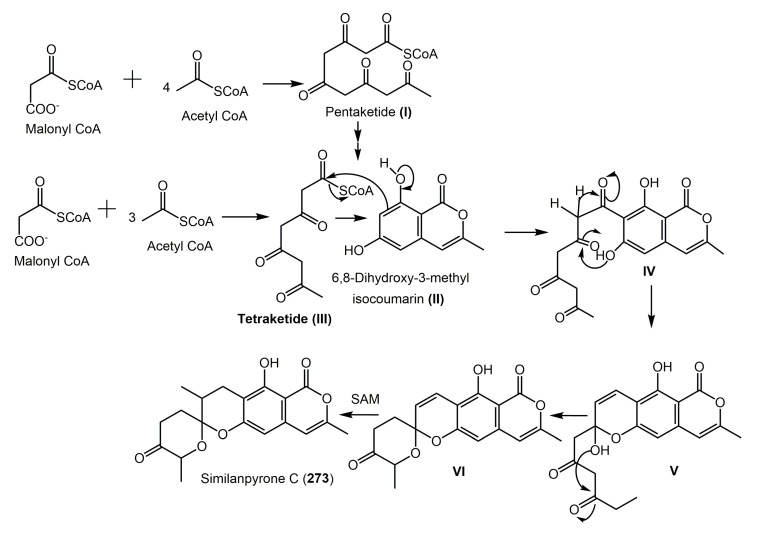
Proposed biosynthetic pathway of **273** [28].

**Figure 34:**
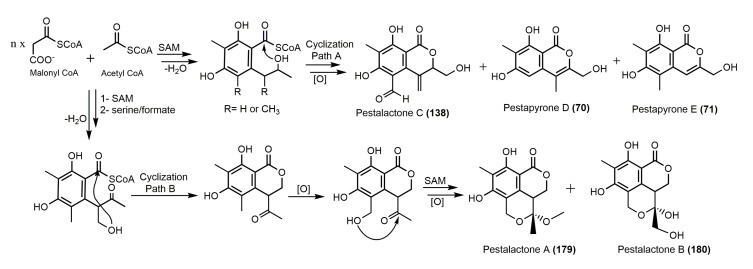
Proposed biosynthetic pathway of **70**, **71**, **138**, **179**, and **180** [22].

Moreover, Song et al. reported that an intramolecular cyclization occurs of a polyketide chain (Path A, Figure 6) [22]. The C-4 substituted derivatives have been resulted from the participation of an additional carbon unit in the cyclization (Path B, Figure 34). 

**Figure 35:**
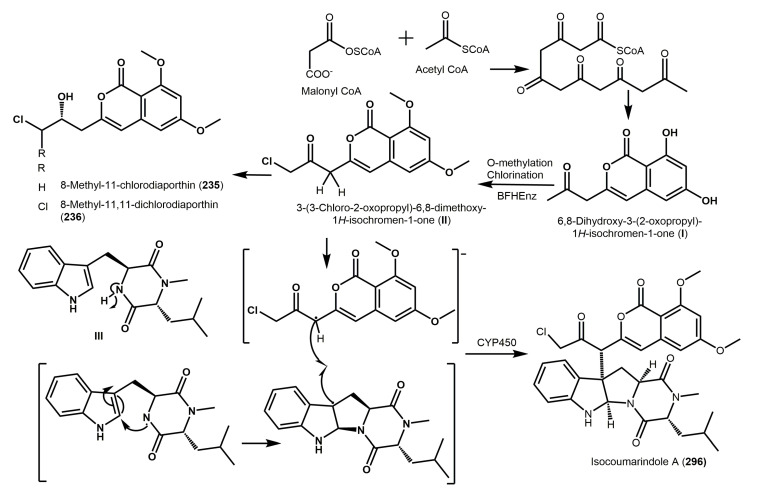
Proposed biosynthetic pathway of **235**, **236**, and **296** [29].

Therefore, the rare isocoumarin derivatives, **179** and **180** biosynthesis differs from those of **70**, **71**, and **138**, in which a carbon moiety (CH_2_OH) from formate or serine took part in the cyclization. Additionally, the 3-unsubstituted derivatives couldn’t be yielded in the biosynthesis of compounds **138** and **70**; due to the C-11 oxidation is usually taking place after the polyketide chain cyclization [22]. Chen et al. postulated the biosynthetic origin of **296**, an isocoumarin-indole diketopiperazine alkaloid (Figure 35) [29]. 

6,8-Dihydroxy-3-(2-oxopropyl)-1H-isochromen-1-one (**I**) was originated from the PKS pathway. It was then chlorinated and *O*-methylated to produce 3-(3-chloro-2-oxopropyl)-6,8-dimethoxy-1H-isochromen-1-one (**II**) by the catalytic effect of a bifunctional hybrid enzyme (BFHEnz). The methyl-carbonyl group of **II** undergoes chlorination and reduction leading to the formation of **235** and **236**, respectively. Then, the hybridization of the diketopiperazine and isocoumarin units by a free radical mechanism, which could be catalyzed by cytochrome P450 giving **296** [29]. 

## 3. Structural Characterization of Isocoumarins Derivatives

Isocoumarins can be characterized by different spectral techniques such as 1D (^1^H, ^13^C, and NOE) and 2D NMR techniques (COSY, HSQC, HMBC, ROESY, and NOESY) combined with other usual methods (chemical synthesis, UV, IR, MS, etc.). However, their spectral data cannot be generalized as the data differ to a wide range relying on the type, position, number, and nature of substituents connected to the core skeleton. Furthermore, these data vary basically due to the variation of the core ring. In the compounds having isocoumarins framework, the lactone carbonyl frequency generally appears in the region 1745–1700 cm^−1^ in the IR. In ^1^H NMR, the C-3 vinylic proton appears at 6.2–7.0 ppm as a singlet or doublet for C_3_-substituted and unsubstituted derivatives, respectively. In ^13^C NMR, the lactone C=O appears in the range from 164 ppm to 168 ppm. In the 3-substituted derivatives, C-4 vinylic proton appears at 6.11–6.7 ppm as a singlet. 3,4-Dihydroisocoumarins derivatives have relatively more complicated ^1^H NMR spectra than isocoumarins due to C-4 and C-3 vicinal coupling and/or C-4 diastereotopic protons geminal coupling. In both derivatives, the 8-OH group appears at 10.0–12.0 ppm due to the hydrogen bonding to the C-1 carbonyl.

Mass spectroscopy is a helpful tool for the identification of these metabolites. The existence of sulfur was evident by the intensity of [M + 2]^+^ ion peak (∼4.5% of the molecular ion peak) [30]. Moreover, the chlorine atom in the structure was characterized by two ion peaks [M + H]^+^ and [M+2H]^+^ in a ratio 3:1 [31,32]. The relative configuration was determined by NOE, NOESY, and ROESY. The circular dichroism (CD) is usually utilized to assess the absolute configuration by comparison of the theoretical and experimental CD spectra [30,31,33]. Besides, the total synthesis provides important information and an additional confirmation for characterization of these metabolites structures. Furthermore, it allows the synthesis of analogs with improved biological efficiencies [11,34,35]. The X-ray structure crystallographic analysis of the crystalline derivatives is another tool for the absolute configuration determination. This technique could not be applied in many cases since the crystals with the required qualifications are not available because most of these metabolites do not crystallize conveniently [20,27,36]. Finally, the assignment of the absolute configuration could be done using Mosher’s method and the differences in chemical shift between the (*R*)- and (*S*)-MTPA were analyzed [33,37].

## 4. Methods of Extraction and Purification of Isocoumarins Derivatives

For the extraction and isolation of isocoumarins, the fungal material was extracted with CH_2_Cl_2_, acetone or EtOAc. The total extracts were partitioned between n-hexane, CHCl_3_, EtOAc, and MeOH or fractionated on SiO_2_ 60 VLC using mixtures of *n*-hexane, EtOAc, and MeOH, or using petroleum ether, CH_2_Cl_2_, and MeOH, respectively [38,39,40]. The fractions were chromatographed over Sephadex LH-20 (CHCl_3_:MeOH 1:1), SiO_2_ CC using gradient elution of CH_2_Cl_2_:MeOH; PE:EtOAc; *n*-hexane:EtOAc [31,36,37,41,42,43] or RP-18 CC using MeOH-H_2_O (8:2, *v*/*v*) [33]. Purifications of compounds were achieved by preparative HPLC using gradient of MeOH:H_2_O or MeCN:H_2_O [31,39,40]; SiO_2_ CC (*n*-hexane:acetone:MeOH, *n*-hexane:acetone gradient or benzene:EtOAc) [44,45,46]; RP-18 CC (H_2_O:MeOH gradient) [47]. Preparative TLC could be used for compounds purification using acetone:petroleum ether (3:7) [41]; CHCl_3_:Me_2_CO:HCO_2_H (97:3:0.01); petrol:CHCl_3_ and CHCl_3_:Me_2_CO [48]; CH_2_Cl_2_:2-propanol (50:1) [49]; PE:EtOAc (1:1) [47]. Isocoumarins derivatives can be purified by recrystallization from CH_2_C1_2_:CH_3_OH or PE:EtOAc until they showed constant melting points. These compounds can be detected on TLC by UV light or spraying reagents (vanillin-sulfuric acid or cerium-molybdenum) [27].

## 5. Biological Activities 

### 5.1. Antimicrobial Activity

The isocoumarins **4** and **5** which were produced by an unidentified *Ascomycete*, separated from *Meliotus dentatus* had a potent antibacterial effect towards *B. megaterium* and *E. coli* with equal partial inhibition (PI) 10 and 9 mm, respectively compared to penicillin (PI 18 and 14 mm, respectively) and tetracycline (PI 18 and 18 mm, respectively). Furthermore, **4** and **5** exhibited prominent antifungal activities toward *Botrytis cinerea*, *Microbotryum violaceum*, and *Septoria tritici* and algicidal activities towards *Chlorella fusca* [50]. Compound **6** was tested against *C. fusca*, *E. coli*, *B. megaterium*, and *M. violaceum* using agar diffusion assay. It showed activity against *C. fusca* (IZD 9 mm), compared to actidione (IZD 35 mm) as well as against *B. megaterium* and *M. violaceum* with IZDs 8 and 6 mm, respectively [43]. Oliveira et al. indicated that **6** exhibited antifungal potential towards *Cladosporium sphaerospermum* and *C. cladosporioides* with detection limit 10 and 5 μg, respectively, whereas **3** showed moderate activity with detection limit 10 and 25 μg, respectively [18]. However, **8** was inactive [18]. The new isocoumarins **23**–**29** produced by Lachnum palmae associated with Przewalskia tangutica showed antimicrobial activities against *Penicillium* sp., *C. neoformans*, *C. albicans*, *S. aureus*, and *B. subtilis* (MICs 10–75 µg/mL), compared to kanamycin and amphotericin B using broth microdilution assay. It is noteworthy that **27** had potential antimicrobial potentials towards all the strains tested (MICs 10–55 µg/mL) [51]. The antifungal effect of **34** separated from *Xylaria* sp. and **36** and **37** separated from *Penicillium* sp. towards *Cladosporium cladosporioides* and *C. sphaerospermum* was assessed using direct bioautography assay [17]. Compounds **37** and **36** showed a promising effect against *C. sphaerospermum* and *C. cladosporioides* (MICs 10.0 and 5.00 μg, respectively), compared to nystatin (MICs 1.0 and 1.0 μg, respectively), while **34** had moderate effect towards *C. sphaerospermum* and *C. cladosporioides* (MICs 25.0 and 10.0 μg, respectively) [17]. Furthermore, compound **65** exhibited moderate effect towards *Vibrio parahemolyticus* and *B. cereus* with MICs 6.25 μM [52]. Furthermore, **35** exhibited only weak potential towards *Botrytis cinerea* (EC_50_ 49.2 μg/mL) [53]. However, **78** and **79** showed no antifungal activity (Conc. 128 μg/mL) toward *C. albicans* (ATCC10231 and ATCC32354) [54]. Compounds **59**, **116**, **124**, and **125** were tested against three fungal organisms: *Eurotium repens*, *Mycotypha rnicrospora*, and *Ustilago violacae*. Only **59** had a moderate potential towards all tested fungi [20]. Compound **132** was evaluated for the antimicrobial effect towards *Cladosporium herbarum*, *Aspergillus niger*, *B. subtilis*, and *Pseudomonas syringae*. The results revealed that **132** exhibited only mild activity towards *B. subtilis* with MIC 25 μg/mL, compared to chloramphenicol (MIC 3.13 μg/mL) [55]. The dihydroisocoumarins **154** and **155** showed selective antibacterial potential against the five pathogenic bacteria *S. epidermidis*, *B. cereus*, *S. aureus*, *Vibrio alginolyticus*, and *E. coli* (MICs 20, 20, 20, 20, and 20 µM, respectively for **154** and 10, 20, 20, 20, and 20 µM, respectively for **155**), compared to ciprofloxacin (MICs 0.30, 0.30, 1.20, 0.60, and 1.25 µM, respectively) [56]. The antibacterial activities of **188**, **189**, and **193** were tested against *B. megaterium*, *B. subtilis*, *E. coli*, *Micrococcus tetragenus*, *Clostridium perfringens*, and MRSA *S. aureus*. Compound **189** had a stronger antibacterial potential (MIC 12.5 μg/mL) against *B. megaterium* than ampicillin (MIC 50 μg/mL). However, the other compounds did not exhibit any activity [57]. Compounds **70**, **71**, **138**, **179**, and **180** which were isolated from *Pestalotiopsis* sp. associated with *Photinia frasery* were evaluated for their antimicrobial activities towards *P. aeruginosa* (ATCC 9027), *S. aureus* (ATCC 25923), *E. coli* (ATCC 25922), *B. subtilis* (ATCC 6633)*,* and *C. glabrata* (ATCC 90030). It is noteworthy that only **138** had a promising antifungal capacity against *C. glabrata* (MIC_50_ 3.49 μg/mL) in comparison to amphotericin B (MIC_50_ 0.25 μg/mL). Whilst, the other metabolites had no activity (Conc. 50 μg/mL) [22]. The antimicrobial activity of **157**–**159** towards different aquatic and human pathogenic bacteria (*E. coli*, *Aeromonas hydrophilia*, *P. aeruginosa*, *Micrococcus luteus*, *Vibrio alginolyticus*, *V. parahaemolyticus*, and *V. harveyi*) and plant pathogenic fungi (*Colletotrichum gloeosprioides* and *Phytophthora parasitica var. nicotianae*) was evaluated. They had broad-spectrum antifungal and antibacterial capacities with MICs ranging from 4 to >64 µg/mL. Compound **157** possessed the highest activities against *P. aeruginosa, E. coli*, *V. harveyi*, *C. gloeosprioides* and *V. parahaemolyticus* (MICs 4 µg/mL), whereas **158** and **159** displayed moderate activities against these microorganisms [58]. Compound **165** (Conc. 500–7.8 μg/mL) which was biosynthesized by *Exserohilum rostratum* isolated from *Bauhinia guianensis* possessed a good activity towards *B. subtilis* (MBC 62.5 μg/mL and MIC 15.62 μg/mL), *E. coli* (MBC 250 μg/mL and MIC 15.62 μg/mL), *P. aeruginosa* (MIC 15.62 μg/mL), *S. Typhimurium* (MIC 31.25 μg/mL), and *S. aureus* (MIC 62.5 μg/mL) [59]. *Microdochium bolleyi* metabolites: **165**, **167**, **169**, and **240** were estimated for their antibacterial, antifungal, and algicidal effects toward *E. coli*, *B. megaterium*, *M. Violaceum*, and *C. fusca* using agar diffusion assay. Compounds **165**, **169**, and **240** inhibited the four tested organisms. It is noteworthy that **167** showed an algicidal potential towards *C. fusca* (IZD 6 mm, actidione IZD 50 mm and nystatin IZD 20 mm) and an antifungal effect against *M. violaceum* (IZD 7 mm, tetracycline IZD 10 mm and actidione IZD 35 mm), but did not have an antibacterial effect [49]. Hussain et al. stated that **197** showed potential antifungal and antibacterial activities against *M. violaceum* (IZD 10 mm) and *B. megaterium* (IZD 5 mm), respectively [42]. Compounds **165**, **167**–**171**, **175**–**177**, and **201** were tested for their antimicrobial potential towards *S. aureus* (CGMCC 1.2465), *B. subtilis* (ATCC 6633), *S*. *pneumoniae* (CGMCC 1.1692), *F. oxysporum* (CGMCC 3.2830), and *E. coli* (CGMCC 1.2340) [60]. Compounds **167** and **177** displayed antifungal potential towards *F. oxysporum* with MICs 20 μg/mL, compared to amphotericin B (MIC 0.63 μg/mL). Furthermore, **201** exhibited significant antibacterial effects against *B. subtilis*, *S. pneumonia*, and *S. aureus* with MICs 20, 10, and 5 μg/mL, respectively in comparison to ampicillin (MICs 1.25, 10, and 0.16 μg/mL, respectively). Furthermore, it had a promising effect towards *E. coli* (MIC 20 μg/mL), compared to gentamicin (MIC 2.5 μg/mL) [60]. Arunpanichlert et al. reported that **213** had a mild antifungal effect against *Cryptococcus neoformans* and *C. albicans* with equal MICs of 200 μg/mL, while **212** was inactive (Conc. 200 mg/mL) [60]. Furthermore, **212** and **213** had a weak antibacterial effect against *B. subtilis* and *S. aureus* (Conc. 50 µg/mL) [61]. Chen et al. reported that the isocoumarins metabolites **54**, **64**–**66**, **84**, **92**–**95**, **150**, **158**, **159**, **212**, **213**, **216**, and **217** which were separated from *Talaromyces amestolkiae*, did not exhibit any antibacterial activity against *Staphylococcus epidermidis, S. aureus, Klebsiella pneumoniae, E. coli,* and *B. subtilis* [62]. Compounds **98**, **99**, **276**–**285**, and **294** which were biosynthesized by *Aspergillus banksianus*, were tested for in vitro antimicrobial activities against *E. coli* (ATCC 25922), *B. subtilis* (ATCC 6633), *Saccharomyces cerevisiae* (ATCC 9763), and *C. albicans* (ATCC 10231). Compounds **283**–**285** displayed weak to moderate activities, whereas the other metabolites had no activity towards any of the tested strains [30]. Compound **232** showed a weak activity towards *B. subtilis* ATCC 6633 and *Trichophyton rubrum* ATCC 28,189 with MIC_80_ 19.7 and 32.0 μg/mL, respectively, compared to penicillin (MIC_80_ 0.9 μg/mL) and fluconazole (MIC_80_ 1.0 μg/mL) [63]. Moreover, compounds **172**, **173**, and **178** isolated from *Setosphaeria* sp. possessed no activity towards *S. aureus, Colletotrichum asianum, C. gloeosporioides, C. acutatum, Pyricularia oryza*, and *F. oxysporum* using broth microdilution technique [64]. Compounds **72**, **188**, **189**, **202**–**205**, **207**–**211**, **231**, **233**, **234**, **286**, and **287** were evaluated against *Agrobacterium tumefaciens*, *E. coli*, *Ralstonia solanacearum, S. aureus*, *Bacillus thuringensis*, *Xanthomonas vesicatoria*, and *Pseudomonas lachrymans*. The metabolite **188** showed moderate inhibitory effect towards *A*. *tumefaciens, R*. *solanacearum,* and *S*. *aureus* with MICs 16 μM, while **189** and **234** had weak inhibition (MICs 32 μM) against *A. tumefaciens* and *R. solanacearum*, respectively [65]. The isocoumarins **88**–**90** and **272** exhibited no activities (MIC > 256 μg/mL) towards *P. aeruginosa, B. subtilis, S. aureus, E. coli,* and *C. albicans* [48]. The antifungal activity of **220** and **290** was assessed using the broth dilution method. Compound **290** showed significant antifungal capacity towards *Rhizoctonia solani* (MIC 6.25 μg/mL), compared to carbendazim (MIC 6.25 μg/mL) and moderate effect against *Colletotrichum musae* (MIC 25 μg/mL), whereas **220** exhibited weak activities toward these two fungi (MICs 150 μg/mL). However, none of these metabolites was active towards *Fusarium graminearum* and *Penicillium italicum* (MICs > 200 μg/mL) [66]. Compounds **188**, **193**–**196**, **232**, and **299** biosynthesized by *Alternaria alternata* were tested against *B. subtilis* ATCC 6633, *S. aureus* ATCC 25923, *T. rubrum* ATCC 28189, and *C. albicans* ATCC 24433. Compound **194** ([(−)- and (+)] displayed moderate effects against *S. aureus* (MICs 15.4 and 17.1 μg/mL, respectively), compared to penicillin MIC 1.2 μg/mL) whereas **193**, **195** ([(−)- and (+)], and **196** had no prominent effect. These results demonstrated that the 2-OH acetylation could enhance the activity towards *S. aureus*, however, the enantiomeric difference may have a negligible influence. Furthermore, (+)-**194** and (+)-**195** showed promising potential towards *C. albicans*, while (−)-**194** and (−)-**195** had less activities, suggesting the difference in the antifungal potentials among the different enantiomers. Moreover, **188** possessed the highest activity towards *B. subtilis* (MIC 8.6 μg/mL). Whilst **232** had no activity. [63] Bai et al. stated that compounds **186**, **187**, **244**, **245**, and **248** had no antibacterial potential towards *E. coli*, MRSA *S. aureus*, *S. aureus*, *B. cereus*, *Vibrio alginolyticus*, and *V. parahaemolyticus* using microplate assay [67]. Chen et al. reported that **296** had mild activity against *C*. *albicans* (MIC 32.0 μg/mL), compared to caspofungin (MIC 0.03 μg/mL) [29]. Compounds **1**, **2**, and **16** demonstrated a significant potential against *Trichophyton longifusus* and *Microsporum canis* (% inhibition 45, 70, and 55, respectively and 50, and 50, and 70, respectively), compared to miconazole, (% inhibition 70 and 98.4, respectively), whereas they were inactive towards *C. albicans*, *Fusarium solani*, *C. glabrata*, and *Aspergillus flavus* using agar tube dilution technique [68]. The antimicrobial potential of **237** and **238** separated *Trichoderma harzianum* was investigated towards *B. subtilis*, *E. coil*, *C. albicans*, *S. aureus*, and *P. aeruginosa* using well diffusion technique. They had a weak inhibitory effect against *E. coli* (MICs 32 μg/mL), compared to chloramphenicol (MIC 4 μg/mL), whereas they were inactive towards the other tested microorganisms [69]. Compounds **46** and **73** new isocoumarins reported from *Aspergillus* sp. SCSIO 41,501 derived from marine gorgonian *Melitodes squamata* were inactive against *E. coli* and *B. subtilis* using disc diffusion technique [70]. The new isocoumarins metabolites, **13**, **106**, and **107** isolated *Botryosphaeria ramosa* were assessed for their antifungal potential towards *F. oxysporum*, *P. italicum*, and *F. graminearum*. Compounds **13** and **107** demonstrated a high inhibitory potential against *F. graminearum* and *F. oxysporum* (MICs 223 and 223 μM and 211.7 and 105.8 μM and respectively) in comparison to triadimefon (MICs 510.7 and 340.4 μM, respectively), whereas **106** had a significant activity against *F. oxysporum* (MIC 112.6 μM) and a weak effect towards *F. graminearum* (MIC 900 μM) [71]. Xu et al. reported that **145** displayed moderate activities against *P. aeruginosa* (MIC 50 μg/mL) and *E. coli* (MIC 12.5 μg/mL), compared to ciprofloxacin (0.078 and 0.625 μg/mL, respectively) [72]. The anti-bacterial activity of **100**, **220**, **239**, **274**, and **275** towards *K. pneumonia*, *S. epidermidis*, *E. coli*, *S. aureus*, and *B. subtilis* was assessed. Only **220** had activities against *B. subtilis* and *S. aureus* (MICs 25 μg/mL). While **100**, **239**, **274**, and **275** (Conc. 25 μg/mL) did not exhibit any activity against the tested strains [73]. Compounds **162**–**164**, novel dihydroisocoumarins isolated *Geotrichum* sp., associated with *Crassocephalum crepidioides* had been tested for the antifungal activity towards *C. albicans* using colorimetric technique [74]. Compounds **162** and **164** had a weak antifungal potential towards *C. albicans* (IC_50_s 13 and 33 μg/mL, respectively), compared to amphotericin B (IC_50_ 0.01 μg/mL). Whilst **163** had no activity [74]. Compounds **51**, **146**, and **147** possessed antibacterial potential towards *B. subtilis* (MICs 100, 50, and 25 μg/mL, respectively) and *S*. *aureus* (MICs 100, 25, and 25 μg/mL, respectively), compared to ciprofloxacin (MICs 0.25 and 0.13 μg/mL, respectively). However, they were inactive against *E. coli*, *C. albicans*, *C. parapsilosis*, and *Cryptococcus neoformans* [75]. The new isocoumarins **261**–**266** which were produced by *Aspergillus* sp. 085,242 separated from *Acanthus ilicifolius* roots exhibited no antibacterial capacity towards *Staphylococcus epidermidis*, *S. aureus*, *E. coli*, *B. subtilis*, and *Klebsiella pneumoniae* [76]. Compound **297** had a moderate antifungal capacity towards *Botrytis cinerea*, *S. sclerotiorum*, *Phytophthora capsici*, *Fusarium graminearum*, *and F. moniliforme* (inhibition rates 18.8, 39.0, 13.7, 24.0, and 31.6%, respectively) [77]. The isocoumarins **151–153**, **214**, **215**, and **295** had no activity against *B. cereus*, *E. coli*, *S. albus*, *S. aureus*, *B. subtilis*, *Kocuria rhizophila*, *Micrococcus tetragenus*, *Vibrio anguillarum*, and *V. parahemolyticus*, whereas **153** possessed weak activity (MIC 12.5 μM) against *S. aureus* compared to ciprofloxacin (MIC 0.160 μM) [52]. Compounds **42**, **304**, and **306** reported from *Seltsamia galinsogisoli* were assessed for the antimicrobial effect towards *S. aureus*, *P. aeruginosa*, *B. subtilis*, *B. cereus*, and *K. pneumonia* [78]. Compound **306** had selective activity against *S. aureus* (MIC 32 μg/mL), whereas **42** and **304** exhibited weak effects [78].

### 5.2. Cytotoxic Activity

The cytotoxic activities of isocoumarins have been assessed towards various cancer cell lines using various assays and the most active compounds have been listed in Table 3.

The cytotoxicity of **203**, **219**, and **221** reported from *Ampelomyces* sp. associated with *Urospermum picroides* was assessed against L5178Y (mouse lymphoma) cells using MTT assay. Interestingly, **203** had a strong cytotoxic activity with EC_50_ 7.3 μg/mL [39]. Compound **289** isolated from *Trichoderma* sp. was moderately active against HepG2 and MCF-7 cell lines (IC_50_s 39.6 and 17.8 μg/mL, respectively) by MTT assay compared with epirubicin (IC_50_s 5.3 and 5.2 μg/mL, respectively) [79]. The cytotoxicity of **165** and **168** towards CHAGO, BT474, HepG2, SW-620, and KATO-3 carcinomas was estimated using MTT colorimetric assay [46]. None of these metabolites was cytotoxic (Conc. 20 μg/mL) [46]. Compounds **160**, **161**, **165**, and **166** were evaluated for the cytotoxic activity towards HuCCA-1, HepG2, MOLT-3, and A549 [80]. They were weakly cytotoxic (IC_50_ 115.3–153.0 μM). Interestingly, **166** possessed selective cytotoxic activity (IC_50_ 23.7 μM) toward HepG2 cell line, compared to etoposide (IC_50_ 15.8 μM) [80]. Arunpanichlert et al. stated that **61**, **212**, and **213** which were separated from *Pestalotiopsis* sp., had no activity towards MCF-7, noncancerous Vero cell, and human oral cavity cancer [41]. Furthermore, the isocoumarin metabolites, **3**, **88**, **131**, **165**, and **293** biosynthesized by *Botryosphaeria* sp. KcF6 did not have cytotoxic capacity towards MCF-7, K562, U937, A549, HeLa, HL-60, DU145, MOLT-4, and BGC823 cancer cell lines [36]. Compounds **204**, **222**–**230**, **258**, **267**, and **268** did not exhibit cytotoxic potential towards MCF-7, HepG2, A549, HEK293T, and HeLa cell lines [31]. Ebada et al. reported that **259** and **260** (Conc. 10 μg/mL) had cytotoxic potential against L5178Y cell line with % growth inhibition 33 and 13, respectively using MTT assay [81]. The isocoumarins **98**, **99**, **276**–**285**, and **294** biosynthesized by *Aspergillus banksianus* were tested for in vitro activity against NS-1 cells. Compounds **283**–**285** displayed weak to moderate activity, whereas the other metabolites had no activity [30]. Compound **100** possessed no cytotoxicity against A2780 cell line [82]. Compounds **78** and **79**, isocoumarin ribonic glycosides biosynthesized by *Daldinia eschscholzii*, had no obvious activity (IC_50_ > 40 μg/mL) towards SMMC-7721, HL-60, A-549, SW-480, and MCF-7 using the MTT assay [54]. Compounds **183** and **184** were obtained from *Talaromyces* sp. that inhabited *Cedrus deodara*, showed cytotoxic potential towards HEP-1, A-549, THP-1, HCT, and PC-3 cell lines (% inhibition 23, 15, 54, 44, and 23%, respectively for **183** and 3, 35, 40, 35, and 34%, respectively for **184**) [83]. Phomasatin (**141**) showed no cytotoxicity (IC_50_ > 50 µM) against Molm 1, HL-60, and PC-3 cell lines using MTT assay [84]. Compounds **154** and **155** had no cytotoxic potential against HeLa, MCF-7, and A549 cells (IC_50_ > 50 µM) [56]. Compounds **157**–**159**, dihydroisocoumarin derivatives reported from *Penicillium simplicissimum* were examined against *Artemia salina* (brine shrimp lethality). They showed brine shrimp lethality with LD_50_ 7.7, 36.4, and 18.6 µg/mL, respectively compared to colchicine (LD_50_ 16.5 µg/mL) [58]. Alternariol (**188**) was reported to prevent cell proliferation by intervention with the cell cycle. The MTT assay results of the related derivatives from *Alternaria* sp. indicated that all alternariol derivatives demonstrated activity toward the L5178Y, except for **190** and **192**. Compound **188** was the highly active metabolite (EC_50_ 1.7 μg/mL), whereas **189** and **191** had activity with EC_50_s 7.8 and 4.5 μg/mL, respectively. However, **193** was inactive [40]. Compounds **269**–**271** obtained from *Alternaria tenuis* were tested for their in vitro cytotoxicity against A375-S2 and HeLa cells using MTT assay. Compounds **269** and **271** had a potent effect with IC_50_s 0.1 and 0.02 mM and 0.3 and 0.05 mM, respectively. However, **270** displayed only weak activity (IC_50_ 0.4 mM) to HeLa cells [85]. Fusariumin (**298**) a new isocoumarin derivative (Conc. 10 µg/mL) which was isolated from *Fusarium* sp. displayed a significant growth inhibitory potential against *A. salina* with mortality rate 78.2% [86]. Wang et al. reported that **188**, **193**–**196**, **232**, and **299** were inactive (Conc. 50.0 μM) towards U2OS and HepG2 using the MTT method [63]. Furthermore, compounds **186**, **187**, **244**, **245**, and **248** had no activity towards HeLa, HepG2, and A549 cell lines [67]. Furthermore, **46** and **73** possessed no cytotoxicity towards MCF-7, HepG2, and HL60 cell lines using MTT assay [70]. Compound **296** possessed significant cytotoxicity towards AsPC-1 and MIA-PaCa-2 cell lines with IC_50_s 5.53 and 1.63 μM, respectively, in comparison to gemcitabine (IC_50_s 20.10 and 1.02 μM, respectively) [29]. Compounds **100**, **220**, **239**, **274**, and **275** were assessed for their cytotoxic effects towards HepG2, MDA-MB-435, HCT116, MCF10A, and H460 cell lines [73]. It is noteworthy that **274** had a selective cytotoxic potential towards HepG2, MDA-MB-435, MCF10A, and H460 (IC_50_s 43.70, 5.08, 11.34, and 21.53 μM, respectively) comparable to epirubicin (IC_50_s 0.32, 0.26, 0.13, 0.12 μM, respectively), whereas **275** showed a selective effect against MCF10A and MDA-MB-435 (IC_50_s 21.40 and 4.98 μM, respectively). However, other metabolites (Conc. 50 μM) did not affect all tested cell lines [73]. The new isocoumarin metabolite, **20** isolated from *Bruguiera sexangula* root-associated fungus *Penicillium sp*. 091,402 exhibited moderate cytotoxicity potential towards K562 (IC_50_18.9 μg/mL) [87]. Huang et al. reported that **142** prohibited HepG2 and Hep-2 cells growth (IC_50_s 55 and 52 μg/mL, respectively) using MTT method [88]. The cytotoxic potential of **252** isolated *Aspergillus versicolor* was estimated towards A549, NB4, PC3, SHSY5Y, and MCF7. Interestingly, it had a higher activity towards MCF7 and A549 with IC_50_s 8.0 and 5.8 μM, respectively [89]. The new isocoumarin, **108** isolated from *Arthrinium sacchari* displayed a weak cytotoxic effect towards HUVECs and HUAECs (IC_50_ 70.8 and 277.1 μM, respectively), compared to Ki8751 (IC_50_s 1.0–2.0 μM) using MTT assay [90]. Compound **35** did not have a cytotoxic effect against MRC-5 and AGS cell lines [91]. Moreover, **6** possessed no cytotoxic capacity against NCIH460, MCF-7, and A375-C5 cell lines using the protein binding dye SRB method [92]. The cytotoxic abilities against A549, NB4, MCF7, SHSY5Y, and PC3 tumor cell lines of **38**, **52, 53**, **127**, **137**, **251**, and **301** were tested [93]. Compound **251** exhibited a high cytotoxic effect towards MCF7 and A549 cells (IC_50_ 3.8 and 4.0 μM, respectively), in comparison to taxol (IC_50_ 0.1 and 0.02 μM, respectively), while the other compounds had moderate cytotoxic capacities towards some of the tested cell lines with IC_50_s less than 10 [93]. Compounds **23**–**29** were assessed for their cytotoxic capacities towards HepG2, HL-60, and SGC-7901 using MTT method. Only **27** had weak cytotoxicity towards HepG2 (IC_50_ 42.8 µM) [51]. The cytotoxic effect of **111** and **112** towards NB4, SHSY5Y, A549, MCF7, and PC3 using MTT assay was evaluated [94]. They had moderate to weak inhibitory capacities against some tested human tumor cell lines (IC_50_s 2.8–8.8 µM) [94]. Penicimarins **151**–**153**, **214**, **215**, and **259** had no cytotoxic activity towards HL-60, HeLa cell, A-549, and K562 cell lines [52]. 

### 5.3. Antioxidant Activity 

Choudhary et al. reported that **16** had a scavenging potential against DPPH (IC_50_ 159 μM) in comparison to PG (IC_50_ 30 μM) and BHA, also had a powerful XO inhibitory potential (IC_50_ 243 μM), in comparison to BHA (IC_50_ 591 μM) and PG (IC_50_ 628 μM), while **2** showed a weak XO inhibitory potential (IC_50_ 707 μM) [68]. Compounds **47**, **50**, and **134** exhibited no antioxidant capacities in the DPPH assay [95]. However, **90** showed a moderate DPPH scavenging activity with an IC_50_ 58 μg/mL, compared to BHA (IC_50_ 5.5 μg/mL) [96]. Compounds **161** and **166** scavenged DPPH with IC_50_s 23.4 and 16.4 μM, respectively in comparison to ascorbic acid (IC_50_ 21.2 μM) [80]. Furthermore, they prohibited the formation of superoxide anion radical with IC_50_ values of 4.3 and 52.6 μM, respectively in the XXO assay in relation to allopurinol (IC_50_ 3.0 μM), whereas **165** showed no radical scavenging activities [80]. Moreover, they did not suppress the generation of superoxide anions induced by TPA in the differentiated HL-60 cells. Compounds **160**, **165**, and **166** showed excellent ORAC antioxidant activity (14.4, 10.8, and 11.5 ORAC units, respectively) [80]. Pang et al. reported that **172**, **173**, and **178** possessed no radical scavenging activity against DPPH [64]. The radical scavenging capacities of **261**–**266** were tested using DPPH. Only compounds **261** and **263** exhibited weak activity with EC_50_s 125.0 and 130.0 μM, respectively compared to vitamin C (EC_50_ 35.0 μM) [76]. The ability of compounds **72**, **188**, **189**, **202**–**205**, **207**–**211**, **231**, **233**, **234**, **286**, and **287** to regulate Nrf2, that complies to the oxidative stress by binding to ARE in the gene’s coding promoter for antioxidant enzymes and protein for the synthesis of glutathione using ARE-driven luciferase reporters in HepG2C8 cells was evaluated. Compounds **209**, **210**, and **233** (a dose 10 μM) produced a significant induction of luciferase 1.93–2.95 folds more than DMSO (blank control), whereas tBHQ (positive control) invigorated the luciferase activation with 4 folds more than DMSO at a dose (50 μM) [65]. Compounds **80**, **88**, **118**, **119**, **303**, and **307** isolated from *Penicillium coffeae* were tested for their DPPH scavenging activities. Only **302** had a moderate effect (IC_50_ 656 μM), compared to BHT (IC_50_ 59 μM), whereas the rest compounds had no activities (IC_50_ > 900 μM) [47].

### 5.4. α-Glucosidase, Acetylcholinesterase (AChE), and Protein Kinase Inhibitory Activities

α-Glucosidase is a carbohydrase, which is secreted from the epithelium of the small intestine [97,98]. It catalyzes the degradation of carbohydrates into α-glucose thus elevating the blood glucose level [99,100]. One of the therapeutic approaches for treating diabetes is to retard glucose absorption via inhibiting this enzyme. *α*-Glucosidase inhibitors slow down the digestion and absorption of carbohydrates by competitive blocking of the *α*-glucosidase activity [101]. Consequently, the postprandial blood glucose concentration is reduced [99,100,101]. Therefore, many efforts have been made to identify α-glucosidase inhibitors from natural sources.

Compounds **54**, **64**–**66**, **84**, **92**–**95**, **150**, **158**, **159**, **212**, **213**, **216**, and **217** were tested for the α-glucosidase inhibitory capacity [62]. Compounds **84**, **95**, **93**, and **212** exhibited promising inhibitory activities (IC_50_s 89.4, 17.2, 36.4, and 38.1 μM, respectively), better than acarbose (IC_50_ 958.3 μM). The activity of **150**, **213**, **216**, and **217** was five-fold more than that of acarbose. Compounds **65**, **94**, **66**, **159**, and **158** displayed moderate inhibitory activity with IC_50s_ 315.3, 302.6, 417.8, 266.3, and 431.4 μM, respectively. Moreover, **54**, **65**, and **66** had weak activity, whereas the other isocoumarins had activities similar to that of acarbose [62]. Compounds **114** and **115** biosynthesized by *Aspergillus* sp. were assessed for their in vitro α-glucosidase inhibitory capacities. Compound **114** exhibited more efficacy than that of acarbose (IC_50_ 553.7 μM) with IC_50_ 90.4 μM, whereas **115** was moderately active [38]. The new isocoumarins, **261**–**266** were assessed for the α-glucosidase inhibitory potential in comparison to acarbose (IC_50_ of 628.3 μM) [76]. Compounds **262**, **265**, and **266** showed moderate inhibitory effects with IC_50_s 87.8, 52.3, and 95.6 μM, respectively [76]. Cui et al. reported that **203**, **205**, and **206** had a moderate inhibitory activity with IC_50_s 343.7, 392.5, and 538.7 μM, respectively, compared to acarbose (IC_50_ 815.3 μM) [33]. The α-glucosidase inhibitory capacities of **204**, **222**–**230**, **258**, **267**, and **268** which were biosynthesized by *Penicillium commune* were evaluated. Compounds **225**, **227**, **228**, and **258** possessed powerful activity (IC_50_ 38.1–78.1 μM) than acarbose (IC_50_ 478.4 μM). However, compounds **223**, **224**, and **229** were moderately active (IC_50_s 102.4–158.4 μM) [31]. Compound **103** which was separated from *Xylariaceae* sp. isolated from *Quercus gilva* stem, possessed α-glucosidase inhibitory potential with IC_50_ 41.75 μg/mL, compared to quercetin (IC_50_ 4.80 μg/mL) [102]. The in vitro glucose consumption assay of **78** and **79** (Conc. 20 μg/mL) showed no activity with DMEM-induced 3T3 fibroblasts in the anti-diabetic model [54]. The α-glucosidase inhibitory activity of **76**, **81, 82, 85**–**88**, **93**, and **95** which are reported from *Myrothecium* sp. was investigated. Compounds **76**, **81**, **85**, and **87** exhibited inhibitory potential towards the *Saccharomyces cerevisiae* expressed human-sourced α-glucosidase recombinant with IC_50_s 0.37, 0.32, 0.036, and 0.026 mM, respectively compared to acarbose (IC_50_ 0.47 mM) [103]. Compounds **165**, **167**, **174**, and **218** biosynthesized by *Leptosphaena maculans* did not show any inhibitory effects against α-glucosidase [104]. 

Acetylcholinesterase (AChE), an enzyme that catalyzes acetylcholine (ACh) hydrolysis leading to a decrease in the levels of ACh in the brain [105]. Thus, appears to be a critical element in the development of neurodegenerative diseases such as Alzheimer’s disease (AD) and dementia. The most suitable therapeutic approach for treating AD and other forms of dementia is to restore ACh levels by inhibiting AChE [106]. Compounds **3**, **6**, and **8** were evaluated for their AChE inhibitory activities. Compound **6** had a moderate AChE inhibitory potential with a limit of detection 30.0 μg, whereas **3** and **8** were inactive (limit of detection over 100 μg) [18]. Compounds **34**, **36**, and **37** showed weak inhibition of AChE with a limit of detection 10 μg in a TLC-based AChE inhibition assay [17]. Only **34** displayed moderate AChE inhibitory activity (limit of detection 3.0 μg) compared to galantamine (MICs 1.0 μg) [17]. 

Protein kinases are enzymes that catalyze the transfer of a phosphate group from a high energy molecule such as adenine triphosphate (ATP) to a specific amino acid. They play important roles in regulating many cellular functions, including survival, proliferation, motility, apoptosis, as well as DNA damage repair and metabolism [107]. Some of them are commonly activated in cancer cells and known to play roles in tumorigenesis [108]. Protein kinases inhibitors are anticipated to be a source of potential therapeutic targets for treating various human disorders such as neoplastic and neuroinflammatory diseases [107,108]. The isolated alternariol derivatives from *Alternaria* sp. were assessed for their inhibitory activities against 24 protein kinases. Interestingly, alternariol (**188**) and its derivatives **189**–**191** prohibited protein kinases: Aurora A, ARK5, Aurora B, IGF1-R, b-RAF, VEGF-R2, FLT3, VEGF-R3, SAK, and PDGF-Rbeta with IC_50_ below 1 × 10^−6^ g/mL. Moreover, **193** exhibited activity with an IC_50_ 1 × 10^−5^ g/mL or less towards the various tested kinases [40].

### 5.5. Anti-Inflammatory Activity

Compounds **3**, **88**, **131**, **165**, and **293** were assessed for their COX-2 inhibitory activities. Only 293 exhibited significant inhibitory activity with an IC_50_ 6.51 μM [36]. The anti-inflammatory activities of **203**, **208**, **220**, **221**, **231**, **288**, **289**, **291**, and **292** were assessed against NO production in the LPS-stimulated mouse macrophage RAW 264.7 [37]. Compound **292** had a potent inhibitory capacity (IC_50_ 15.8 µM), whereas **220**, **221**, and **291** were weakly active compared to indomethacin (IC_50_ 37.5 µM), while the other compounds had no inhibitory potential (IC_50_ > 100 µM) [37]. Annulohypoxylomarin A (7) derived from the endophytic fungus *A. truncatum* did not affect (IC_50_ > 100 μM) the production of TNF-α, IL-12 p40, and IL-6 in LPS-stimulated bone marrow-derived dendritic cells [45]. Compounds **186**, **187**, **244**, **245**, and **248** did not affect the NO production in LPS-induced RAW 246.7 mouse macrophages [67]. Compounds **19** and **36** biosynthesized by *Xylaria cubensis* associated with *Litsea akoensis* leaves were assessed for their capacities to prohibit IL-6 and NO production in LPS-activated RAW 264.7 cells. It is noteworthy that **36** had IL-6 inhibitory potential with IC_50_ 9.4 μM [109]. Compounds **23** and **27** had moderate inhibitory effects on the production NO in LPS-induced RAW 264.7 cells (IC_50_ 26.3 and 38.7 µM, respectively) with no observed toxicities at 50 µM [51]. 

### 5.6. Anti-Mycobacterial, Antiplasmodial, Antiviral, and Insecticidal Activities

Compounds **204**, **222**–**230**, **258**, **267**, and **268** (Conc. 50 μM) were tested for their *Mycobacterium* protein tyrosine phosphatase B (MptpB) inhibitory activity [31]. Compound **225** had MptpB inhibitory effect (IC_50_ 20.7 μM), compared to oleanolic acid (IC_50_ 22.1 μM), however, the other compounds possessed weak or no inhibitory effects at the same concentration [31]. Arunpanichlert et al. reported that compounds **61**, **212**, and **213** showed no activity against *Mycobacterium tuberculosis* [41]. Compounds **162** and **163** showed weak antituberculous potential against *M. tuberculosis* H27Ra (IC_50_s 25 and 50 μg/mL, respectively), in comparison to kanamycin sulfate and isoniazid (IC_50_s 0.050 and 2.5 μg/mL, respectively) using MABA, whereas **164** had no activity [74]. The antiplasmodial activity against the multidrug-resistant K1 strain of *Plasmodium falciparum* of **165** and **168** was estimated using microculture radioisotope technique [46]. Compound **165** showed antiplasmodial activity (IC_50_ 0.68 μM). However, its 11-hydroxy analog (**168**) had a 10-fold lower activity, suggesting that the existence of the OH group in the *n*-propyl chain decreases the activity [46]. Compounds **61**, **212**, and **213** had no antimalarial activity towards *P. falciparum* [41]. Furthermore, **6** and **35** had no antimalarial potential against K1 strain of *P. falciparum* [110,111]. Compounds **98**, **99**, **276**–**285**, and **294** were tested for in vitro activity against *Tritrichomonas fetus* (KV-1). The results revealed that **283**–**285** displayed weak to moderate activity, whereas the other metabolites had no activity [30]. Compounds **162** and **163** showed significant antimalarial potential with IC_50_s 4.7 and 2.6 μg/mL, respectively, compared to chloroquine diphosphate (IC_50_ 0.16 μg/mL) towards *P. falciparum* (K1, multi-drug resistant strain) using microculture radioisotope technique [74]. However, **163** was inactive [74]. Compound **11** separated from the endophytic fungus *Phoma* sp. exhibited antiviral activity against influenza A virus (A/Puerto Rico/8/34, H1N1) with IC_50_ 20.98 µg/mL, compared to arbidol (IC_50_ 0.15 µg/mL) [26]. The anti-tobacco mosaic virus (anti-TMV) activities **39**, **67**–**69**, **96**, **97**, and **137** (Conc. 20 μM) produced by *P. oxalicum*, isolated from *Nicotiana sanderae* leaves was screened using the half-leaf method [44]. The results showed that **67** had the highest activity (% inhibition 25.4). While other compounds possessed weak activity with an inhibition rate ranging from 11.3 to 18.9% [44]. Duan et al. mentioned that **40** and **41** (Conc. 20 μM) had anti-TMV capacities (inhibition rates 18.6 and 21.8%, respectively) using the half-leaf technique [112]. The anti-TMV activities of **38**, **52, 53**, **127**, **137**, **251**, and **301** were evaluated using the half-leaf method at a concentration 20 μM. Interestingly, **251** showed a high anti-TMV potential (inhibition rate 28.6%), compared to ningnanmycin (inhibition rate 31.5%), whereas the other metabolites had a moderate activity (inhibition rates 15.6–22.0%) [93]. Oryzaeins A (**111**) and B (**112**), new isocoumarin derivatives isolated from Aspergillus oryzae were tested for their anti-TMV utilizing the half-leaf assay at Conc. 20 µM. The results revealed that **111** and **112** had moderate anti-TMV potential (inhibition rates 28.4 and 30.6%, respectively) [94]. Antifeedant activities of **134** and **135** were evaluated against larvae of *Spodoptera littoralis* using glass-fiber discs. Compound **135** possessed activity at 100 ppm with feeding index 42.1, whereas **134** did not affect the feeding (feeding index 30.1) [55]. The new isocoumarins, **186**, **187**, **244**, **245**, and **248** displayed a growth inhibitory activity toward *Helicoverpa armigera Hubner* (IC_50_s 200, 200, 200, 100, and 100, μg/mL, respectively), compared to azadirachtin (IC_50_ 25 μg/mL) [67].

### 5.7. Other Biological Activities

The antischistosomal activity of **6** and **35** was estimated against *Schistosoma mansoni* adult worms. It is noteworthy that **6** and **35** (Conc. 50 and 200 μg/mL, respectively) caused 100% death of the parasites, compared to praziquantel (Conc. 12.5 μg/mL) [113]. Compounds **6** and **131** produced by *Neofusicoccum parvum,* showed phytotoxic activity on tomato plants with symptoms ranging from slight to drastic wilting of leaves [114]. Nakashima et al. reported that **87**, **109**, **148**, and **149** isolated from *Houttuynia cordata* leaves associated fungus *Tubakia* sp. ECN-111 had no agonistic activity for a liver X-receptor and peroxisome proliferator-activated receptor-C in a luciferase reporter gene assay [115]. Compounds **160**, **161**, **165**, and **166** did not prohibit aromatase (CYP19) enzyme (IC_50_ 15.3–16.9 μM) [80]. Compound **300** produced by *Penicillium* sp. possessed moderate inhibitory activity on the *Arabidopsis thaliana* seeds germination [116].

## 6. Conclusions 

Endophytic fungi have been emerged as a new area for the discovery of new pharmaceutical candidates and continue to be a prosperous pool of bioactive and structurally unique metabolites. The isocoumarins and 3,4-dihydroisocoumarins ring system is present in nature with an enormous spectrum of bioactivities, extending from antibacterial to anticancer. According to this review, the distinctive pharmacological significance of these metabolites initiates much research to be done and still going on towards the advancement and synthesis of their derivatives. The inactivity reported for some derivatives in former studies could be due to prejudice in the evaluation experiments. Further assessments of these metabolites with wider spectrum screening systems are endorsed, which may lead to the invention of their interesting activities.

## Figures and Tables

**Figure 3:**
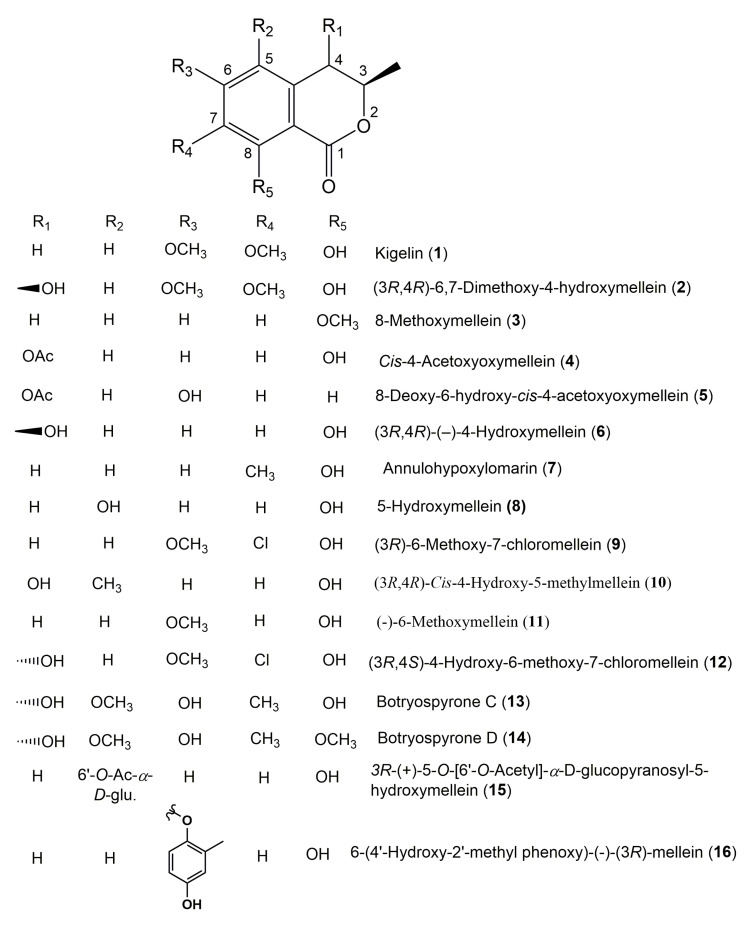
Structures of isocoumarin derivatives **1**–**16**.

**Figure 4:**
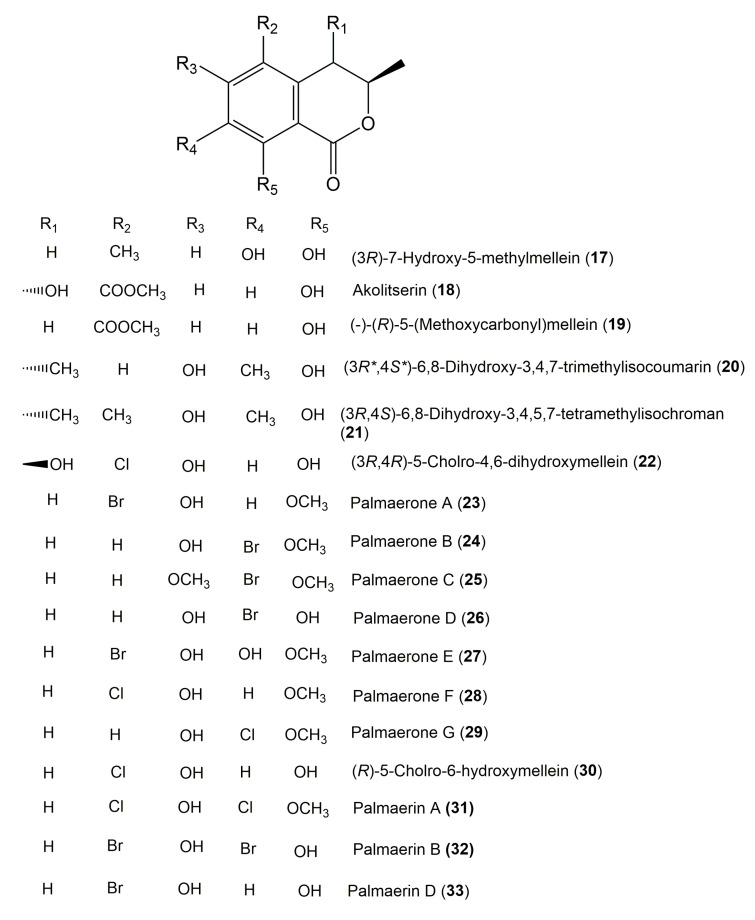
Structures of isocoumarin derivatives **17**–**33**.

**Figure 5:**
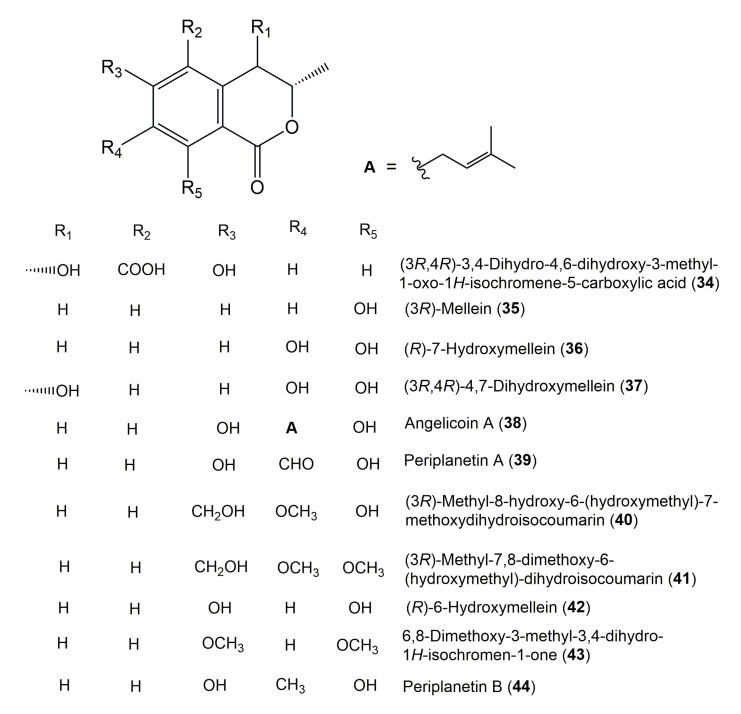
Structures of isocoumarin derivatives **34**–**44**.

**Figure 7:**
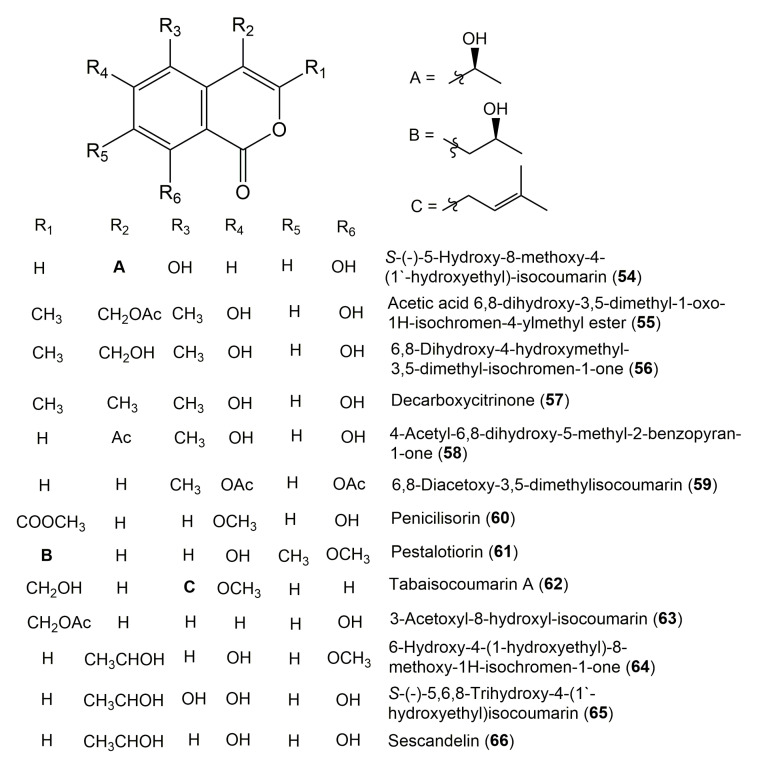
Structures of isocoumarin derivatives **54**–**66**.

**Figure 8:**
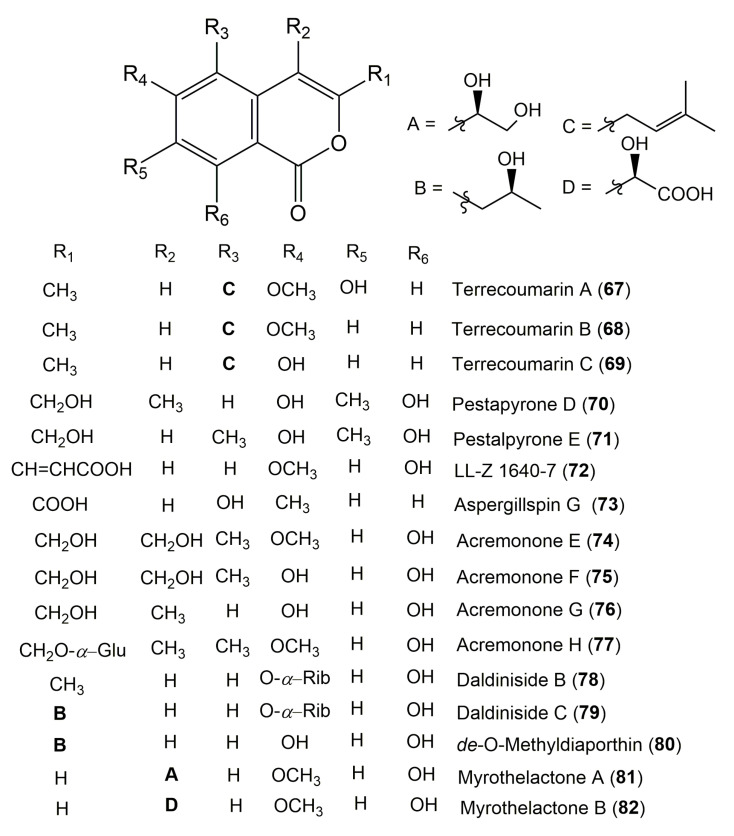
Structures of isocoumarin derivatives **67**–**82**.

**Figure 9:**
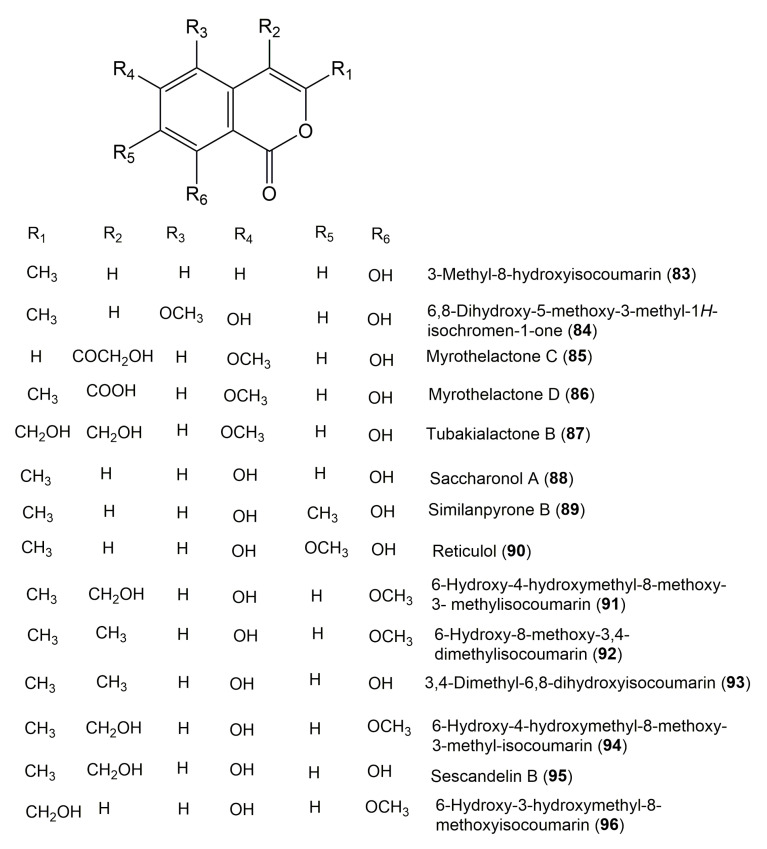
Structures of isocoumarin derivatives **83**–**96**.

**Figure 10:**
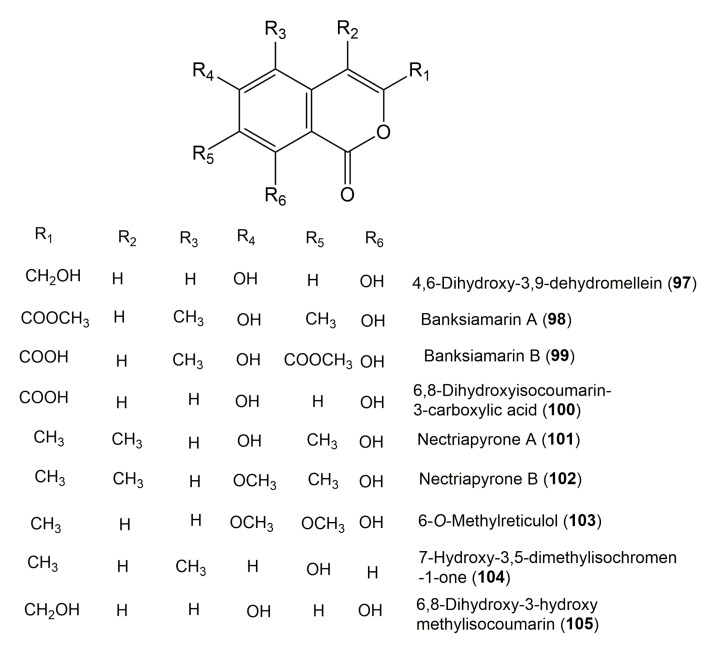
Structures of isocoumarin derivatives **97**–**105**.

**Figure 11:**
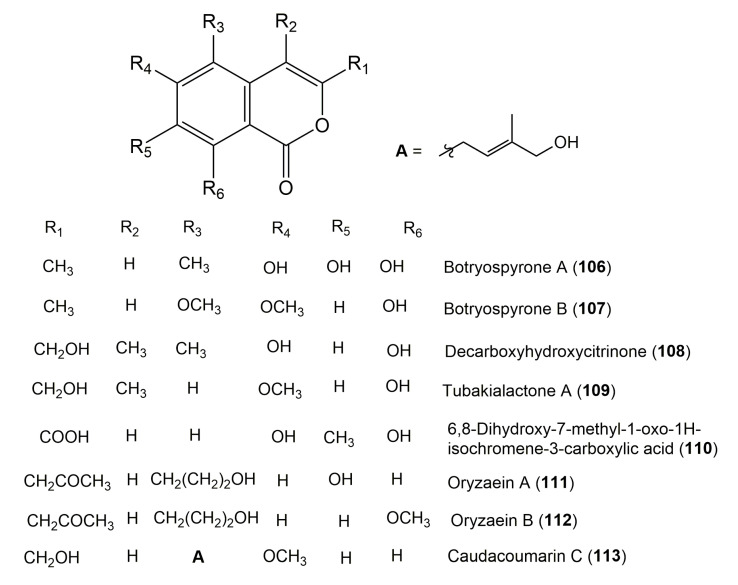
Structures of isocoumarin derivatives **106**–**113**.

**Figure 12:**
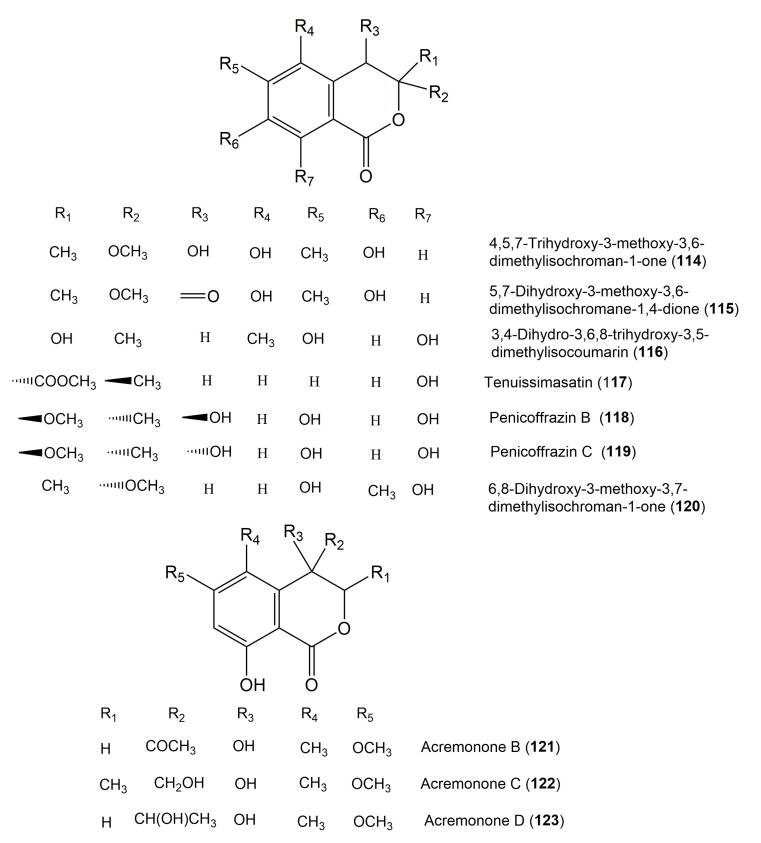
Structures of isocoumarin derivatives **114**–**123**.

**Figure 13:**
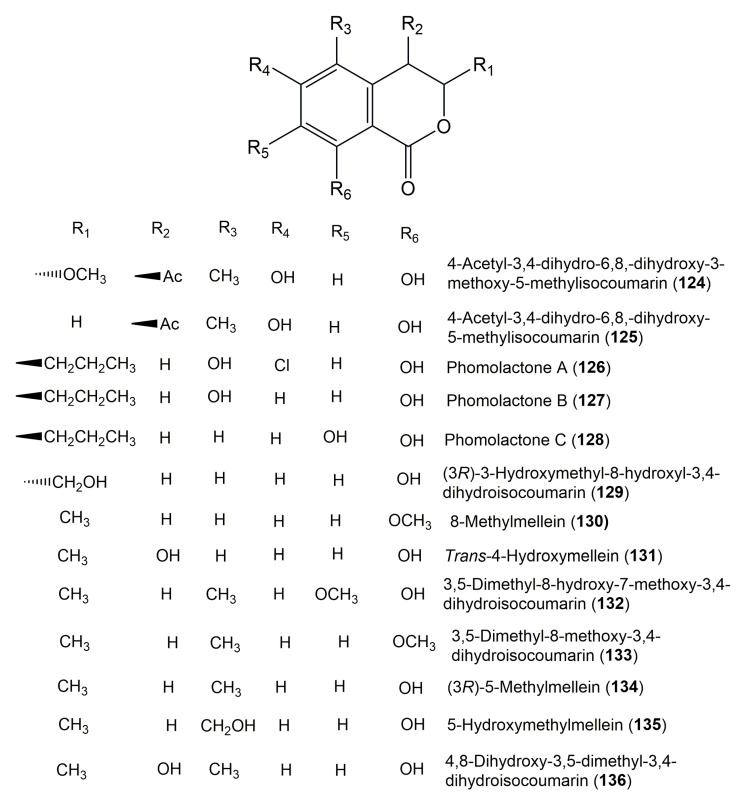
Structures of isocoumarin derivatives **124**–**136**.

**Figure 14:**
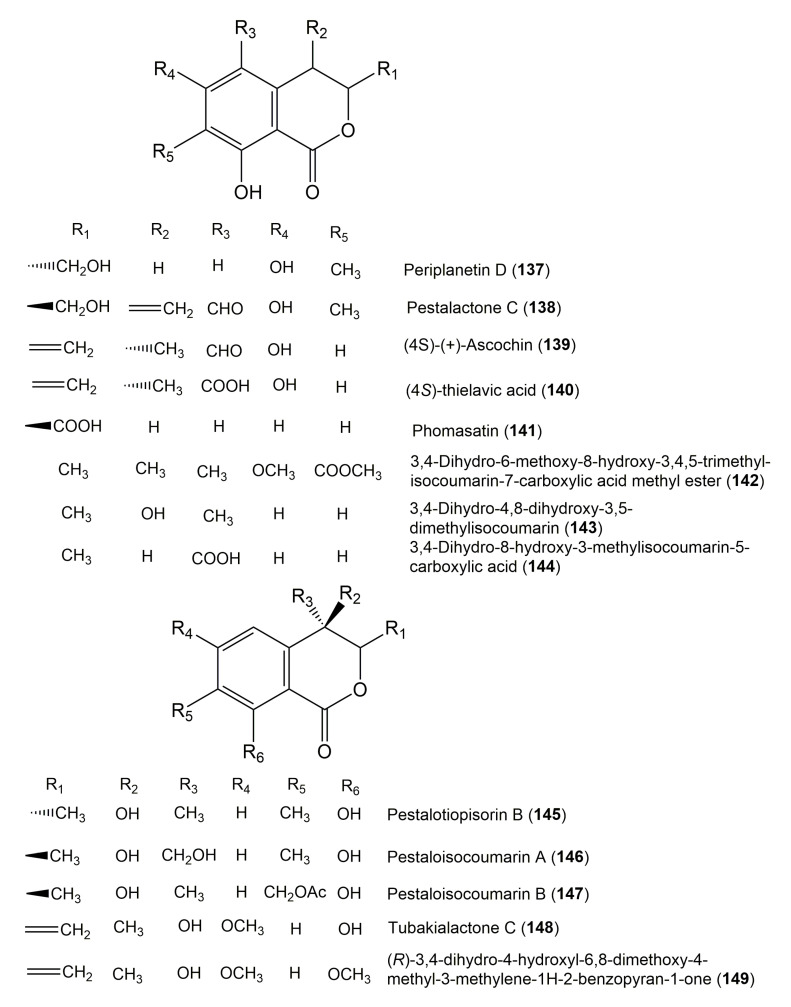
Structures of isocoumarin derivatives **137**–**149**.

**Figure 15:**
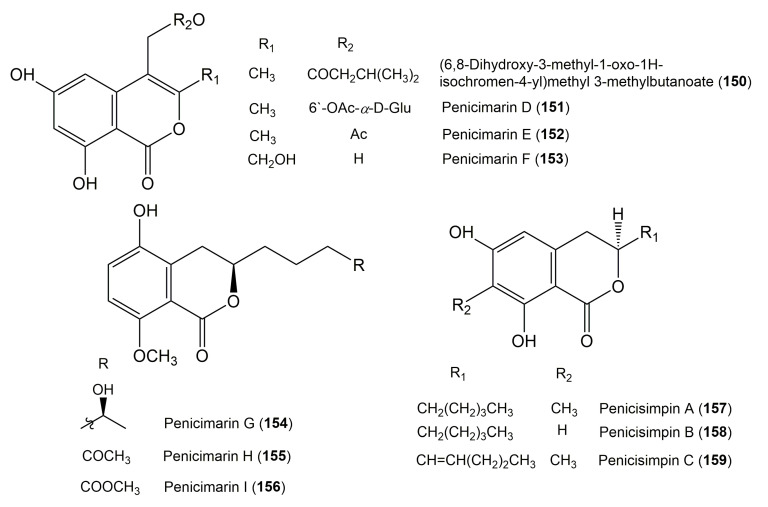
Structure of isocoumarin derivatives **150**–**159**.

**Figure 16:**
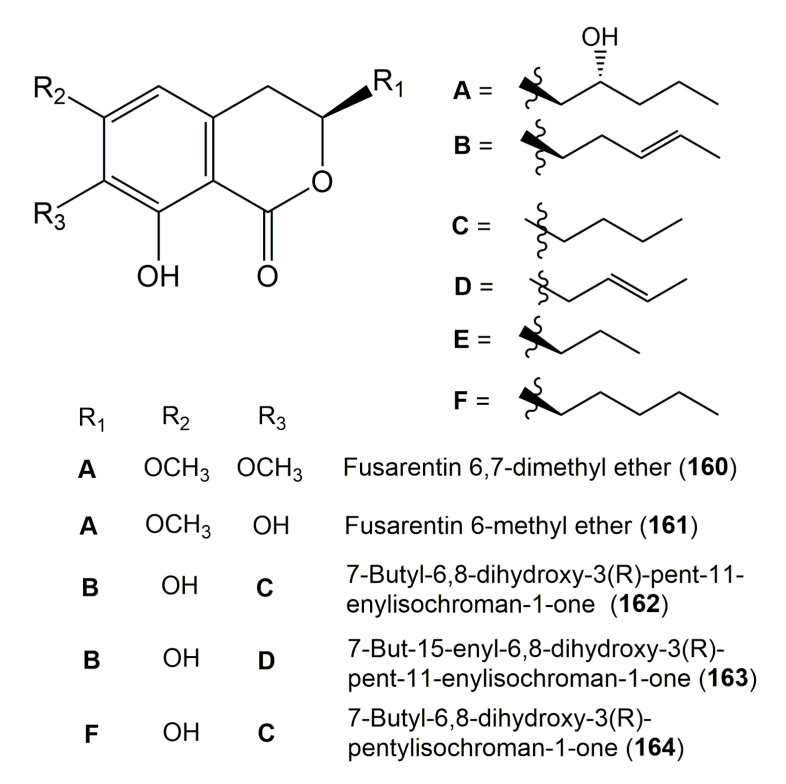
Structures of isocoumarin derivatives **160**–**164**.

**Figure 17:**
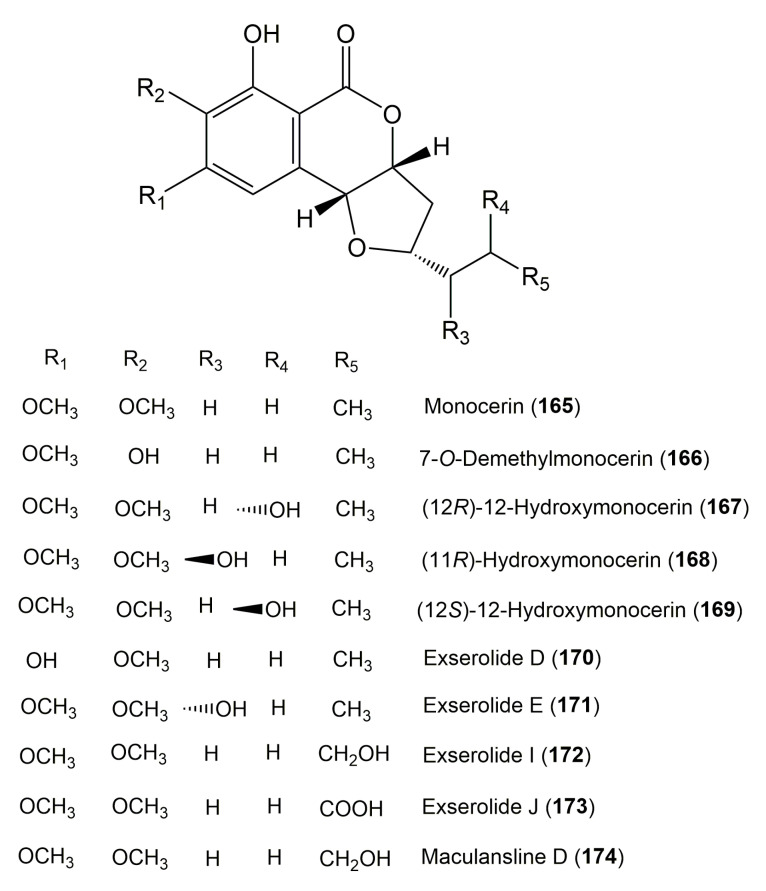
Structures of isocoumarin derivatives **165**–**174**.

**Figure 18:**
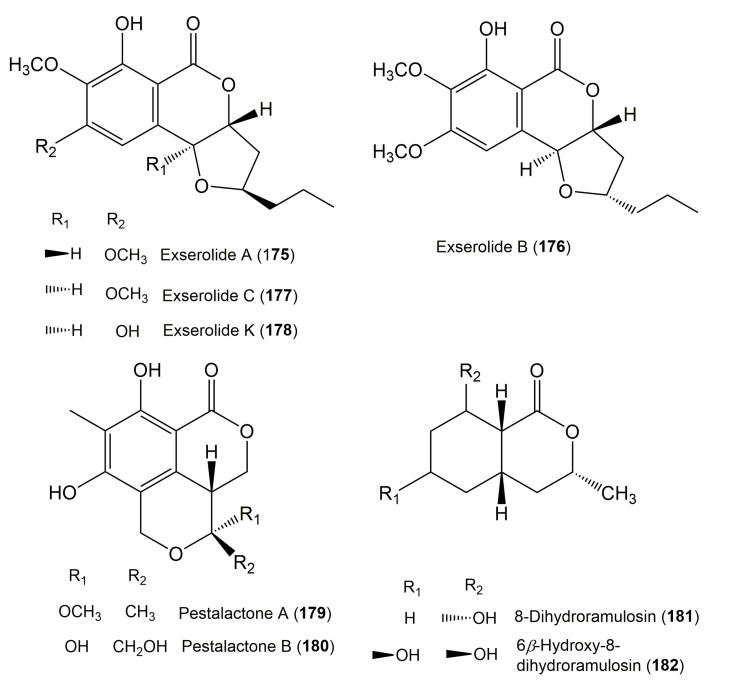
Structures of isocoumarin derivatives **175**–**182**.

**Figure 19:**
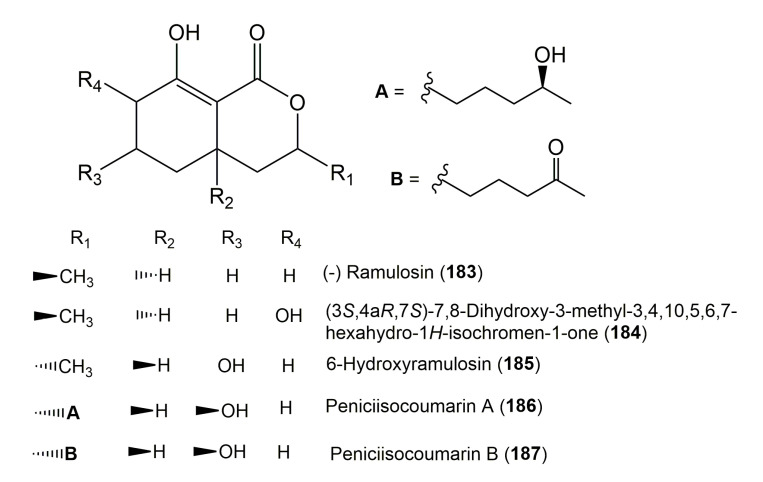
Structures of isocoumarin derivatives **183**–**187**.

**Figure 20:**
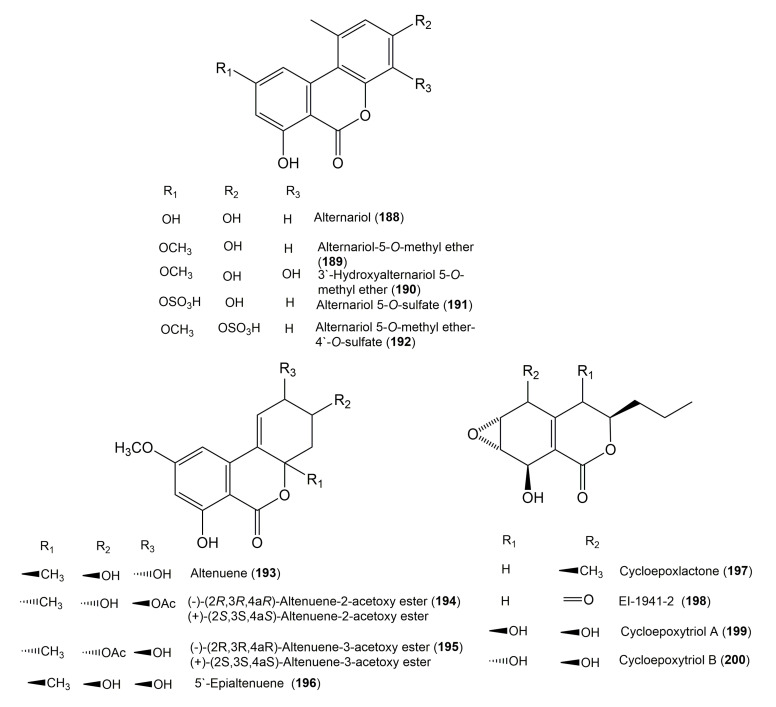
Structures of isocoumarin derivatives **188**–**200**.

**Figure 21:**
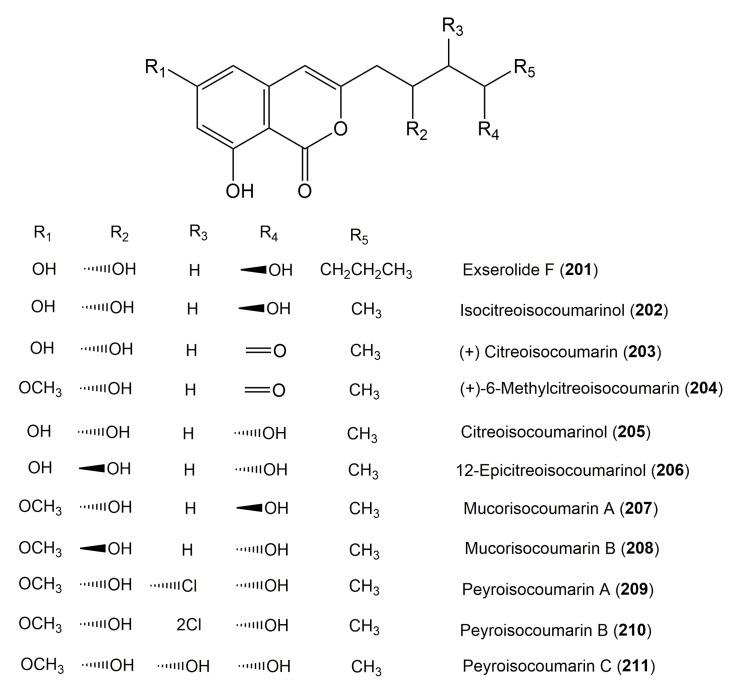
Structures of isocoumarin derivatives **201**–**211**.

**Figure 22:**
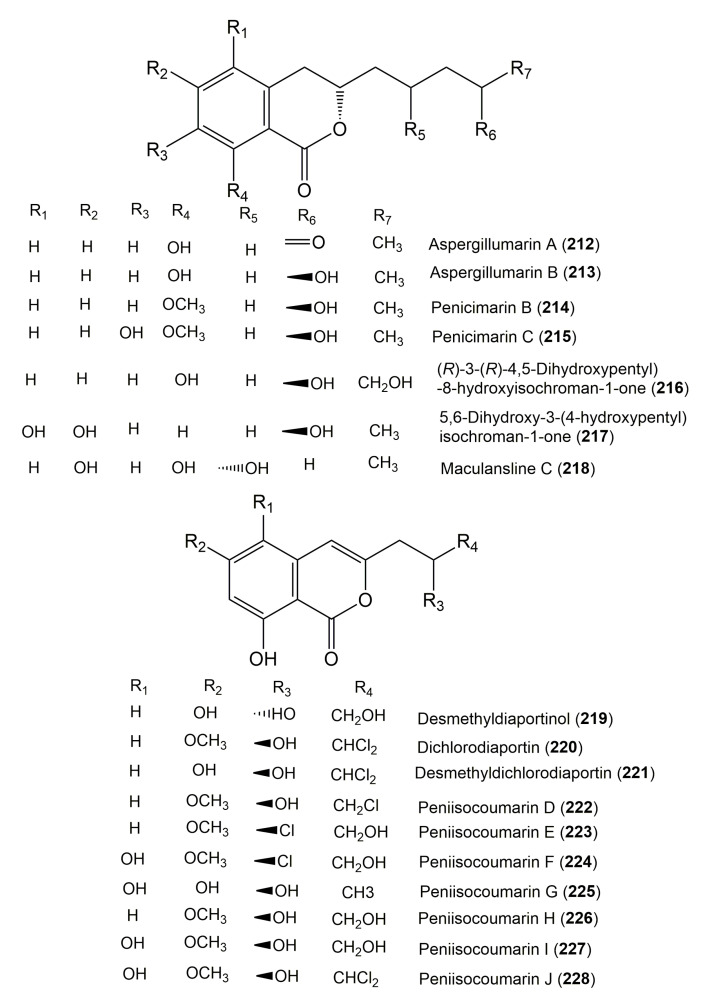
Structures of isocoumarin derivatives **212**–**228**.

**Figure 23:**
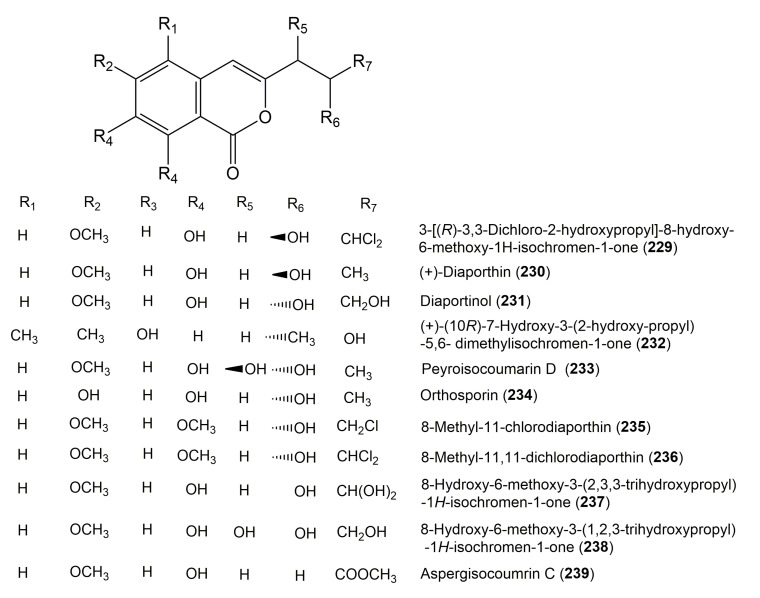
Structures of isocoumarin derivatives **229**–**239**.

**Figure 24:**
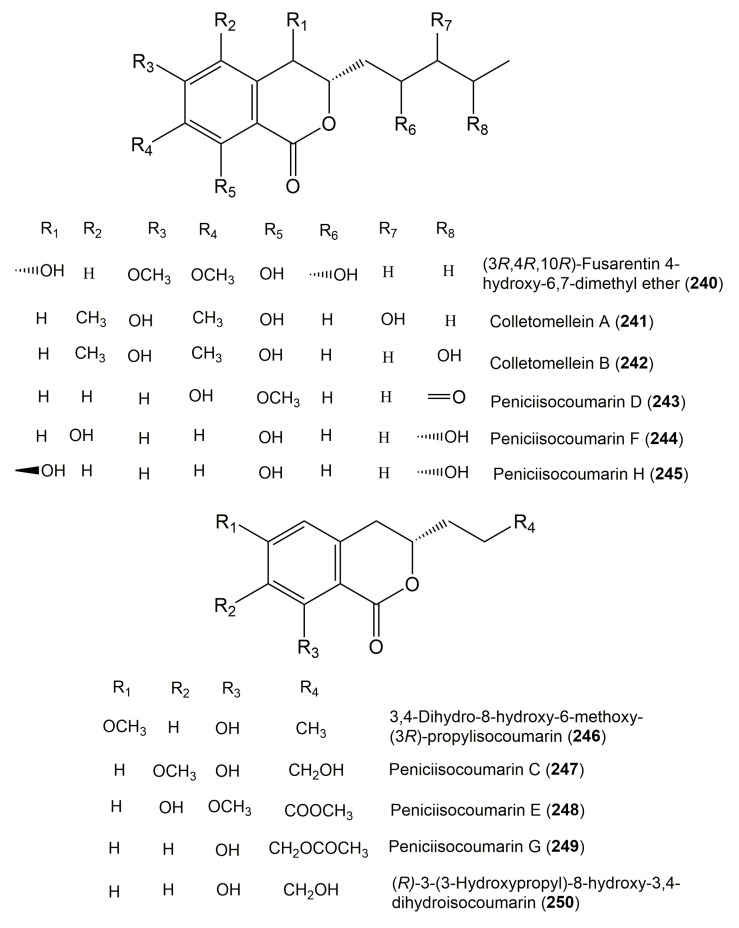
Structures of isocoumarin derivatives **240**–**250**.

**Figure 25:**
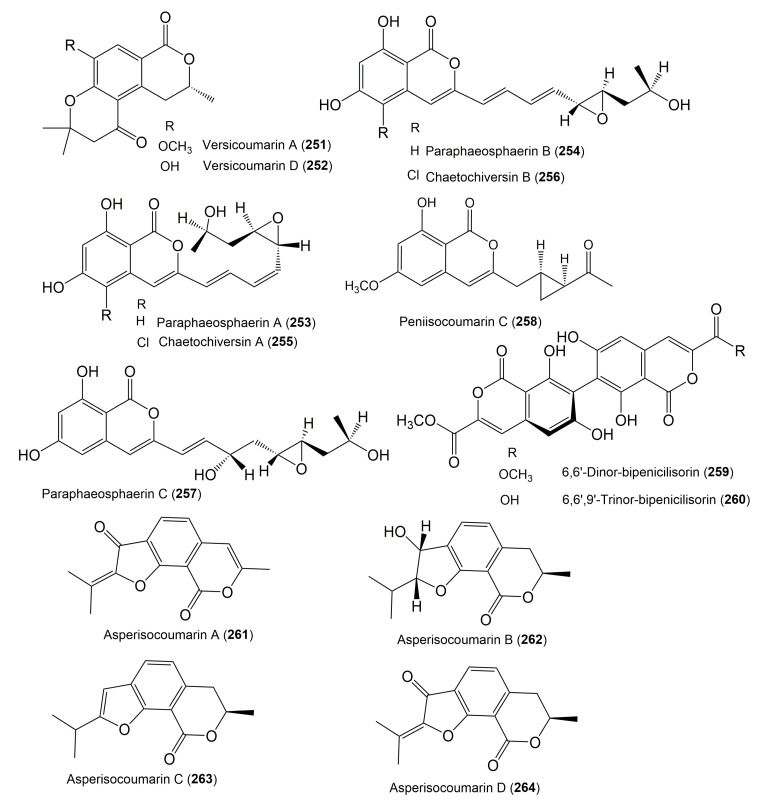
Structures of isocoumarin derivatives **251**–**264**.

**Figure 26:**
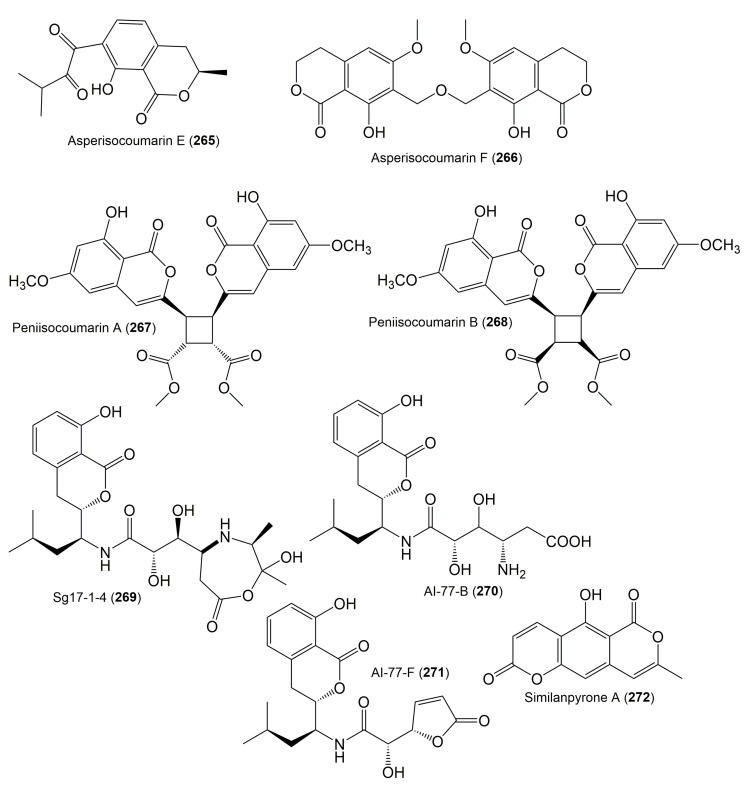
Structures of isocoumarin derivatives **265**–**272**.

**Figure 27:**
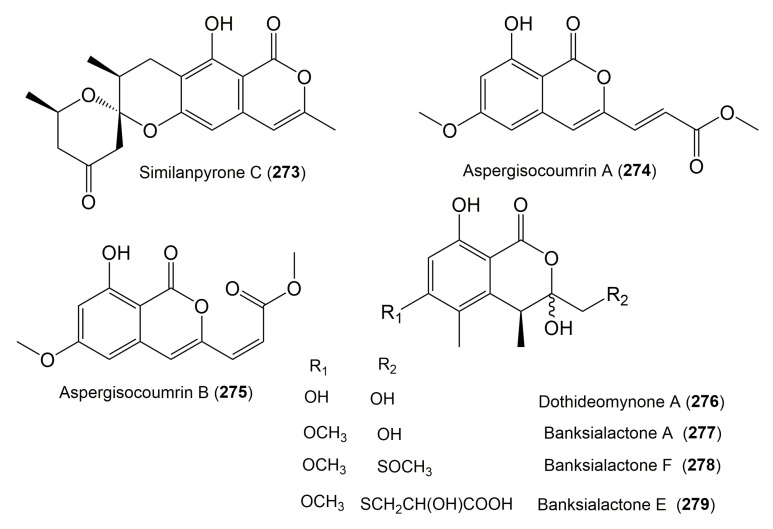
Structures of isocoumarin derivatives **273**–**279**.

**Figure 28:**
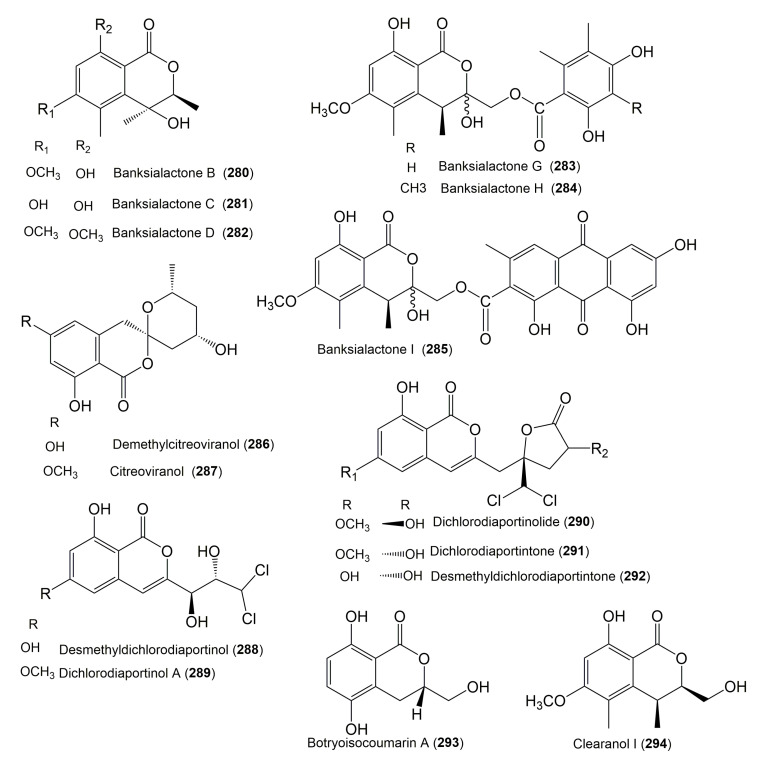
Structures of isocoumarin derivatives **280**–**294**.

**Figure 29:**
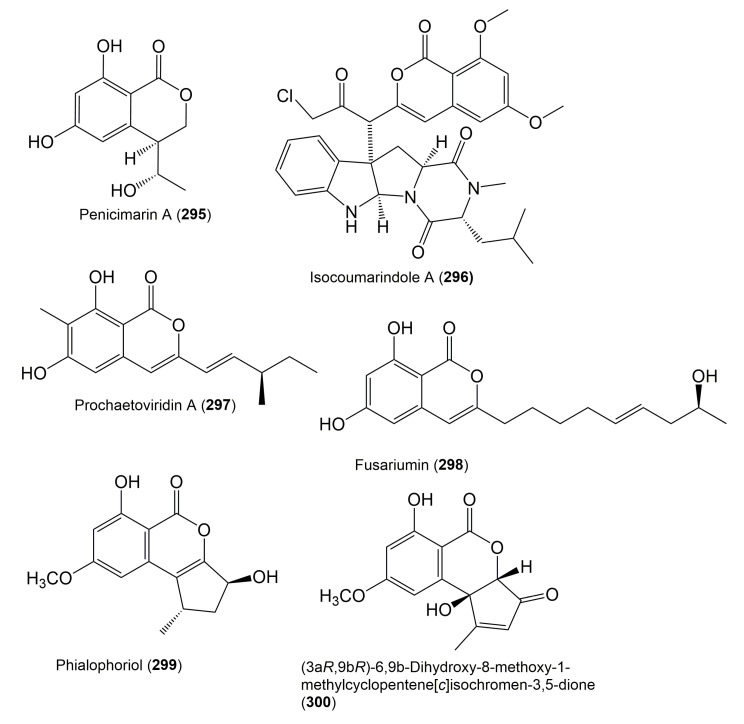
Structures of isocoumarin derivatives **295**–**300**.

**Figure 30:**
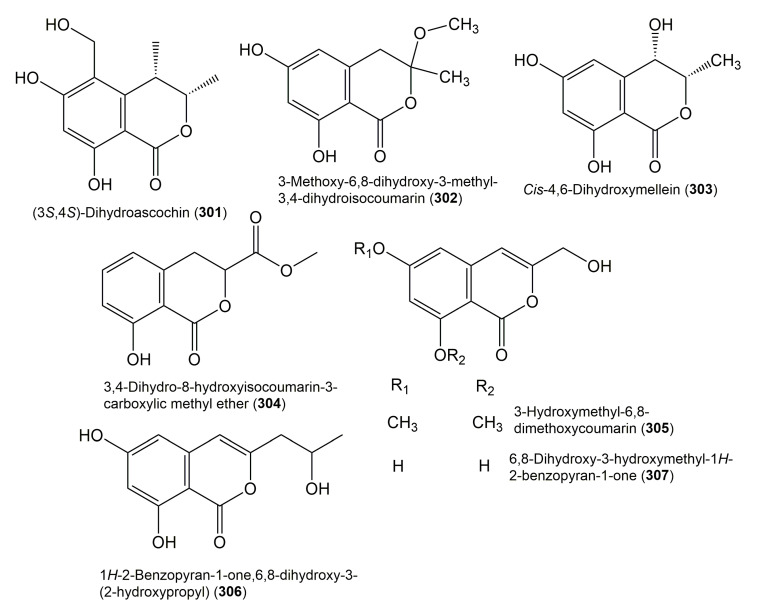
Structures of isocoumarin derivatives **301**–**307**.

**Table 1:** List of fungal isocoumarins (Fungal source, host, and place).

Compound Name	Fungus	Host (Part, Family)	Source, Place	References
Kigelin (**1**) (−)-(3R)-6,7-Dimethoxymellein	*Aspergillus terrus* BDKU 1164	Marine alga	Mubarak village beach, Karachi, Pakistan	[68]
(3R,4R)-6,7-Dimethoxy-4-hydroxymellein (**2**)	*Aspergillus terrus* BDKU 1164	Marine alga	Mubarak village beach, Karachi, Pakistan	[68]
8-Methoxymellein (**3**)	*Penicillium* sp.1 and sp.2	*Alibertia macrophylla* (Leaves, Rubiaceae)	Mogi-Guaçu, São Paulo, Brazil	[18]
	*Botryosphaeria* sp. KcF6	*Kandelia candel* (Fruits, Rhizophoraceae)	Daya Bay, Shenzhen, China	[36]
	*Xylaria cubensis* BCRC 09F 0035	*Litsea akoensis* Hayata (Leaves, Lauraceae)	Kaohsiung, Taiwan	[109]
Cis-4-Acetoxyoxymellein (**4**)	*Ascomycete* 6650	*Meliotus dentatus* (Leaves, Fabaceae)	Baltic Sea, Ahrenshoop, Germany	[50]
8-Deoxy-6-hydroxy-*cis*-4-acetoxyoxymellein (**5**)	*Ascomycete* 6650	*Meliotus dentatus* (Leaves, Fabaceae)	Baltic Sea, Ahrenshoop, Germany	[50]
(3R,4R)-(−)-4-Hydroxymellein (3R,4R)-Cis-4-Hydroxymellein (**6**)	*Aspergillus terrus* (BDKU 1164)	Marine alga	Mubarak village beach, Karachi, Pakistan	[68]
	*Xylaria* sp. PBR-30	*Sandoricum koetjape* (Leaves, Meliaceae)	Prachinburi Province, Thailand	[111]
	*Ascochyta* sp.	*Meliotus dentatus* (Whole plant, Fabaceae)	Shores of the Baltic Sea, near Ahrenshoop, Germany	[117]
	*Nigrospora* sp. PSU-N24	*Garcinia nigrolineata* (Branches, Clusiaceae)	Ton Nga Chang wildlife sanctuary, Songkhla province, Southern Thailand	[110]
	*Neofusicoccum parvum*	*Vitis vinifera* L. (Cankered branchs, Vitaceae)	Catalonia, NE Spain	[114]
	*Emericellopsis minima*	*Hyrtios erecta* (Marine sponge)	Similan islands, Phag Nga Province, Thailand	[92]
	*Apiospora montagnei* Sacc.	*Smallanthus sonchifolius* (Roots, Asteraceae)	Ribeirão Preto city, S. P. State, Brazil	[113]
	*Lachnum palmae*	*Przewalskia tangutica* Maxim. (Leaves, Solanaceae)	Linzhou Country of the Tibet Autonomous Region, China	[51]
Annulohypoxylomarin (**7**)	*Annulohypoxylon truncatum*	*Zizania caduciflora* (Leaves, Poaceae)	Suncheon, South Korea	[45]
5-Hydroxymellein (**8**)	*Penicillium* sp.1 and sp.2	*Alibertia macrophylla* (Leaves, Rubiaceae)	Mogi-Guaçu, São Paulo, Brazil	[18]
	*Lachnum palmae*	*Przewalskia tangutica* Maxim. (Leaves, Solanaceae)	Linzhou Country of the Tibet Autonomous Region, China	[51]
(3R)-6-Methoxy-7-chloromellein (**9**)	*Phoma* sp. 135	*Ectyplasia perox*	Lauro Club Reef, Dominica	[32]
(3R,4R)-Cis-4-Hydroxy-5-methylmellein (**10**)	Unidentified *Ascomycete* 6650	*Meliotus dentatus* (Leaves, Fabaceae)	Baltic Sea, Ahrenshoop, Germany	[43]
(−)-6-Methoxymellein (**11**)	*Lachnum palmae*	*Przewalskia tangutica* Maxim. (Leaves, Solanaceae)	Linzhou Country of the Tibet Autonomous Region, China	[51]
	*Phoma* sp. YE3135	*Aconitum vilmorinianum* (Roots, Ranunculaceae)	Yunnan University, China	[26]
(3R,4S)-4-Hydroxy-6-methoxy-7-chloromellein (**12**)	*Phoma* sp. 135	*Ectyplasia perox*	Lauro Club Reef, Dominica	[32]
Botryospyrone C (**13**)	*Botryosphaeria ramosa* L29	*Myoporum bontioides* (Leaves, Scrophulariaceae)	Leizhou Peninsula, China	[71]
Botryospyrone D (**14**)	*Botryosphaeria ramosa* L29	*Myoporum bontioides* (Leaves, Scrophulariaceae)	Leizhou Peninsula, China	[71]
3R-(+)-5-*O*-[6′-*O*-Acetyl]-α-D-glucopyranosyl-5-hydroxymellein (**15**)	*Xylaria* sp. cfcc 87468	*Pinus tabuliformis* (Leaves, Pinaceae)	China Forestry Culture Collection Center, Beijing, China	[118]
6-(4′-Hydroxy-2′-methyl phenoxy)-(−)-(3R)-mellein (**16**)	*Aspergillus terrus* BDKU 1164	Marine alga	Mubarak village beach, Karachi, Pakistan	[68]
(3R)-7-Hydroxy-5-methylmellein (**17**)	*Phomopsis* sp. 7233	*Laurus azorica* (Leaves, Lauraceae)	Gomera, Spain	[42]
	*Biscogniauxia capnodes*	*Averrhoa carambola* L. (Fruits, Oxalidaceae)	Home garden in Kandy, Central Province, Sri Lanka	[96]
Akolitserin (**18**) (+)-(3R,4S)-5-Carbomethoxy-3-hydroxymellein Methyl (3R,4S)-3,4-Dihydro-4,8-dihydroxy-3-methyl-1-oxo-1H-isochromene-5-carboxylate	*Xylaria cubensis* BCRC 09F 0035	*Litsea akoensis* Hayata (Leaves, Lauraceae)	Kaohsiung, Taiwan	[109]
(−)-(R)-5-(Methoxycarbonyl)mellein (**19**)	*Xylaria cubensis* BCRC 09F 0035	*Litsea akoensis* Hayata (Leaves, Lauraceae)	Kaohsiung, Taiwan	[109]
(3R*,4S*)-6,8-Dihydroxy-3,4,7-trimethylisocoumarin (**20**)	*Penicillium* sp. 091402	*Bruguiera sexangula* (Roots, Rhizophoraceae)	Qinglan Port, Hainan, China	[87]
(3R,4S)-6,8-Dihydroxy-3,4,5,7-tetramethylisochroman (**21**)	*Penicillium* sp. 091402	*Bruguiera sexangula* (Roots, Rhizophoraceae)	Qinglan Port, Hainan, China	[87]
	*Aspergillus versicolor*	*Paris marmorata* Stearn (Rhizomes, Melanthiaceae)	Dali, Yunnan, China	[93]
(3R,4R)-5-Cholro-4,6-dihydroxymellein (**22**)	*Lachnum palmae*	*Przewalskia tangutica* Maxim. (Leaves, Solanaceae)	Linzhou Country of the Tibet Autonomous Region, China	[51]
Palmaerone A (**23**) (R)-5-Bromo-6-hydroxy-8-methoxy-mellein	*Lachnum palmae*	*Przewalskia tangutica* Maxim. (Leaves, Solanaceae)	Linzhou Country of the Tibet Autonomous Region, China	[51]
Palmaerone B (**24**) (R)-7-Bromo-6-hydroxy-8-methoxy-mellein	*Lachnum palmae*	*Przewalskia tangutica* Maxim. (Leaves, Solanaceae)	Linzhou Country of the Tibet Autonomous Region, China	[51]
Palmaerone C (**25**) (R)-7-Bromo-6,8-dimethoxy-mellein	*Lachnum palmae*	*Przewalskia tangutica* Maxim. (Leaves, Solanaceae)	Linzhou Country of the Tibet Autonomous Region, China	[51]
Palmaerone D (**26**) (R)-7-Bromo-6-hydroxy-mellein	*Lachnum palmae*	*Przewalskia tangutica* Maxim. (Leaves, Solanaceae)	Linzhou Country of the Tibet Autonomous Region, China	[51]
Palmaerone E (**27**) (R)-5-Bromo-6,7-dihydroxy-8-methoxy-mellein	*Lachnum palmae*	*Przewalskia tangutica* Maxim. (Leaves, Solanaceae)	Linzhou Country of the Tibet Autonomous Region, China	[51]
Palmaerone F (**28**) (R)-5-Cholro-6-hydroxy-8-methoxy-mellein	*Lachnum palmae*	*Przewalskia tangutica* Maxim. (Leaves, Solanaceae)	Linzhou Country of the Tibet Autonomous Region, China	[51]
Palmaerone G (**29**) (R)-7-Cholro-6-hydroxy-8-methoxy-mellein	*Lachnum palmae*	*Przewalskia tangutica* Maxim. (Leaves, Solanaceae)	Linzhou Country of the Tibet Autonomous Region, China	[51]
(R)-5-Cholro-6-hydroxymellein (**30**)	*Lachnum palmae*	*Przewalskia tangutica* Maxim. (Leaves, Solanaceae)	Linzhou Country of the Tibet Autonomous Region, China	[51]
Palmaerin A (**31**)	*Lachnum palmae*	*Przewalskia tangutica* Maxim. (Leaves, Solanaceae)	Linzhou Country of the Tibet Autonomous Region, China	[51]
Palmaerin B (**32**)	*Lachnum palmae*	*Przewalskia tangutica* Maxim. (Leaves, Solanaceae)	Linzhou Country of the Tibet Autonomous Region, China	[51]
Palmaerin D (**33**)	*Lachnum palmae*	*Przewalskia tangutica* Maxim. (Leaves, Solanaceae)	Linzhou Country of the Tibet Autonomous Region, China	[51]
(3R,4R)-3,4-Dihydro-4,6-dihydroxy-3-methyl-1-oxo-1H-isochromene-5-carboxylic acid (**34**)	Xylaria sp. PA-01	*Piper aduncum* (Leaves, Piperaceae)	Mogi-Guaçu, Săo Paulo, Brazil	[17]
(3R)-Mellein (**35**) 3,4-Dihydro-(3R)-methyl-8-hydroxyisocoumarin	*Centraalbureau voor* Schimmel 120379	*Picea glauca* (Leaves, Pinaceae)	Sussex, New Brunswick, Canada	[119]
	*Nigrospora* sp. PSU-N24	*Garcinia nigrolineata* (Branches, Clusiaceae)	Ton Nga Chang wildlife sanctuary, Songkhla province, Southern Thailand	[119]
	*Nigrospora* sp. LLGLM003	*Moringa oleifera* (Roots, Moringaceae)	Xiamen municipality, Fujian Province, China	[53]
	*Apiospora montagnei* Sacc.	*Smallanthus sonchifolius* (Roots, Asteraceae)	Ribeirão Preto city, S. P. State, Brazil	[113]
	*Lasiodiplodia* sp. ME4-2	*Viscum coloratum* (Flowers, Santalaceae)	Hangzhou City, Zhejiang Province, China	[120]
	*Sarcosomataceae* sp. NO.49-14-2-1	*Everniastrum nepalense* (Taylor) Hale ex Sipman (Lichen, Parmeliaceae)	Panzhihua, Sichuan province, China	[121]
	*Penicillium janczewskii*	*Prumnopitys andina* (Phloem, Podocarpaceae)	Western Andean slopes near Las Trancas, Chillan	[91]
	*Lachnum palmae*	*Przewalskia tangutica* Maxim. (Leaves, Solanaceae)	Linzhou Country of the Tibet Autonomous Region, China	[51]
(R)-7-Hydroxymellein (**36**)	*Penicillium* sp. 05070032-C	*Alibertia macrophylla* (Leaves, Rubiaceae)	Mogi-Guaçu, Săo Paulo, Brazil	[17]
	*Xylaria cubensis* BCRC 09F 0035	*Litsea akoensis* Hayata (Leaves, Lauraceae)	Kaohsiung, Taiwan	[109]
(3R,4R)-4,7-Dihydroxymellein (**37**)	*Penicillium* sp. 05070032-C	*Alibertia macrophylla* (Leaves, Rubiaceae)	Mogi-Guaçu, Săo Paulo, Brazil	[17]
Angelicoin A (**38**)	*Aspergillus versicolor* 0456	*Nicotiana sanderae* (Leaves, Solanaceae)	Shilin, Yunnan Province, China	[122]
	*Aspergillus versicolor*	*Paris marmorata* Stearn (Rhizomes, Melanthiaceae)	Dali, Yunnan, China	[93]
Periplanetin A (**39**)	*Penicillium oxalicum* 0403	*Nicotiana sanderae* (Leaves, Solanaceae)	Shilin, Yunnan Province, China	[44]
(3R)-Methyl-8-hydroxy-6-(hydroxymethyl)-7-methoxydihydroisocoumarin (**40**)	*Aspergillus versicolor*	*Nicotiana tabacum* (Rhizomes, Solanaceae)	Chuxiong, Yunnan, China	[112]
(3R)-Methyl-7,8-dimethoxy-6-(hydroxymethyl) dihydro-isocoumarin (**41**)	*Aspergillus versicolor*	*Nicotiana tabacum* (Rhizomes, Solanaceae)	Chuxiong, Yunnan, China	[112]
(R)-6-Hydroxymellein (**42**)	*Aspergillus versicolor*	*Nicotiana tabacum* (Rhizomes, Solanaceae)	Chuxiong, Yunnan, China	[112]
	*Lachnum palmae*	*Przewalskia tangutica* Maxim. (Leaves, Solanaceae)	Linzhou Country of the Tibet Autonomous Region,	[51]
	*Seltsamia galinsogisoli* sp. nov. SYPF 7336	*Galinsoga parviflora* (Whole plant, Asteraceae)	Huludao, China	[78]
6,8-Dimethoxy-3-methyl-3,4-dihydro-1H-isochromen-1-one (**43**)	*Aspergillus versicolor*	*Nicotiana tabacum* (Rhizomes, Solanaceae)	Chuxiong, Yunnan, China	[112]
Periplanetin B (**44**)	*Aspergillus versicolor*	*Nicotiana tabacum* (Rhizomes, Solanaceae)	Chuxiong, Yunnan, China	[112]
Arundinone A (**45**)	*Microsphaeropsis arundinis*	*Ulmus macrocarpa* (Stems, Ulmaceae)	Dongling Mountain, Beijing, China	[123]
Aspergillspin F (**46**)	*Aspergillus* sp. SCSIO 41501	*Melitodes squamata* (Gorgonian, Plexauridae)	South China Sea, Sanya Hainan Province, China	[70]
(3R)-5-Carbomethoxymellein (**47**) 5-Carbomethyoxy-3,4-dihydro-8-hydroxy-(3R)-methylisocoumarin	*Centra albureau voor* Schimmel cultures 120379	*Picea glauca* (Leaves, Pinaceae)	Sussex, New Brunswick, Canada	[119]
	*Xylaria* sp. PSU-G12	*Garcinia hombroniana* (Branch, Clusiaceae)	Songkhla province, Thailand	[95]
(3R)-5-Formylmellein (**48**) 3,4-Dihydro-5-formyl-8-hydroxy-(3R)-methylisocoumarin	*Centraalbureau voor* Schimmel 120379	*Picea glauca* (Leaves, Pinaceae)	Sussex, New Brunswick, Canada	[119]
Xylarellein (**49**)	*Xylaria* sp. PSU-G12	*Garcinia hombroniana* (Branch, Clusiaceae)	Songkhla province, Thailand	[95]
(3R)-5-Carboxylmellein (**50**)	*Xylaria* sp. PSU-G12	*Garcinia hombroniana* (Branches, Clusiaceae)	Songkhla province, Thailand	[95]
Gamahorin (**51**)	*Pestalotiopsis heterocornis*	*Phakellia fusca* (Sponge, Axinellidae	Xisha Islands, China	[75]
Versicoumarin B (**52**)	*Aspergillus versicolor*	*Paris marmorata* Stearn (Rhizomes, Melanthiaceae)	Dali, Yunnan, China	[93]
Versicoumarin C (**53**)	*Aspergillus versicolor*	*Paris marmorata* Stearn (Rhizomes, Melanthiaceae)	Dali, Yunnan, China	[93]
S-(−)-6-Hydroxy-8-methoxy-4-(1′-hydroxyethyl)-isocoumarin (**54**)	*Talaromyces Amestolkiae* YX1	*Kandelia obovata* (Leaves, Rhizophoraceae)	Zhanjiang, Guangdong Province, China	[62]
Acetic acid 6,8-dihydroxy-3,5-dimethyl-1-oxo-1H-isochromen-4-ylmethyl ester (**55**)	*Scytalidium* sp. 5681	*Salix* sp. (Leaves, Salicaceae)	Harz Mountains, Lower Saxony, Germany	[27]
6,8-Dihydroxy-4-hydroxymethyl-3,5-dimethyl-isochromen-1-one (**56**)	*Scytalidium* sp. 5681	*Salix* sp. (Leaves, Salicaceae)	Harz Mountains, Lower Saxony, Germany	[27]
Decarboxycitrinone (**57**)	*Scytalidium* sp. 5681	*Salix* sp. (Leaves, Salicaceae)	Harz Mountains, Lower Saxony, Germany	[27]
4-Acetyl-6,8-dihydroxy-5-methyl-2- benzopyran-1-one (**58**)	*Scytalidium* sp. 5681	*Salix* sp. (Leaves, Salicaceae)	Harz Mountains, Lower Saxony, Germany	[27]
6,8-Diacetoxy-3,5-dimethylisocoumarin (**59**)	*Mycelia sterile* 4567	Canadian thistle *Cirsium arvense* (Asteraceae)	Lower Saxony, Germany	[20]
Penicilisorin (**60**)	*Penicillium sclerotiorum* PSUA13	*Garcinia atroviridis* (Leaves, Clusiaceae)	Yala Province, Thailand	[124]
Pestalotiorin (**61**)	*Pestalotiopsis* sp. PSU-ES194	*Enhalus acoroides* (Leaves, Hydrocharitaceae)	Songkla Province, Thailand	[41]
Tabaisocoumarin A (**62**)	*Aspergillus versicolor* 0456	*Nicotiana sanderae* (Leaves, Solanaceae)	Shilin, Yunnan Province, China	[122]
	*Aspergillus oryzae*	*Paris polyphylla* var. *yunnanensis* (Franch.) Hand.-Mazz. (Rhizomes, Liliaceae)	Dali, Yunnan, China	[94]
3-Acetoxyl-8-hydroxyl-isocoumarin (**63**)	*Sarcosomataceae* sp. NO.49-14-2-1	*Everniastrum nepalense* (Taylor) Hale ex Sipman (Lichen, Parmeliaceae)	Panzhihua, Sichuan province, China	[121]
6-Hydroxy-4-(1-hydroxyethyl)-8-methoxy-1H-isochromen-1-one (**64**)	*Talaromyces amestolkiae*	*Kandelia obovata* (Leaves, Rhizophoraceae)	Zhanjiang, Guangdong Province, China	[62]
S-(−)-5,6,8-Trihydroxy-4-(1′-hydroxyethyl)isocoumarin (**65**)	*Penicillium* sp. MWZ14-4	Unidentified sponge GX-WZ-2008001 (Inner fresh tissues)	Weizhou, South China Sea, China	[52]
	*Talaromyces amestolkiae*	*Kandelia obovata* (Leave, Rhizophoraceae	Zhanjiang, Guangdong Province, China	[62]
Sescandelin (**66**)	*Penicillium* sp. MWZ14-4	Unidentified sponge GX-WZ-2008001 (Inner fresh tissue)	Weizhou, South China Sea, China	[52]
	*Talaromyces amestolkiae*	*Kandelia obovata* (Leaves, Rhizophoraceae)	Zhanjiang, Guangdong Province, China	[62]
Terrecoumarin A (**67**)	*Penicillium oxalicum* 0403	*Nicotiana sanderae* (Leaves, Solanaceae)	Shilin, Yunnan Province, China	[44]
Terrecoumarin B (**68**)	*Penicillium oxalicum* 0403	*Nicotiana sanderae* (Leaves, Solanaceae)	Shilin, Yunnan Province, China	[44]
Terrecoumarin C (**69**)	*Penicillium oxalicum* 0403	*Nicotiana sanderae* (Leaves, Solanaceae)	Shilin, Yunnan Province, China	[44]
Pestapyrone D (**70**)	*Pestalotiopsis* sp.	*Photinia frasery* (Leaves, Amygdaloideae)	Nanjing, Jiangsu, China	[22]
Pestapyrone E (**71**)	*Pestalotiopsis* sp.	*Photinia frasery* (Leaves, Amygdaloideae)	Nanjing, Jiangsu, China	[22]
LL-Z 1640-7 (**72**)	*Peyronellaea glomerata* XSB-01-15	*Amphimedon* sp. (Sponge, Niphatidae)	Yongxin Island, Hainan Province, China	[65]
Aspergillspin G (**73**)	*Aspergillus* sp. SCSIO 41501	*Melitodes squamata* (Gorgonian, Plexauridae)	South China Sea, Sanya Hainan Province, China	[70]
Acremonone E (**74**)	*Acremonium* sp. PSU-MA70	*Rhizophora apiculata* (Branches, Rhizophoraceae)	Satun Province, Thailand	[125]
Acremonone F (**75**)	*Acremonium* sp. PSU-MA70	*Rhizophora apiculata* (Branches, Rhizophoraceae)	Satun Province, Thailand	[125]
Acremonone G (**76**)	*Acremonium* sp. PSU-MA70	*Rhizophora apiculata* (Branches, Rhizophoraceae)	Satun Province, Thailand	[125]
	*Myrothecium* sp. OUCMDZ-2784	*Apocynum venetum* (Leaves, Apocynaceae)	Dongying, China	[103]
Acremonone H (**77**)	*Acremonium* sp. PSU-MA70	*Rhizophora apiculata* (Branches, Rhizophoraceae)	Satun Province, Thailand	[125]
Daldiniside B (**78**)	*Daldinia eschscholzii*	Scaevola sericea Vahl (Branches, Goodeniaceae)	Hainan province, China	[54]
Daldiniside C (**79**)	*Daldinia eschscholzii*	Scaevola sericea Vahl (Branches, Goodeniaceae)	Hainan province, China	[54]
de-*O*-Methyldiaporthin (**80**)	*Daldinia eschscholzii*	*Scaevola sericea* Vahl (Branches, Goodeniaceae)	Hainan province, China	[54]
	*Penicillium coffeae* MA-314	*Laguncularia racemose* (Leaves, Combretaceae)	Hainan island, China	[47]
Myrothelactone A (**81**)	*Myrothecium* sp. OUCMDZ-2784	*Apocynum venetum* (Leaves, Apocynaceae)	Dongying, China	[103]
Myrothelactone B (**82**)	*Myrothecium* sp. OUCMDZ-2784	*Apocynum venetum* (Leaves, Apocynaceae)	Dongying, China	[103]
3-Methyl-8-hydroxyisocoumarin (**83**)	*Sarcosomataceae* sp. NO.49-14-2-1	*Everniastrum nepalense* (Taylor) Hale ex Sipman (Lichen, Parmeliaceae)	Panzhihua, Sichuan province, China	[121]
6,8-Dihydroxy-5-methoxy-3-methyl-1H-isochromen-1-one (**84**)	*Talaromyces amestolkiae*	*Kandelia obovata* (Leave, Rhizophoraceae)	Zhanjiang, Guangdong Province, China	[62]
Myrothelactone C (**85**)	*Myrothecium* sp. OUCMDZ-2784	*Apocynum venetum* (Leaves, Apocynaceae)	Dongying, China	[103]
Myrothelactone D (**86**)	*Myrothecium* sp. OUCMDZ-2784	*Apocynum venetum* (Leaves, Apocynaceae)	Dongying, China	[103]
Tubakialactone B (**87**) 8-Hydroxyl-3,4-bis(hydroxymethyl)-6-methoxy-4-methyl-1H-2-benzopyran-1-one	*Tubakia* sp. ECN-111	*Houttuynia cordata* Thunb (Leaves, Saururaceae)	Chikusa-ku Nagoya city, Japan	[115]
	*Myrothecium* sp. OUCMDZ-2784	*Apocynum venetum* (Leaves, Apocynaceae)	Dongying, China	[103]
Saccharonol A (**88**) 6,8-Dihydroxy-3-methylisocoumarin	*Aspergillus similanensis* sp. nov. KUFA 0013	*Rhabdermia* sp. (Sponge, Rhabderemiidae)	Phang Nga Province, Thailand	[48]
	*Botryosphaeria* sp. KcF6	*Kandelia candel* (Fruits, Rhizophoraceae)	Daya Bay, Shenzhen, China	[36]
	*Aspergillus versicolor* KJ801852	*Paris polyphylla* var. *yunnanensis* (Rhizomes, Melanthiaceae)	Dali, Yunnan, China	[126]
	*Myrothecium* sp. OUCMDZ-2784	*Apocynum venetum* (Leaves, Apocynaceae)	Dongying, China	[103]
	*Penicillium coffeae* MA-314	*Laguncularia racemose* (Leaves, Combretaceae)	Hainan island, China	[47]
Similanpyrone B (**89**) 6,8-Dihydroxy-3,7-dimethylisocoumarin	*Aspergillus similanensis* sp. nov. KUFA 0013	*Rhabdermia* sp. (Sponge, Rhabderemiidae)	Phang Nga Province, Thailand	[48]
	*Pestalotiopsis* sp. HQD-6	*Rhizophora mucronata* (Leaves, Rhizophoraceae)	Hainan Island, China	[126]
Reticulol (**90**)	*Aspergillus similanensis* sp. nov. KUFA 0013	*Rhabdermia* sp. (Sponge, Rhabderemiidae)	Phang Nga Province, Thailand	[48]
	*Biscogniauxia capnodes*	*Averrhoa carambola* L. (Fruits, Oxalidaceae)	Kandy, Central Province, Sri Lanka	[96]
6-Hydroxy-4-hydroxymethyl-8-methoxy-3-methylisocoumarin (**91**)	Endophytic fungus (No. GX4-1B)	*Bruguiera gymnoihiza* (L.) Savigny (Branch, Rhizophoraceae)	South China Sea in Guangxi province, China	[127]
6-Hydroxy-8-methoxy-3,4-dimethylisocoumarin (**92**)	*Talaromyces amestolkiae*	*Kandelia obovata* (Leave, Rhizophoraceae)	Zhanjiang, Guangdong Province, China	[62]
3,4-Dimethyl-6,8-dihydroxyisocoumarin (**93**)	*Talaromyces amestolkiae*	*Kandelia obovata* (Leaves, Rhizophoraceae)	Zhanjiang, Guangdong Province, China	[62]
	*Nectria pseudotrichia* 120-1NP	*Gliricidia sepium* (Stems, Fabaceae)	Wanagama forest of Universitas, Yogyakarta, Indonesia	[128]
6-Hydroxy-4-hydroxymethyl-8-methoxy-3-methyl-isocoumarin (**94**)	*Talaromyces amestolkiae*	*Kandelia obovata* (Leaves, Rhizophoraceae)	Zhanjiang, Guangdong Province, China	[62]
Sescandelin B (**95**)	*Talaromyces amestolkiae*	*Kandelia obovata* (Leaves, Rhizophoraceae)	Zhanjiang, Guangdong Province, China	[62]
	*Myrothecium* sp. OUCMDZ-2784	*Apocynum venetum* (Leaves, Apocynaceae)	Dongying, China	[103]
6-Hydroxy-3-hydroxymethyl-8-methoxyisocoumarin (**96**)	*Penicillium oxalicum* 0403	*Nicotiana sanderae* (Leaves, Solanaceae)	Shilin, Yunnan Province, China	[44]
4,6-Dihydroxy-3,9-dehydromellein (**97**)	*Penicillium oxalicum* 0403	*Nicotiana sanderae* (Leaves, Solanaceae)	Shilin, Yunnan Province, China	[44]
	Aspergillus *versicolor* KJ801852	*Paris polyphylla* var. yunnanensis (Rhizomes, Melanthiaceae)	Dali, Yunnan, China	[126]
Banksiamarin A (**98**)	*Aspergillus banksianus* sp. nov	*Banksia integrifolia* (Leaves, Proteaceae)	Collaroy, New South Wales, Australia	[30]
Banksiamarin B (**99**)	*Aspergillus banksianus* sp. nov	*Banksia integrifolia* (Leaves, Proteaceae)	Collaroy, New South Wales, Australia	[30]
6,8-Dihydroxyisocoumarin-3-carboxylic acid (**100**)	*Bionectria* sp.	*Raphia taedigera* (Seeds, Arecaceae)	Haut Plateaux region, Cameroon	[82]
	*Nectria pseudotrichia* 120-1NP	*Gliricidia sepium* (Stem, Fabaceae)	Wanagama forest of Universitas, Yogyakarta, Indonesia	[128]
	*Aspergillus* sp. HN15-5D	*Acanthus ilicifolius* (Leaves, Acanthaceae)	Dongzhaigang Mangrove National Nature Reserve, Hainan Island, China.	[73]
Nectriapyrone A (**101**)	*Nectria pseudotrichia* 120-1NP	*Gliricidia sepium* (Stems, Fabaceae)	Wanagama forest of Universitas, Yogyakarta, Indonesia	[128]
Nectriapyrone B (**102**)	*Nectria pseudotrichia* 120-1NP	*Gliricidia sepium* (Stems, Fabaceae)	Wanagama forest of Universitas, Yogyakarta, Indonesia	[128]
6-*O*-Methylreticulol (**103**) 8-Hydroxy-6,7-dimethoxy-3-methylisocoumarin	*Xylariaceae* sp. QGS 01	*Quercus gilva* Blume (Stems, Fagaceae)	EhimeUniversity Garden, Ehime Prefecture, Japan	[102]
	*Biscogniauxia capnodes*	*Averrhoa carambola* L. (Fruits, Oxalidaceae)	Home garden in Kandy, Central Province, Sri Lanka	[96]
7-Hydroxy-3,5-dimethylisochromen-1-one (**104**)	*Phoma* sp. YE3135	*Aconitum vilmorinianum* (Roots, Ranunculaceae)	Yunnan University, China	[26]
6,8-Dihydroxy-3-hydroxymethylisocoumarin (**105**)	*Aspergillus versicolor* KJ801852	*Paris polyphylla* var. yunnanensis (Rhizomes, Melanthiaceae)	Dali, Yunnan, China	[126]
Botryospyrone A (**106**)	*Botryosphaeria ramosa* L29	*Myoporum bontioides* (Leaves, Scrophulariaceae)	Leizhou Peninsula, China	[71]
Botryospyrone B (**107**)	*Botryosphaeria ramosa* L29	*Myoporum bontioides* (Leaves, Scrophulariaceae)	Leizhou Peninsula, China	[71]
Decarboxyhydroxycitrinone (**108**)	*Arthrinium sacchari*	Unidentified sponge	The coast of Atami-shi, ShizuokaPrefecture, Japan	[90]
Tubakialactone A (**109**) 8-Hydroxyl-3- hydroxymethyl-6-methoxy-4-methyl-1H-2-benzopyran-1-one	*Tubakia* sp. ECN-111 (Melanconidaceae)	*Houttuynia cordata* Thunb (Leaves, Saururaceae)	Chikusa-ku Nagoya city, Japan	[115]
6,8-Dihydroxy-7-methyl-1-oxo-1H-isochromene-3-carboxylic acid (**110**)	*Pestalotiopsis coffeae*	*Caryota mitis* (Palm, Arecaceae)	Hainan Province, China	[129]
Oryzaein A (**111**)	*Aspergillus oryzae*	*Paris polyphylla* var. yunnanensis (Franch.) Hand-Mazz. (Rhizomes, Liliaceae)	Dali, Yunnan, China	[94]
Oryzaein B (**112**)	*Aspergillus oryzae*	*Paris polyphylla* var. yunnanensis (Franch.) Hand.-Mazz. (Rhizomes, Liliaceae)	Dali, Yunnan, China	[94]
Caudacoumarin C (**113**)	*Aspergillus oryzae*	*Paris polyphylla* var. yunnanensis (Franch.) Hand.-Mazz. (Rhizomes, Liliaceae)	Dali, Yunnan, China	[94]
4,5,7-Trihydroxy-3-methoxy-3,6-dimethylisochroman-1-one (**114**)	*Aspergillus* sp. 16-5B	*Sonneratia apetala* (Leaves, Lythraceae)	Dongzhaigang Mangrove National Nature Reserve in Hainan Island, China	[38]
5,7-Dihydroxy-3-methoxy-3,6-dimethylisochromane-1,4-dione (**115**)	*Aspergillus* sp. 16-5B	*Sonneratia apetala* (Leaves, Lythraceae)	Dongzhaigang Mangrove National Nature Reserve in Hainan Island, China	[38]
3,4-Dihydro-3,6,8-trihydroxy-3,5-dimethylisocoumarin (**116**)	*Mycelia sterile* 4567	Canadian thistle *Cirsium arvense* (Asteraceae)	Lower Saxony, Germany	[20]
Tenuissimasatin (**117**)	*Alternaria tenuissima*	*Erythrophleum fordii* (Barks, Fabaceae)	Nanning, Guangxi Province, China	[130]
Penicoffrazin B (**118**)	*Penicillium coffeae* MA-314	*Laguncularia racemose* (Leaves, Combretaceae)	Hainan island, China	[47]
Penicoffrazin C (**119**)	*Penicillium coffeae* MA-314	*Laguncularia racemose* (Leaves, Combretaceae)	Hainan island, China	[47]
6,8-Dihydroxy-3-methoxy-3,7-dimethylisochroman-1-one (**120**)	*Pestalotiopsis coffeae*	*Caryota mitis* (Palm, Arecaceae)	Hainan Province, China	[129]
Acremonone B (**121**)	*Acremonium* sp. PSU-MA70	*Rhizophora apiculata* (Branches, Rhizophoraceae)	Satun Province, Thailand	[125]
Acremonone C (**122**)	*Acremonium* sp. PSU-MA70	*Rhizophora apiculata* (Branches, Rhizophoraceae)	Satun Province, Thailand	[125]
Acremonone D (**123**)	*Acremonium* sp. PSU-MA70	*Rhizophora apiculata* (Branches, Rhizophoraceae)	Satun Province, Thailand	[125]
4-Acetyl-3,4-dihydro-6,8,-dihydroxy-3-methoxy-5-methylisocoumarin (**124**)	*Mycelia sterile* 4567	Canadian thistle *Cirsium arvense* (Asteraceae)	Lower Saxony, Germany	[20]
4-Acetyl-3,4-dihydro-6,8-dihydroxy-5-methylisocoumarin (**125**)	*Mycelia sterile* 4567	Canadian thistle *Cirsium arvense* (Asteraceae)	Lower Saxony, Germany	[20]
Phomolactone A (**126**)	*Phomopsis* sp. 7233	*Laurus azorica* (Leaves, Lauraceae)	Gomera, Spain	[42]
	*Aspergillus versicolor*	*Paris marmorata* Stearn (Rhizomes, Melanthiaceae)	Dali, Yunnan, China	[93]
Phomolactone B (**127**)	*Phomopsis* sp. 7233	*Laurus azorica* (Leaves, Lauraceae)	Gomera, Spain	[42]
	*Aspergillus versicolor*	*Paris marmorata* Stearn (Rhizomes, Melanthiaceae)	Dali, Yunnan, China	[93]
Phomolactone C (**128**)	*Phomopsis* sp. 7233	*Laurus azorica* (Leaves, Lauraceae)	Gomera, Spain	[42]
(3R)-3-hydroxymethyl-8-hydroxyl-3,4-dihydroisocoumarin (**129**)	*Sarcosomataceae* sp. NO.49-14-2-1	*Everniastrum nepalense* (Taylor) Hale ex Sipman (Lichen, Parmeliaceae)	Panzhihua, Sichuan province, China	[121]
8-Methylmellein (**130**)	*Sarcosomataceae* sp. NO.49-14-2-1	*Everniastrum nepalense* (Taylor) Hale ex Sipman (Lichen, Parmeliaceae)	Panzhihua, Sichuan province, China	[121]
	*Pestalotiopsis* sp. HHL101	*Rhizophora stylosa* (Branches, Rhizophoraceae)	Dong Zhai Gang-Mangrove Garden, Hainan Island, China	[72]
Trans-4-hydroxymellein (**131**)	*Penicillium* sp.1 and sp.2	*Alibertia macrophylla* (Leaves, Rubiaceae)	Mogi-Guaçu, São Paulo, Brazil	[18]
	*Botryosphaeria* sp. KcF6	*Kandelia candel* (Fruits, Rhizophoraceae),	Daya Bay, Shenzhen, China	[36]
	*Sarcosomataceae* sp. NO.49-14-2-1	*Everniastrum nepalense* (Taylor) Hale ex Sipman (Lichen, Parmeliaceae)	Panzhihua, Sichuan province, China	[121]
	*Lachnum palmae*	*Przewalskia tangutica* Maxim. (Leaves, Solanaceae)	Linzhou Country of the Tibet Autonomous Region, China	[51]
3,5-Dimethyl-8-hydroxy-7-methoxy-3,4-dihydroisocoumarin (**132**)	*Cytospora eucalypticola* SS8	*Eucalyptus perriniana* (Bark, Myrtaceae)	Royal Botanic Gardens, Kew, United Kingdom	[55]
3,5-dimethyl-8-methoxy-3,4-dihydroisocoumarin (**133**)	*Cytospora eucalypticola* SS8	*Eucalyptus perriniana* (Barks, Myrtaceae)	Royal Botanic Gardens, Kew, United Kingdom	[55]
(3R)-5-Methylmellein (**134**) 3,4-Dihydro-(3R),5-dimethyl-8-hydroxyisocoumarin	*Cytospora eucalypticola* SS8	*Eucalyptus perriniana* (Barks, Myrtaceae)	Royal Botanic Gardens, Kew, United Kingdom	[55]
	*Centraalbureau voor* Schimmel cultures (120379)	*Picea glauca* (Leaves, Pinaceae)	Sussex, New Brunswick, Canada	[119]
	*Xylaria* sp. PSU-G12	*Garcinia hombroniana* (Branchs, Clusiaceae)	Songkhla province, Thailand	[95]
	*Xylaria cubensis* (Xylariaceae) BCRC 09F 0035	*Litsea akoensis* Hayata (Leaves, Lauraceae)	Kaohsiung, Taiwan	[109]
	*Biscogniauxia capnodes*	*Averrhoa carambola* L. (Fruits, Oxalidaceae)	Home garden in Kandy, Central Province, Sri Lanka	[96]
5-Hydroxymethylmellein (**135**) 8-Hydroxy-5-hydroxymethyl-3-methyl-3,4-dihydroisocoumarin	*Cytospora eucalypticola* SS8	*Eucalyptus perriniana* (Barks, Myrtaceae)	Royal Botanic Gardens, Kew, United Kingdom	[55]
4,8-Dihydroxy-3,5-dimethyl-3,4-dihydroisocoumarin (**136**)	*Cytospora eucalypticola* SS8	*Eucalyptus perriniana* (Barks, Myrtaceae)	Royal Botanic Gardens, Kew, United Kingdom	[55]
Periplanetin D (**137**)	*Aspergillus versicolor*	*Paris marmorata* Stearn (Rhizomes, Melanthiaceae)	Dali, Yunnan, People’sRepublic of China,	[93]
	*Penicillium oxalicum* 0403	*Nicotiana sanderae* (Leaves, Solanaceae)	Shilin, Yunnan Province, China	[44]
	*Pestalotiopsis coffeae*	*Caryota mitis* (Palm, Arecaceae)	Hainan Province, China	[129]
Pestalactone C (**138**)	*Pestalotiopsis* sp.	*Photinia frasery* (Leaves, Amygdaloideae)	Nanjing, Jiangsu, China	[22]
(4S) (+)-Ascochin (**139**)	*Ascochyta* sp.	*Meliotus dentatus* (Whole plant, Fabaceae)	Shores of the Baltic Sea, near Ahrenshoop, Germany	[117]
(4S)-Thielavic acid (**140**)	*Thielavia* sp. ECN-115	*Crassula ovata* (Stems, Crassulaceae)	Chikusa-ku Nagoya city, Japan	[115]
Phomasatin (**141**)	*Phoma* sp. YN02-P-3	*Sumbaviopsis albicans* J. J. Smith (Leaves, Euphorbiaceae)	Yunnan, China	[84]
3,4-Dihydro-6-methoxy-8-hydroxy-3,4,5-trimethyl-isocoumarin-7-carboxylic acid methyl ester (**142**)	Fungus dz17	Mangrove plant	South China Sea coast, China	[88]
3,4-Dihydro-4,8-dihydroxy-3,5-dimethylisocoumarin (**143**)	Fungus dz17	Mangrove plant	South China Sea coast, China	[88]
3,4-Dihydro-8-hydroxy-3-methylisocoumarin-5-carboxylic acid (**144**)	Fungus dz17	Mangrove plant	South China Sea coast, China	[88]
Pestalotiopisorin B (**145**)	*Pestalotiopsis* sp. HHL101	*Rhizophora stylosa* (Branches, Rhizophoraceae)	Dong Zhai Gang-Mangrove Garden, Hainan Island, China	[72]
Pestaloisocoumarin A (**146**)	*Pestalotiopsis heterocornis*	*Phakellia fusca* (Sponge, Axinellidae	Xisha Islands, China	[75]
Pestaloisocoumarin B (**147**)	*Pestalotiopsis heterocornis*	*Phakellia fusca* (Sponge, Axinellidae	Xisha Islands, China	[75]
Tubakialactone C (**148**) (R)-3,4-Dihydro-4,8-dihydroxy-6-methoxy-4-methyl-3-methylene-1H-2-benzopyran-1-one	*Tubakia* sp. ECN-111 (Melanconidaceae)	*Houttuynia cordata* Thunb (Leaves, Saururaceae)	Chikusa-ku Nagoya city, Japan	[115]
(R)-3,4-dihydro-4-hydroxyl-6,8-dimethoxy-4-methyl-3-methylene-1H-2-benzopyran-1-one (**149**)	*Tubakia* sp. ECN-111	*Houttuynia cordata* Thunb (Leaves, Saururaceae)	Chikusa-ku Nagoya city, Japan	[115]
(6,8-dihydroxy-3-methyl-1-oxo-1H-isochromen-4-yl)methyl-3-methylbutanoate (**150**)	*Talaromyces amestolkiae*	*Kandelia obovata* (Leaves, Rhizophoraceae)	Zhanjiang, Guangdong Province, China	[62]
Penicimarin D (**151**)	*Penicillium* sp. MWZ14-4	Unidentified sponge GX-WZ-2008001 (Inner fresh tissues)	Weizhou, South China Sea, China	[52]
Penicimarin E (**152**)	*Penicillium* sp. MWZ14-4	Unidentified sponge GX-WZ-2008001 (Inner fresh tissues)	Weizhou, South China Sea, China	[52]
Penicimarin F (**153**)	*Penicillium* sp. MWZ14-4	Unidentified sponge GX-WZ-2008001 (Inner fresh tissues)	Weizhou, South China Sea, China	[52]
	*Aspergillus versicolor* KJ801852	*Paris polyphylla* var. yunnanensis (Rhizomes, Melanthiaceae)	Dali, Yunnan, China	[126]
Penicimarin G (**154**)	*Penicillium citrinum* HL-5126	*Bruguiera sexangula* var. rhynchopetala (Roots, Rhizophoraceae)	Hainan Island, P.R. China	[56]
Penicimarin H (**155**)	*Penicillium citrinum* HL-5126	*Bruguiera sexangula* var. rhynchopetala (Roots, Rhizophoraceae)	Hainan Island, China	[56]
Penicimarin I (**156**)	*Penicillium citrinum* HL-5126	*Bruguiera sexangula* var. rhynchopetala (Roots, Rhizophoraceae)	Hainan Island, China	[56]
Penicisimpin A (**157**) 3-(R)-6,8-Dihydroxy-7-methyl-3-pentylisochroman-1-one	*Penicillium simplicissimum* MA-332	*Bruguiera sexangula* var. rhynchopetala (Roots, Rhizophoraceae)	Hainan Island, China	[58]
Penicisimpin B (**158**) 3-(R)-6,8-Dihydroxy-3-pentylisochroman-1-one	*Penicillium simplicissimum* MA-332	*Bruguiera sexangula* var. rhynchopetala (Roots, Rhizophoraceae)	Hainan Island, China	[58]
Penicisimpin C (**159**) 3-(S)-6,8-Dihydroxy-7-methyl-3-(pent-1-enyl)isochroman-1-one	*Penicillium simplicissimum* MA-332	*Bruguiera sexangula* var. rhynchopetala (Roots, Rhizophoraceae)	Hainan Island, China	[58]
Fusarentin 6-methyl ether (**160**)	*Colletotrichum* sp. CRI535-02	*Piper ornatum* (Leaves, Piperaceae)	Tai Rom Yen National Park, Surat Thani Province, Thailand	[80]
Fusarentin 6,7-dimethyl ether (**161**)	*Colletotrichum* sp. CRI535-02	*Piper ornatum* (Leaves, Piperaceae)	Tai Rom Yen National Park, Surat Thani Province, Thailand	[80]
7-Butyl-6,8-dihydroxy-3(R)-pent-11-enylisochroman-1-one (**162**)	*Geotrichum* sp.	*Crassocephalum crepidioides* S. Moore (Stems, Asteraceae)	Songkhla Province, Southern Thailand	[74]
7-But-15-enyl-6,8-dihydroxy-3(R)-pent-11-enylisochroman-1-one (**163**)	*Geotrichum* sp.	*Crassocephalum crepidioides* S. Moore (Stems, Asteraceae)	Songkhla Province, Southern Thailand	[74]
7-Butyl-6,8-dihydroxy-3(R)-pentylisochroman-1-one (**164**)	*Geotrichum* sp.	*Crassocephalum crepidioides* S. Moore (Stems, Asteraceae)	Songkhla Province, Southern Thailand	[74]
Monocerin (**165**)	*Microdochium bolleyi* 8880	*Fagonia cretica* (Leaves, Zygophyllaceae)	Gomera, Spain.	[49]
	*Exserohilum rostratum* EU571210	*Stemona* sp. (Leaves and roots, Stemonaceae)	Amphur Bangban, Ayutthaya Province, Thailand	[46]
	*Colletotrichum* sp. CRI535-02	*Piper ornatum* (Leaves, Piperaceae)	Tai Rom Yen National Park, Surat Thani Province, Thailand	[80]
	*Botryosphaeria* sp. KcF6	*Kandelia candel* (Fruits, Rhizophoraceae)	Daya Bay, Shenzhen, China	[36]
	*Exserohilum rostratum* ER1.1	*Bauhinia guianensis* (Fabaceae)	Embrapa Amazônia Oriental Belém, Brazil	[59]
	*Leptosphaena maculans*	*Osmanthus fragrans* (Leaves, Oleaceae)	China	[104]
7-*O*-Demethylmonocerin (**166**)	*Colletotrichum* sp. CRI535-02	*Piper ornatum* (Leaves, Piperaceae)	Tai Rom Yen National Park, Surat Thani Province, Thailand	[80]
	*Setosphaeria* sp. SCSIO41009	*Callyspongia* sp. (Sponge, Callyspongiidae)	Xuwen, Guangdong Province, China	[64]
(12R)-Hydroxymonocerin (**167**)	*Microdochium bolleyi* 8880	*Fagonia cretica* (Leaves, Zygophyllaceae)	Gomera, Spain	[49]
	*Exserohilum* sp. KJ156361	*Acer truncatum* (Leaves, Sapindaceae)	Dongling Mountain, Beijing, China.	[60]
	*Setosphaeria* sp. SCSIO41009	*Callyspongia* sp. (Sponge, Callyspongiidae)	Guangdong Province, China	[64]
	*Leptosphaena maculans*	*Osmanthus fragrans* (Leaves, Oleaceae)	China	[104]
(11R)-Hydroxymonocerin (**168**)	*Exserohilum rostratum* EU571210	*Stemona* sp. (Leaves and roots, Stemonaceae)	Amphur Bangban, Ayutthaya Province, Thailand	[46]
	*Setosphaeria* sp. SCSIO41009	*Callyspongia* sp. (Sponge, Callyspongiidae)	Guangdong Province, China	[64]
(12S)-Hydroxymonocerin (**169**)	Microdochium bolleyi 8880	*Fagonia cretica* (Leaves, Zygophyllaceae)	Gomera, Spain.	[49]
Exserolide D (**170**)	*Exserohilum* sp. KJ156361	*Acer truncatum* (Leaves, Sapindaceae)	Dongling Mountain, Beijing, China.	[60]
	*Aspergillus oryzae*	*Paris polyphylla* var. yunnanensis (Franch.) Hand.-Mazz. (Rhizomes, Liliaceae)	Dali, Yunnan, China	[94]
Exserolide E (**171**)	*Exserohilum* sp. KJ156361	*Acer truncatum* (Leaves, Sapindaceae)	Dongling Mountain, Beijing, China.	[60]
	*Setosphaeria* sp. SCSIO41009	*Callyspongia* sp. (Sponge, Callyspongiidae)	Guangdong Province, China	[64]
Exserolide I (**172**)	*Setosphaeria* sp. SCSIO41009	*Callyspongia* sp. (Sponge, Callyspongiidae)	Guangdong Province, China	[64]
Exserolide J (**173**)	*Setosphaeria* sp. SCSIO41009	*Callyspongia* sp. (Sponge, Callyspongiidae)	Guangdong Province, China	[64]
Maculansline D (**174**) Isomer of (12R)-12-hydroxymonocerin	*Leptosphaena maculans*	*Osmanthus fragrans* (Leaves, Oleaceae)	China	[104]
Exserolide A (**175**)	*Exserohilum* sp. KJ156361	*Acer truncatum* (Leaves, Sapindaceae)	Dongling Mountain, Beijing, China.	[60]
Exserolide B (**176**)	*Exserohilum* sp. KJ156361	*Acer truncatum* (Leaves, Sapindaceae)	Dongling Mountain, Beijing, China.	[60]
	*Setosphaeria* sp. SCSIO41009	*Callyspongia* sp. (Sponge, Callyspongiidae)	Guangdong Province, China	[64]
Exserolide C (**177**)	*Exserohilum* sp. KJ156361	*Acer truncatum* (Leaves, Sapindaceae)	Dongling Mountain, Beijing, China	[60]
	*Setosphaeria* sp. SCSIO41009	*Callyspongia* sp. (Sponge, Callyspongiidae)	Guangdong Province, China	[64]
Exserolide K (**178**)	*Setosphaeria* sp. SCSIO41009	*Callyspongia* sp. (Sponge, Callyspongiidae)	Guangdong Province, China	[64]
Pestalactone A (**179**)	*Pestalotiopsis* sp.	*Photinia frasery* (Leaves, Amygdaloideae)	Nanjing, Jiangsu, China	[22]
Pestalactone B (**180**)	*Pestalotiopsis* sp.	*Photinia frasery* (Leaves, Amygdaloideae)	Nanjing, Jiangsu, China	[22]
8-Dihydroramulosin (**181**)	*Nigrospora* sp. PSU-N24	*Garcinia nigrolineata* (Branches, Clusiaceae)	Ton Nga Chang wildlife sanctuary, Songkhla province, Southern Thailand	[110]
	*Nigrospora* sp. LLGLM003	*Moringa oleifera* (Roots, Moringaceae)	Xiamen municipality, Fujian Province, China	[53]
6β-Hydroxy-8-dihydroramulosin (**182**)	*Nigrospora* sp. PSU-N24	*Garcinia nigrolineata* (Branches, Clusiaceae)	Ton Nga Chang wildlife sanctuary, Songkhla province, Southern Thailand	[110]
(−) Ramulosin (**183**)	*Talaromyces* sp. JQ769262	*Cedrus deodara* (Twigs, Pinaceae)	Lolab Valley in the Western Himalayas, Kashmir, India	[83]
(3S,4aR,7S)-7,8-Dihydroxy-3-methyl-3,4,10,5,6,7-hexahydro-1H-isochromen-1-one (**184**)	*Talaromyces* sp. JQ769262	*Cedrus deodara* (Twigs, Pinaceae)	Lolab Valley in the Western Himalayas, Kashmir, India	[83]
6-Hydroxyramulosin (**185**)	*Nigrospora* sp. PSU-N24	*Garcinia nigrolineata* (Branches, Clusiaceae)	Ton Nga Chang wildlife sanctuary, Songkhla province, Southern Thailand	[110]
Peniciisocoumarin A (**186**)	*Penicillium* sp. TGM112	*Bruguiera sexangula* var. rhynchopetala (Leaves, Rhizophoraceae)	South China Sea, China	[67]
Peniciisocoumarin B (**187**)	*Penicillium* sp. TGM112	*Bruguiera sexangula* var. rhynchopetala (Leaves, Rhizophoraceae)	South China Sea, China	[67]
Alternariol (**188**)	*Alternaria* sp. II2L4	*Polygonum senegalense* Meisn. (Leaves, Polygonaceae)	Alexandria, Egypt	[40]
	*Alternaria tenuissima* SP-07	*Salvia przewalskii* (Roots, Lamiaceae)	Longxi County, Gansu Province, China	[57]
	*Alternaria alternata*	*Camellia sinensis* (Branches, Theaceae)	Nanjing, Jiangsu Province, China	[63]
	*Peyronellaea glomerata* XSB-01-15	*Amphimedon* sp. (Sponge, Niphatidae)	Yongxin Island, Hainan Province, China	[65]
Alternariol-5-*O*-methyl ether (**189**)	Alternaria sp. II2L4	Polygonum *senegalense Meisn*. (Leaves, Polygonaceae)	Alexandria, Egypt	[40]
	*Alternaria tenuissima* SP-07	*Salvia przewalskii* (Roots, Lamiaceae)	Longxi County, Gansu Province, China	[57]
	*Alternaria alternata*	*Camellia sinensis* (Branches, Theaceae)	Nanjing, Jiangsu Province, China	[63]
	*Peyronellaea glomerata* XSB-01-15	*Amphimedon* sp. (Sponge, Niphatidae)	Yongxin Island, Hainan Province, China	[65]
3′-Hydroxyalternariol 5-*O*-methyl ether (**190**)	*Alternaria* sp. II2L4	*Polygonum senegalense* Meisn. (Leaves, Polygonaceae)	Alexandria, Egypt	[40]
Alternariol 5-*O*-sulfate (**191**)	*Alternaria* sp. II2L4	*Polygonum senegalense* Meisn. (Leaves, Polygonaceae)	Alexandria, Egypt	[40]
Alternariol 5-*O*-methyl ether-4′-*O*-sulfate (**192**)	*Alternaria* sp. II2L4	*Polygonum senegalense* Meisn. (Leaves, Polygonaceae)	Alexandria, Egypt	[40]
Altenuene (**193**)	*Alternaria* sp. II2L4	*Polygonum senegalense* Meisn. (Leaves, Polygonaceae)	Alexandria, Egypt	[40]
	*Alternaria tenuissima* SP-07	*Salvia przewalskii* (Roots, Lamiaceae)	Longxi County, Gansu Province, China	[57]
	*Alternaria alternata*	*Camellia sinensis* (Branches, Theaceae)	Nanjing, Jiangsu Province, China	[63]
(−)-(2R,3R,4aR)-Altenuene-2-acetoxy ester (+)-(2S,3S,4aS)-Altenuene-2-acetoxy ester (**194**)	*Alternaria alternata*	*Camellia sinensis* (Branches, Theaceae)	Nanjing, Jiangsu Province, China	[63]
(−)-(2R,3R,4aR)-Altenuene-3-acetoxy ester (+)-(2S,3S,4aS)-Altenuene-3-acetoxy ester (**195**)	*Alternaria alternata*	*Camellia sinensis* (Branches, Theaceae)	Nanjing, Jiangsu Province, China	[63]
5′-Epialtenuene (**196**)	*Alternaria alternata*	*Camellia sinensis* (Branches, Theaceae)	Nanjing, Jiangsu Province, China	[63]
	*Alternaria* sp. II2L4	*Polygonum senegalense* Meisn. (Leaves, Polygonaceae)	Alexandria, Egypt	[40]
Cycloepoxylactone (**197**)	*Phomopsis* sp. 7233	*Laurus azorica* (Leaves, Lauraceae)	Gomera, Spain	[42]
EI-1941-2 (**198**)	*Phomopsis* sp. 7233	*Laurus azorica* (Leaves, Lauraceae)	Gomera, Spain	[42]
Cycloepoxytriol A (**199**)	*Phomopsis* sp. 7233	*Laurus azorica* (Leaves, Lauraceae)	Gomera, Spain	[42]
Cycloepoxytriol B (**200**)	*Phomopsis* sp. 7233	*Laurus azorica* (Leaves, Lauraceae)	Gomera, Spain	[42]
Exserolide F (**201**)	*Exserohilum* sp. KJ156361	*Acer truncatum* (Leaves, Sapindaceae)	Dongling Mountain, Beijing, China	[60]
	*Aspergillus oryzae*	*Paris polyphylla* var. yunnanensis (Franch.) Hand.-Mazz. (Rhizomes, Liliaceae)	Dali, Yunnan, China	[94]
Isocitreoisocoumarinol (**202**)	*Peyronellaea glomerata* XSB-01-15	*Amphimedon* sp. (Sponge, Niphatidae)	Yongxin Island, Hainan Province, China	[65]
(+)-Citreoisocoumarin (**203**)	*Ampelomyces* sp. EU143251.	*Urospermum picroides* (Flowers, Asteraceae)	Alexandria, Egypt	[39]
	*Peyronellaea glomerata* XSB-01-15	*Amphimedon* sp. (Sponge, Niphatidae)	Yongxin Island, Hainan Province, China	[65]
	*Nectria* sp. HN001	*Sonneratia ovata* (Branches, Lythraceae)	South China Sea in Hainan province, China	[33]
	*Phoma* sp. TA07-1	*Dichotella gemmacea* GX-WZ-2008003-4 (Gorgonian, Plexauridae)	Weizhou coral reef, South China Sea, China	[131]
	*Ascomycota* sp. CYSK-4	*Pluchea indica* (Branches, Asteraceae)	Shankou Mangrove Nature Reserve, Guangxi Province, China	[37]
(+)-6-Methylcitreoisocoumarin (**204**)	*Peyronellaea glomerata* XSB-01-15	*Amphimedon* sp. sponge (Niphatidae)	Yongxin Island, Hainan Province, China	[65]
	*Penicillium commune* QQF-3	*Kandelia candel* (Fruits, Rhizophoraceae)	Guangdong Province, China	[31]
Citreoisocoumarinol (**205**)	*Peyronellaea glomerata* XSB-01-15	*Amphimedon* sp. (Sponge, Niphatidae)	Yongxin Island, Hainan Province, China	[65]
	*Nectria* sp. HN001	*Sonneratia ovata* (Branches, Lythraceae)	South China Sea, Hainan province, China	[33]
	*Phoma* sp. (TA07-1)	*Dichotella gemmacea* GX-WZ-2008003-4 (Gorgonian, Plexauridae)	Weizhou coral reef, South China Sea, China	[131]
12-epicitreoisocoumarinol (**206**)	*Nectria* sp. HN001	*Sonneratia ovata* (Branches, Lythraceae)	South China Sea, Hainan province, China	[33]
Mucorisocoumarin A (**207**)	*Peyronellaea glomerata* XSB-01-15	*Amphimedon* sp. (Sponge, Niphatidae)	Yongxin Island, Hainan Province, China	[65]
Mucorisocoumarin B (**208**)	*Peyronellaea glomerata* XSB-01-15	*Amphimedon* sp. (Sponge, Niphatidae)	Yongxin Island, Hainan Province, China	[65]
	*Ascomycota* sp. CYSK-4	*Pluchea indica* (Branches, Asteraceae)	Shankou Mangrove Nature Reserve, Guangxi Province, China	[37]
Peyroisocoumarin A (**209**)	*Peyronellaea glomerata* XSB-01-15	*Amphimedon* sp. (Sponge, Niphatidae)	Yongxin Island, Hainan Province, China	[65]
Peyroisocoumarin B (**210**)	*Peyronellaea glomerata* XSB-01-15	*Amphimedon* sp. (Sponge, Niphatidae)	Yongxin Island, Hainan Province, China	[65]
Peyroisocoumarin C (**211**)	*Peyronellaea glomerata* XSB-01-15	*Amphimedon* sp. (Sponge, Niphatidae)	Yongxin Island, Hainan Province, China	[65]
Aspergillumarin A (**212**)	*Aspergillus* sp.	*Bruguiera gymnorrhiza* (Leaves, Rhizophoraceae)	South China Sea coast, China	[61]
	*Penicillium* sp. (MWZ14-4)	Unidentified sponge GX-WZ-2008001 (Inner fresh tissues)	Weizhou, South China Sea, China	[52]
	*Talaromyces amestolkiae*	*Kandelia obovata* (Leaves, Rhizophoraceae)	Zhanjiang, Guangdong Province, China	[62]
	*Penicillium citrinum* HL-5126	*Bruguiera sexangula* var. rhynchopetala (Roots, Rhizophoraceae)	Hainan Island, China	[56]
	*Penicillium* sp. TGM112	*Bruguiera sexangula* var. rhynchopetala (Leaves, Rhizophoraceae)	South China Sea, China	[67]
Aspergillumarin B (**213**)	*Aspergillus* sp.	*Bruguiera gymnorrhiza* (Leaves, Rhizophoraceae)	South China Sea coast, China	[61]
	*Penicillium* sp. (MWZ14-4)	Unidentified sponge GX-WZ-2008001 (Inner fresh tissues)	Weizhou, South China Sea, China	[52]
	*Talaromyces amestolkiae*	*Kandelia obovata* (Leaves, Rhizophoraceae)	Zhanjiang, Guangdong Province, China	[62]
Penicimarin B (**214**)	*Penicillium* sp. (MWZ14-4)	Unidentified sponge GX-WZ-2008001 (Inner fresh tissues)	Weizhou, South China Sea, China	[52]
	*Talaromyces amestolkiae*	*Kandelia obovata* (Leaves, Rhizophoraceae)	Zhanjiang, Guangdong Province, China	[62]
Penicimarin C (**215**)	*Penicillium* sp. (MWZ14-4)	Unidentified sponge GX-WZ-2008001 (Inner fresh tissues)	Weizhou, South China Sea, China	[52]
	*Talaromyces amestolkiae*	*Kandelia obovata* (Leaves, Rhizophoraceae)	Zhanjiang, Guangdong Province, China	[62]
	*Penicillium* sp. TGM112	*Bruguiera sexangula* var. rhynchopetala (Leaves, Rhizophoraceae)	South China Sea, China	[67]
(R)-3-((R)-4,5-Dihydroxypentyl)-8-hydroxyisochroman-1-one (**216**)	*Talaromyces amestolkiae*	*Kandelia obovata* (Leaves, Rhizophoraceae)	Zhanjiang, Guangdong Province, China	[62]
5,6-Dihydroxy-3-(4-hydroxypentyl)isochroman-1-one (**217**)	*Talaromyces amestolkiae*	*Kandelia obovata* (Leaves, Rhizophoraceae)	Zhanjiang, Guangdong Province, China	[62]
Maculansline C (**218**) 3S, 10S-Dihydroisocoumarin, (Epimer)	*Leptosphaena maculans*	*Osmanthus fragrans* (Leaves, Oleaceae)	China	[104]
Desmethyldiaportinol (**219**)	*Ampelomyces* sp. EU143251.	*Urospermum picroides* (Flowers, Asteraceae)	Alexandria, Egypt	[39]
	*Phoma* sp. (TA07-1)	*Dichotella gemmacea* GX-WZ-2008003-4 (Gorgonian, Plexauridae)	Weizhou coral reef, South China Sea, China	[131]
Dichlorodiaportin (**220**)	*Trichoderma* sp. 09	*Myoporum bontioides* (Roots, Scrophulariaceae)	Leizhou Peninsula, Guangdong Province, China	[66]
	*Trichoderma* sp. 09	*Myoporum bontioides* (Roots, Scrophulariaceae)	Leizhou Peninsula, Guangdong Province, China	[66]
	*Ascomycota* sp. CYSK-4	*Pluchea indica* (Branches, Asteraceae)	Shankou Mangrove Nature Reserve, Guangxi Province, China	[37]
	*Aspergillus* sp. HN15-5D	*Acanthus ilicifolius* (Leaves, Acanthaceae)	Dongzhaigang Mangrove National Nature Reserve, Hainan Island, China	[73]
Desmethyldichlorodiaportin (**221**)	*Ampelomyces* sp. EU143251.	*Urospermum picroides* (Flowers, Asteraceae)	Alexandria, Egypt	[39]
Peniisocoumarin D (**222**)	*Penicillium commune* QQF-3	*Kandelia candel* (Fruit, Rhizophoraceae)	Guangdong Province, China	[31]
Peniisocoumarin E (**223**)	*Penicillium commune* QQF-3	*Kandelia candel* (Fruits, Rhizophoraceae)	Guangdong Province, China	[31]
Peniisocoumarin F (**224**)	*Penicillium commune* QQF-3	*Kandelia candel* (Fruits, Rhizophoraceae)	Guangdong Province, China	[31]
Peniisocoumarin G (**225**)	*Penicillium commune* QQF-3	*Kandelia candel* (Fruits, Rhizophoraceae)	Guangdong Province, China	[31]
	*Penicillium commune* QQF-3	*Kandelia candel* (Fruits, Rhizophoraceae)	Guangdong Province, China	[31]
Peniisocoumarin H (**226**)	*Penicillium commune* QQF-3	*Kandelia candel* (Fruits, Rhizophoraceae)	Guangdong Province, China	[31]
Peniisocoumarin I (**227**)	*Penicillium commune* QQF-3	*Kandelia candel* (Fruits, Rhizophoraceae)	Guangdong Province, China	[31]
Peniisocoumarin J (**228**)	*Penicillium commune* QQF-3	*Kandelia candel* (Fruits, Rhizophoraceae)	Guangdong Province, China	[31]
3-[(R)-3,3-Dichloro-2-hydroxypropyl]-8-hydroxy-6-methoxy-1H-isochromen-1-one (**229**)	*Penicillium commune* QQF-3	*Kandelia candel* (Fruits, Rhizophoraceae)	Guangdong Province, China	[31]
(+)-Diaporthin (**230**)	*Penicillium commune* QQF-3	*Kandelia candel* (Fruits, Rhizophoraceae)	Guangdong Province, China	[31]
Diaportinol (**231**)	*Peyronellaea glomerata* XSB-01-15	*Amphimedon* sp. (Sponge, Niphatidae)	Yongxin Island, Hainan Province, China	[65]
	*Trichoderma* sp. 09	*Myoporum bontioides* (Roots, Scrophulariaceae)	Leizhou Peninsula, Guangdong Province, China	[66]
	*Phoma* sp. (TA07-1)	*Dichotella gemmacea* GX-WZ-2008003-4 (Gorgonian, Plexauridae)	Weizhou coral reef, South China Sea, China	[131]
	*Ascomycota* sp. CYSK-4	*Pluchea indica* (Branches, Asteraceae)	Shankou Mangrove Nature Reserve, Guangxi Province, China	[37]
(+)-(10R)-7-Hydroxy-3-(2-hydroxy-propyl)-5,6-dimethylisochromen-1-one (**232**)	*Alternaria alternata*	*Camellia sinensis* (Branches, Theaceae)	Nanjing, Jiangsu Province, China	[63]
Peyroisocoumarin D (**233**)	*Peyronellaea glomerata* XSB-01-15	*Amphimedon* sp. (Sponge, Niphatidae)	Yongxin Island, Hainan Province, China	[65]
Orthosporin (**234**)	*Peyronellaea glomerata* XSB-01-15	*Amphimedon* sp. (Sponge, Niphatidae)	Yongxin Island, Hainan Province, China	[65]
8-Methyl-11-chlorodiaporthin (**235**)	*Aspergillus* sp. CPCC 400810	*Cetrelia* sp. (Lichen, Parmeliaceae)	Laojun Mount in Yunnan Province, China	[29]
8-Methyl-11,11-dichlorodiaporthin (**236**)	*Aspergillus* sp. CPCC 400810	*Cetrelia* sp. (Lichen, Parmeliaceae)	Laojun Mount in Yunnan Province, China	[29]
8-Hydroxy-6-methoxy-3-(2,3,3-trihydroxypropyl)-1H-isochromen-1-one (**237**)	*Penicillium funiculosum* Fes1711	*Ficus elastica* (Leaves, Moraceae)	Liaocheng University Arboretum, Liaocheng, Shandong, China	[69]
8-Hydroxy-6-methoxy-3-(1,2,3-trihydroxypropyl)-1H-isochromen-1-one (**238**)	*Penicillium funiculosum* Fes1711	*Ficus elastica* (Leaves, Moraceae)	Liaocheng University Arboretum, Liaocheng, Shandong, China	[69]
Aspergisocoumrin C (**239**)	*Aspergillus* sp. HN15-5D	*Acanthus ilicifolius* (Leaves, Acanthaceae)	Dongzhaigang Mangrove National Nature Reserve, Hainan Island, China	[73]
(3R,4R,10R)-Fusarentin 4-hydroxy-6,7-dimethyl ether (**240**)	*Microdochium bolleyi* 8880	*Fagonia cretica* (Leaves, Zygophyllaceae)	Gomera, Spain	[49]
	*Colletotrichum* sp. CRI535-02	*Piper ornatum* (Leaves, Piperaceae)	Tai Rom Yen National Park, Surat Thani Province, Thailand	[80]
Colletomellein A (**241**)	*Colletotrichum aotearoa* BCRC 09F0161	*Bredia oldhamii* Hook. f. (Leaves, Melastomataceae)	Mutan, Pingtung County, Taiwan	[132]
Colletomellein B (**242**)	*Colletotrichum aotearoa* BCRC 09F0161	*Bredia oldhamii* Hook. f. (Leaves, Melastomataceae)	Mutan, Pingtung County, Taiwan	[132]
Peniciisocoumarin D (**243**)	*Penicillium* sp. TGM112	*Bruguiera sexangula* var. rhynchopetala (Leaves, Rhizophoraceae)	South China Sea, China	[67]
Peniciisocoumarin F (**244**)	*Penicillium* sp. TGM112	*Bruguiera sexangula* var. rhynchopetala (Leaves, Rhizophoraceae)	South China Sea, China	[67]
Peniciisocoumarin H (**245**)	*Penicillium* sp. TGM112	*Bruguiera sexangula* var. rhynchopetala (Leaves, Rhizophoraceae)	South China Sea, China	[67]
3,4-Dihydro-8-hydroxy-6-methoxy-(3R)-propylisocoumarin (**246**)	*Centraalbureau voor* Schimmel cultures (120379)	*Picea glauca* (Leaves, Pinaceae)	Sussex, New Brunswick, Canada	[119]
Peniciisocoumarin C (**247**)	*Penicillium* sp. TGM112	*Bruguiera sexangula* var. rhynchopetala (Leaves, Rhizophoraceae)	South China Sea, China	[67]
Peniciisocoumarin E (**248**)	*Penicillium* sp. TGM112	*Bruguiera sexangula* var. rhynchopetala (Leaves, Rhizophoraceae)	South China Sea, China	[67]
Peniciisocoumarin G (**249**)	*Penicillium* sp. TGM112	*Bruguiera sexangula* var. rhynchopetala (Leaves, Rhizophoraceae)	South China Sea, China	[67]
(R)-3-(3-Hydroxypropyl)-8-hydroxy-3,4-dihydroisocoumarin (**250**)	*Penicillium* sp. TGM112	*Bruguiera sexangula* var. rhynchopetala (Leaves, Rhizophoraceae)	South China Sea, China	[67]
Versicoumarin A (**251**)	*Aspergillus versicolor*	*Paris marmorata* Stearn (Rhizomes, Melanthiaceae)	Dali, Yunnan, China	[93]
Versicoumarin D (**252**)	*Aspergillus versicolor*	*Paris marmorata* Steam (Rhizomes, Melanthiaceae)	Dali, Yunnan, China	[89]
Paraphaeosphaerin A (**253**)	*Paraphaeosphaeria quadriseptata*	*Opuntia leptocaulis* (Rhizosphere, Cactaceae)	Tucson, Arizon	[133]
Paraphaeosphaerin B (**254**)	*Paraphaeosphaeria quadriseptata*	*Opuntia leptocaulis* (Rhizosphere, Cactaceae)	Tucson, Arizon, America	[133]
Chaetochiversin A (**255**)	*Chaetomium chiversii*	*Ephedra fasciculata* (Stems, Ephedraceae)	South mountain park, Phoenix, Arizona, America	[133]
Chaetochiversin B (**256**)	*Chaetomium chiversii*	*Ephedra fasciculata* (Stems, Ephedraceae)	South mountain park, Phoenix, Arizona, America	[133]
Paraphaeosphaerin C (**257**)	*Paraphaeosphaeria quadriseptata*	*Opuntia leptocaulis* (Rhizosphere, Cactaceae)	Tucson, Arizon, America	[133]
Peniisocoumarin C (**258**)	*Penicillium commune* QQF-3	*Kandelia candel* (Fruits, Rhizophoraceae)	Guangdong Province, China.	[31]
6,6′-Dinor-bipenicilisorin (**259**)	*Aspergillus versicolor* KU258497	*Eichhornia crassipes* (Leaves, Pontederiaceae)	Mansoura, Egypt	[81]
6,6′,9′-Trinor-bipenicilisorin (**260**)	*Aspergillus versicolor* KU258497	*Eichhornia crassipes* (Leaves, Pontederiaceae)	Mansoura, Egypt	[81]
Asperisocoumarin A (**261**)	*Aspergillus* sp. 085242	*Acanthus ilicifolius* (Roots, Acanthaceae)	Shankou Mangrove National Nature Reserve, Guangxi Province, China	[76]
Asperisocoumarin B (**262**)	*Aspergillus* sp. 085242	*Acanthus ilicifolius* (Roots, Acanthaceae)	Shankou Mangrove National Nature Reserve, Guangxi Province, China	[76]
Asperisocoumarin C (**263**)	*Aspergillus* sp. 085242	*Acanthus ilicifolius* (Roots, Acanthaceae)	Shankou Mangrove National Nature Reserve, Guangxi Province, China	[76]
Asperisocoumarin D (**264**)	*Aspergillus* sp. 085242	*Acanthus ilicifolius* (Roots, Acanthaceae)	Shankou Mangrove National Nature Reserve, Guangxi Province, China	[76]
Asperisocoumarin E (**265**)	*Aspergillus* sp. 085242	*Acanthus ilicifolius* (Roots, Acanthaceae)	Shankou Mangrove National Nature Reserve, Guangxi Province, China	[76]
Asperisocoumarin F (**266**)	*Aspergillus* sp. 085242	*Acanthus ilicifolius* (Roots, Acanthaceae)	Shankou Mangrove National Nature Reserve, Guangxi Province, China	[76]
Peniisocoumarin A (**267**)	*Penicillium commune* QQF-3	*Kandelia candel* (Fruit, Rhizophoraceae)	Guangdong Province, China	[31]
Peniisocoumarin B (**268**)	*Penicillium commune* QQF-3	*Kandelia candel* (Fruits, Rhizophoraceae)	Guangdong Province, China	[31]
Sg17-1-4 (**269**)	*Alternaria tenuis* Sg17-1	Marine alga	Zhoushan Island, Zhejiang Province, China	[85]
AI-77-B (**270**)	*Alternaria tenuis* Sg17-1	Marine alga	Zhoushan Island, Zhejiang Province, China	[85]
AI-77-F (**271**)	*Alternaria tenuis* Sg17-1	Marine alga	Zhoushan Island, Zhejiang Province, China	[85]
Similanpyrone A (**272**) 5-Hydroxy-8-methyl-2H,6H-pyrano [3,4-g]chromen-2,6-dione	*Aspergillus similanensis* sp. nov. KUFA 0013	*Rhabdermia* sp. (Sponge, Rhabderemiidae)	Phang Nga Province, Thailand	[48]
Similanpyrone C (**273**)	*Aspergillus similanensis* KUFA 0013	*Rhabdermia* sp. (Sponge, Rhabderemiidae)	Phang Nga Province, Thailand	[28]
Aspergisocoumrin A (**274**)	*Aspergillus* sp. HN15-5D	*Acanthus ilicifolius* (Leaves, Acanthaceae)	Dongzhaigang Mangrove National Nature Reserve, Hainan Island, China	[73]
Aspergisocoumrin B (**275**)	*Aspergillus* sp. HN15-5D	*Acanthus ilicifolius* (Leaves, Acanthaceae)	Dongzhaigang Mangrove National Nature Reserve, Hainan Island, China.	[73]
Dothideomynone A (**276**)	*Aspergillus banksianus* sp. nov	*Banksia integrifolia* (Leaves, Proteaceae)	Collaroy, New South Wales, Australia	[30]
Banksialactone A (**277**)	*Aspergillus banksianus* sp. nov	*Banksia integrifolia* (Leaves, Proteaceae)	Collaroy, New South Wales, Australia	[30]
Banksialactone F (**278**)	*Aspergillus banksianus* sp. nov	*Banksia integrifolia* (Leaves, Proteaceae)	Collaroy, New South Wales, Australia	[30]
Banksialactone E (**279**)	*Aspergillus banksianus* sp. nov	*Banksia integrifolia* (Leaves, Proteaceae)	Collaroy, New South Wales, Australia	[30]
Banksialactone B (**280**)	*Aspergillus banksianus* sp. nov	*Banksia integrifolia* (Leaves, Proteaceae)	Collaroy, New South Wales, Australia	[30]
Banksialactone C (**281**)	*Aspergillus banksianus* sp. nov	*Banksia integrifolia* (Leaves, Proteaceae)	Collaroy, New South Wales, Australia	[30]
Banksialactone D (**282**)	*Aspergillus banksianus* sp. nov	*Banksia integrifolia* (Leaves, Proteaceae)	Collaroy, New South Wales, Australia	[30]
Banksialactone G (**283**)	*Aspergillus banksianus* sp. nov	*Banksia integrifolia* (Leaves, Proteaceae)	Collaroy, New South Wales, Australia	[30]
Banksialactone H (**284**)	*Aspergillus banksianus* sp. nov	*Banksia integrifolia* (Leaves, Proteaceae)	Collaroy, New South Wales, Australia	[30]
Banksialactone I (**285**)	*Aspergillus banksianus* sp. nov	*Banksia integrifolia* (Leaves, Proteaceae)	Collaroy, New South Wales, Australia	[30]
Demethylcitreoviranol (**286**)	*Peyronellaea glomerata* XSB-01-15	*Amphimedon* sp. (Sponge, Niphatidae)	Yongxin Island, Hainan Province, China	[65]
Citreoviranol (**287**)	*Peyronellaea glomerata* XSB-01-15	*Amphimedon* sp. (Sponge, Niphatidae)	Yongxin Island, Hainan Province, China	[65]
Desmethyldichlorodiaportinol (**288**)	*Ascomycota* sp. CYSK-4	*Pluchea indica* (Branches, Asteraceae)	Shankou Mangrove Nature Reserve, Guangxi Province, China	[37]
Dichlorodiaportinol A (**289**)	*Trichoderma* sp., 09	*Myoporum bontioides* (Roots, Scrophulariaceae)	Leizhou Peninsula, Guangdong Province, China	[79]
Dichlorodiaportinolide (**290**)	*Trichoderma* sp. 09	*Myoporum bontioides* (Roots, Scrophulariaceae)	Leizhou Peninsula, Guangdong Province, China	[66]
Dichlorodiaportintone (**291**)	*Ascomycota* sp. CYSK-4	*Pluchea indica* (Branches, Asteraceae)	Shankou Mangrove Nature Reserve, Guangxi Province, China	[37]
Desmethyldichlorodiaportintone (**292**)	*Ascomycota* sp. CYSK-4	*Pluchea indica* (Branches, Asteraceae)	Shankou Mangrove Nature Reserve, Guangxi Province, China	[37]
Botryoisocoumarin A (**293**) 3S-5,8-dihydroxy-3-hydroxymethyldihydroisocoumarin	*Botryosphaeria* sp. KcF6	*Kandelia candel* (Fruits, Rhizophoraceae)	Daya Bay, Shenzhen, China	[36]
Clearanol I (**294**)	*Aspergillus banksianus* sp. nov	*Banksia integrifolia* (Leaves, Proteaceae)	Collaroy, New South Wales, Australia	[30]
Penicimarin A (**295**)	*Penicillium* sp. MWZ14-4	Unidentified sponge GX-WZ-2008001 (Inner fresh tissues)	Weizhou, South China Sea, China	[52]
Isocoumarindole A (**296**)	*Aspergillus* sp. CPCC400810	*Cetrelia* sp. (Lichen, Parmeliaceae)	Laojun Mount in Yunnan Province, China	[29]
Prochaetoviridin A (**297**)	*Chaetomium globosum* CDW7 (Chaetomiaceae)	*Ginkgo biloba* (Ginkgoaceae)	Jiangsu province, China	[77]
Fusariumin (**298**)	*Fusarium* sp. LN-10	*Melia azedarach* (Leaves, Meliaceae)	Campus of Northwest A&F University, Yangling, Shaanxi province, China,	[86]
	*Aspergillus versicolor* KJ801852	*Paris polyphylla* var. yunnanensis (Rhizomes, Melanthiaceae)	Dali, Yunnan, China	[126]
Phialophoriol (**299**)	*Alternaria alternata*	*Camellia sinensis* (Branches, Theaceae)	Nanjing, Jiangsu Province, China	[63]
(3aR,9bR)-6,9b-Dihydroxy-8-methoxy-1-methylcyclopentene[c]isochromen-3,5-dione (**300**)	*Penicillium* sp.	*Riccardia multifida* (L.) S. Gray (Liverwort, Aneuraceae)	Maoer Mountain, Guangxi Province, China	[116]
(3S,4S)-Dihydroascochin (**301**)	*Phomopsis* sp. 7233	*Laurus azorica* (Leaves, Lauraceae)	Gomera, Spain	[42]
3-Methoxy-6,8-dihydroxy-3-methyl-3,4-dihydroisocoumarin (**302**)	*Penicillium coffeae* MA-314	*Laguncularia racemose* (Leaves, Combretaceae)	Hainan island, China	[47]
Cis-4,6-Dihydroxymellein (**303**)	*Penicillium coffeae* MA-314	*Laguncularia racemose* (Leaves, Combretaceae)	Hainan island, China	[47]
3,4-Dihydro-8-hydroxyisocoumarin-3-carboxylic methyl ether (**304**)	*Seltsamia galinsogisoli* sp. nov. SYPF 7336	*Galinsoga parviflora* (Whole plant, Asteraceae)	Huludao, China	[78]
3-Hydroxymethyl-6,8-dimethoxycoumarin (**305**)	Endophytic fungus No. GX4-1B	*Bruguiera gymnoihiza* (L.) Savigny (Branches, Rhizophoraceae)	South China Sea, Guangxi province, China	[127]
1H-2-Benzopyran-1-one,6,8-dihydroxy-3-(2-hydroxypropyl) (**306**)	*Seltsamia galinsogisoli* sp. nov. SYPF 7336	*Galinsoga parviflora* (Whole plant, Asteraceae)	Huludao, China	[78]
6,8-Dihydroxy-3-hydroxymethyl-1H-2-benzopyran-1-one (**307**)	*Penicillium coffeae* MA-314	*Laguncularia racemose* (Leaves, Combretaceae)	Hainan island, China	[47]

**Table 2:** Biological activities of the most active fungal isocoumarins.

Compound Name	Biological Activity	Assay, Organism, or Cell Line	Biological Results	Positive Control	References	
Kigelin (**1**) (−)-(3R)-6,7-Dimethoxymellein	Antifungal	Agar tube dilution/*Trichophyton longifusus*	45 (% Inhibition)	Miconazole 70 (% Inhibition)	[68]	
	Antifungal	Agar tube dilution/*A. flavus*	20 (% Inhibition)	Ampicillin 20 (% Inhibition)	[68]	
	Antifungal	Agar tube dilution/*Microsporum canis*	50 (% Inhibition)	Miconazole 98.4 (% Inhibition)	[68]	
(3R,4R)-6,7-Dimethoxy-4-hydroxymellein (**2**)	Antifungal	Agar tube dilution/*Trichophyton longifusus*	70 (% Inhibition)	Miconazole 70 (% Inhibition)	[68]	
	Antifungal	Agar tube dilution/*Microsporum canis*	50 (% Inhibition)	Miconazole 98.4 (% Inhibition)	[68]	
	Antifungal	Agar tube dilution/*Fusarium solani*	20 (% Inhibition)	Miconazole 73.2 (% Inhibition)	[68]	
	Antioxidant	XO Inhibition	707 μM (IC_50_)	PG 628 μM (IC_50_) BHA 591 μM (IC_50_)	[68]	
Cis-4-Acetoxyoxymellein (**4**)	Antibacterial	Agar diffusion/*E. coli*	10 mm (GI)	Penicillin 14 mm (GI) Tetracycline 18 mm (GI)	[50]	
	Antibacterial	Agar diffusion/*Bacillus megaterium*	10 mm (GI)	Penicillin 18 mm (GI) Tetracycline 18 mm (GI)	[50]	
	Antifungal	Agar diffusion/*Microbotryum violaceum*	8 mm (GI)	Nystatin 20 mm (IZD) Actidione 50 mm (IZD)	[50]	
	Antifungal	Agar diffusion/*Septoria tritici*	8 mm (IZD)		[50]	
	Algicidal	Agar diffusion/*Chlorella fusca*	7 mm (IZD)	Actidione 35 mm (IZD)	[50]	
8-Deoxy-6-hydroxy-cis-4-acetoxyoxymellein (**5**)	Antibacterial	Agar diffusion/*E*. *coli*	9 mm (GI)	Penicillin 14 mm (GI) Tetracycline 18 mm (GI)	[50]	
	Antibacterial	Agar diffusion/*Bacillus megaterium*	9 mm (GI)	Penicillin 18 mm (GI) Tetracycline 18 mm (GI)	[50]	
	Antifungal	Agar diffusion/*Microbotryum violaceum*	8 mm (GI)	Nystatin 20 mm (IZD) Actidione 50 mm (IZD)	[50]	
	Antifungal	Agar diffusion/*Botrytis cinerea*	10 mm (IZD)	Nystatin 0 mm (IZD) Actidione 0 mm (IZD)	[50]	
	Antifungal	Agar diffusion/*Septoria tritici*	9 mm (IZD)		[50]	
	Algicidal	Agar diffusion/*Chlorella fusca*	8 mm (IZD)	Actidione 35 mm (IZD)	[50]	
(3R,4R)-(−)-4-Hydroxymellein (3R,4R)-Cis-4-Hydroxymellein (**6**)	Antibacterial	Agar diffusion/*Bacillus megaterium*	6 mm (GI)	Penicillin 14 mm (GI) Tetracycline 18 mm (GI)	[43]	
	Antifungal	Agar diffusion/*Microbotryum violaceum*	8 mm (IZD)	Nystatin 20 mm (IZD) Actidione 50 mm (IZD)	[43]	
	Algicidal	Agar diffusion/*Chlorella fusca*	9 mm (IZD)	Actidione 35 mm (IZD)	[43]	
(−)-6-Methoxymellein (**11**)	Antiviral	CPE inhibition/H1N1 virus	20.98 µg/mL (IC_50_)	Arbidol 0.15 µg/mL (IC_50_)	[26]	
Botryospyrone C (**13**)	Antifungal	2-Fold broth dilution method/*F. oxysporum*	223 μM (MIC)	Triadimefon 340 μM (MIC)	[71]	
	Antifungal	2-Fold broth dilution method/*F. graminearum*	223 μM (MIC)	Triadimefon 510.7 μM (MIC)	[71]	
6-(4′-Hydroxy-2′-methyl phenoxy)-(−)-(3R)-mellein (**16**)	Antifungal	Agar tube dilution/*Trichophyton longifusus*	55 (% Inhibition)	Miconazole 70 (% Inhibition)	[68]	
	Antifungal	Agar tube dilution/*Microsporum canis*	70 (% Inhibition)	Miconazole 98.4 (% Inhibition)	[68]	
	Antifungal	Agar tube dilution/*Fusarium solani*	30 (% Inhibition)	Miconazole 73.2 (% Inhibition)	[68]	
	Antioxidant	DPPH	159 μM (IC_50_)	PG 30 159 μM (IC_50_) BHA 44 μM (IC_50_)	[68]	
	Antioxidant	XO Inhibition	243 μM (IC_50_)	PG 628 μM (IC_50_) BHA 591 μM (IC_50_)	[68]	
(3R,4R)-3,4-Dihydro-4,6-dihydroxy-3-methyl-1-oxo-1H-isochromene-5-carboxylic acid (**34**)	Antifungal	Direct Bioautography Overaly/*Cladosporium cladosporioides*	10 µg (Minimum amount required for inhibition of fungi growth on TLC plates)	Nystatin 1µg (Minimum amount required for inhibition of fungi growth on TLC plates)	[17]	
	Antifungal	Direct Bioautography Overlay/*Cladosporium sphaerospermum*	25 µg (Minimum amount required for inhibition of fungi growth on TLC plates)	Nystatin 1 µg (Minimum amount required for inhibition of fungi growth on TLC plates)	[17]	
	Acetylcholinesterase inhibitory	TLC-based AChE inhibition	3 µg (IC)	Galantamine 1µg (IC)	[17]	
(3R)-Mellein (**35**) 3,4-Dihydro-(3R)-methyl-8-hydroxyisocoumarin	Antifungal	Agar diffusion/*Botrytis cinerea*	49.2 µg/mL (EC_50_)	-	[53]	
(R)-7-Hydroxymellein (**36**)	Antifungal	Direct Bioautography Overlay/*Cladosporium cladosporioides*	5 µg (Minimum amount required for inhibition of fungi growth on TLC plates)	Nystatin 1µg (Minimum amount required for inhibition of fungi growth on TLC plates)	[17]	
	Antifungal	Direct Bioautography Overlay/*Cladosporium sphaerospermum*	10 µg (Minimum amount required for inhibition of fungi growth on TLC plates)	Nystatin 1µg (Minimum amount required for inhibition of fungi growth on TLC plates)	[17]	
	Acetylcholinesterase inhibitory	TLC-based AChE inhibition	10 µg (IC)	Galantamine 1µg (IC)	[17]	
(3R,4R)-4,7-Dihydroxymellein (**37**)	Antifungal	Direct Bioautography Overlay/*Cladosporium cladosporioides*	5 µg (Minimum amount required for inhibition of fungi growth on TLC plates)	Nystatin 1 µg (Minimum amount required for inhibition of fungi growth on TLC plates)	[17]	
	Antifungal	Direct Bioautography Overlay/*Cladosporium sphaerospermum*	10 µg (Minimum amount required for inhibition of fungi growth on TLC plates)	Nystatin 1 µg (Minimum amount required for inhibition of fungi growth on TLC plates)	[17]	
	Acetylcholinesterase inhibitory	TLC-based AChE inhibition	10 µg (IC)	Galantamine 1µg (IC)	[17]	
Periplanetin A (**39**)	Antivirus	Spectrophotometer/Anti-TMV	14.6% GI (20 μM)	Ningnanmycin 28.6% GI (20 μM)	[44]	
(3R)-Methyl-8-hydroxy-6-(hydroxymethyl)-7-methoxydihydroisocoumarin (**40**)	Antiviral	Spectrophotometer/Anti-TMV	21.8% GI (20 μM)	Ningnanmycin 32.8% GI (20 μM)	[112]	
(3R)-Methyl-7,8-dimethoxy-6-(hydroxymethyl) dihydroisocoumarin (**41**)	Antiviral	Spectrophotometer/Anti-TMV	18.6% GI (20 μM)	Ningnanmycin 32.8% GI (20 μM)	[112]	
S-(-)-5-Hydroxy-8-methoxy-4-(1′-hydroxyethyl)-isocoumarin (**54**)	α-Glucosidase inhibitory	Chromogenic	537.3 μM (IC_50_)	Acarbose 958.3 μM (IC_50_)	[62]	
S-(-)-5,6,8-Trihydroxy-4-(1′-hydroxyethyl)isocoumarin (**65**)	α-Glucosidase inhibitory	Chromogenic	315.3 μM (IC_50_)	Acarbose 958.3 μM (IC_50_)	[62]	
	Antibacterial	Colorimetric broth microdilution/*S. aureus* (ATCC 27154)	12.5 μM (MIC_50_)	Ciprofloxacin 0.160 μM (MIC_50_)	[52]	
	Antibacterial	Colorimetric broth microdilution/*B. cereus* (ACCC 11077)	6.25 μM (MIC_50_)	Ciprofloxacin 0.625 μM (MIC_50_)	[52]	
	Antibacterial	Colorimetric broth microdilution/*Vibrio parahemolyticus* (ATCC17802)	6.25 μM (MIC_50_)	Ciprofloxacin 0.160 μM (MIC_50_)	[52]	
Sescandelin (**66**)	α-Glucosidase inhibitory	Chromogenic	417.8 μM (IC_50_)	Acarbose 958.3 μM (IC_50_)	[62]	
Terrecoumarin A (**67**)	Antivirus	Spectrophotometer/Anti-TMV	25.4% GI (20 μM)	Ningnanmycin 28.6% GI (20 μM)	[44]	
Terrecoumarin B (**68**)	Antivirus	Spectrophotometer/Anti-TMV	14.5% GI (20 μM)	Ningnanmycin 28.6% GI (20 μM)	[44]	
Terrecoumarin C (**69**)	Antivirus	Spectrophotometer/Anti-TMV	16.3% GI (20 μM)	Ningnanmycin 28.6% GI (20 μM)	[44]	
LL-Z 1640-7 (**72**)	Antioxidant	Luciferase	0.87 mM (IC_50_)	tBHQ 4.29 mM (IC_50_)	[65]	
Acremonone G (**76**)	α-Glucosidase inhibitory	Chromogenic	0.37 mM (IC_50_)	Acarbose 0.47 mM (IC_50_)	[103]	
Myrothelactone A (**81**)	α-Glucosidase inhibitory	Chromogenic	0.32 mM (IC_50_)	Acarbose 0.47 mM (IC_50_)	[103]	
6,8-Dihydroxy-5-methoxy-3-methyl-1H-isochromen-1-one (**84**)	α-Glucosidase inhibitory	Chromogenic	89.4 μM (IC_50_)	Acarbose 958.3 μM (IC_50_)	[62]	
Myrothelactone C (**85**)	α-Glucosidase inhibitory	Chromogenic	0.036 mM (IC_50_)	Acarbose 0.47 mM (IC_50_)	[103]	
Tubakialactone B (**87**) 8-Hydroxyl-3,4-bis(hydroxymethyl)-6-methoxy-4-methyl-1H-2-benzopyran-1-one	α-Glucosidase inhibitory	Chromogenic	0.026 mM (IC_50_)	Acarbose 0.47 mM (IC_50_)	[103]	
6-Hydroxy-8-methoxy-3,4-dimethylisocoumarin (**92**)	α-Glucosidase inhibitory	Chromogenic	585.7 μM (IC_50_)	Acarbose 958.3 μM (IC_50_)	[62]	
3,4-Dimethyl-6,8-dihydroxyisocoumarin (**93**)	α-Glucosidase inhibitory	Chromogenic	36.4 μM (IC_50_)	Acarbose 958.3 μM (IC_50_)	[62]	
6-Hydroxy-4-hydroxymethyl-8-methoxy-3-methyl-isocoumarin (**94**)	α-Glucosidase inhibitory	Chromogenic	302.6 μM (IC_50_)	Acarbose 958.3 μM (IC_50_)	[62]	
Sescandelin B (**95**)	α-Glucosidase inhibitory	Chromogenic	17.2 μM (IC_50_)	Acarbose 958.3 μM (IC_50_)	[62]	
6-Hydroxy-3-hydroxymethyl-8-methoxyisocoumarin (**96**)	Antivirus	Spectrophotometer/Anti-TMV	18.7% GI (20 μM)	Ningnanmycin 28.6% GI (20 μM)	[44]	
4,6-Dihydroxy-3,9-dehydromellein (**97**)	Antivirus	Spectrophotometer/Anti-TMV	13.8% GI (20 μM)	Ningnanmycin 28.6% GI (20 μM)	[44]	
Botryospyrone A (**106**)	Antifungal	2-Fold broth dilution method/*F. oxysporum*	112.6 μM (MIC)	Triadimefon 340 μM (MIC)	[71]	
Botryospyrone B (**107**)	Antifungal	2-Fold broth dilution method/*F. oxysporum*	105.8 μM (MIC)	Triadimefon 340 μM (MIC)	[71]	
	Antifungal	2-Fold broth dilution method/*F. graminearum*	211.7 μM (MIC)	Triadimefon 510.7 μM (MIC)	[71]	
4,5,7-Trihydroxy-3-methoxy-3,6-dimethylisochroman-1-one (**114**)	α-Glucosidase inhibitory	-	IC_50_ 90.4 μM	Acarbose (IC_50_ 553.7 μM)	[38]	
3,5-Dimethyl-8-hydroxy-7-methoxy-3,4-dihydroisocoumarin (**132**)	Antifungal	TLC-autobiography/*Aspergillus niger*	50 µg/mL (MIC)	Nystatin 12.5 µg/mL (MIC)	[55]	
		TLC-autobiography/*Cladosporium herbarum*	50 µg/mL (MIC)	Nystatin 12.5 µg/mL (MIC)	[55]	
	Antibacterial	Colorimetric broth microdilution/*Bacillus subtilis*	25 µg/mL (MIC)	Chloramphenicol 3.13 µg/mL (MIC)	[55]	
		Colorimetric broth microdilution/*Pseudomonas syringae*	100 µg/mL (MIC)	Chloramphenicol 3.13 µg/mL (MIC)	[55]	
Periplanetin D (**137**)	Antivirus	Spectrophotometer/Anti-TMV	15.5% GI (20 μM)	Ningnanmycin 28.6% GI (20 μM)	[44]	
Pestalactone C (**138**)	Antifungal	Colorimetric broth microdilution/*Candida glabrata* (ATCC 90030)	3.49 μg/mL (MIC_50_)	Amphotericin B 0.25 μg/mL (MIC_50_)	[22]	
(6,8-Dihydroxy-3-methyl-1-oxo-1H-isochromen-4-yl)methyl 3-methylbutanoate (**150**)	α-Glucosidase inhibitory	Chromogenic	140.8 μM (IC_50_)	Acarbose 958.3 μM (IC_50_)	[62]	
Penicimarin F (**153**)	Antibacterial	Colorimetric broth microdilution// *S*. *aureus* (ATCC 27154)	12.5 μM (MIC)	Ciprofloxacin 0.160 μM (MIC)	[52]	
Penicisimpin A (**157**) 3-(R)-6,8-Dihydroxy-7-methyl-3-pentylisochroman-1-one	Antibacterial	Well diffusion method/*E. coli*	4 µg/mL (MIC)	Chloramphenicol 2 µg/mL (MIC)	[58]	
	Antibacterial	Well diffusion method/*Micrococcus luteus*	8 µg/mL (MIC)	Chloramphenicol 1 µg/mL (MIC)	[58]	
	Antibacterial	Well diffusion method/*Pseudomonas aeruginosa*	4 µg/mL (MIC)	Chloramphenicol 4 µg/mL (MIC)	[58]	
	Antibacterial	Well diffusion method/*Vibrio alginolyticus*	8 µg/mL (MIC)	Chloramphenicol 0.5 µg/mL (MIC)	[58]	
	Antibacterial	Well diffusion method/*Vibrio harveyi*	4 µg/mL (MIC)	Chloramphenicol 2 µg/mL (MIC)	[58]	
	Antibacterial	Well diffusion method/*Vibrio parahaemolyticus*	4 µg/mL (MIC)	Chloramphenicol 2 µg/mL (MIC)	[58]	
	Antifungal	Well diffusion method/*Colletotrichum gloeosprioides*	4 µg/mL (MIC)	Amphotericin B 8 µg/mL (MIC)	[58]	
	Antifungal	Well diffusion method/*Phytophthora parasitica* var. nicotianae	6 µg/mL (MIC)	Amphotericin B 16 µg/mL (MIC)	[58]	
Penicisimpin B (**158**) 3-(R)-6,8-Dihydroxy-3-pentylisochroman-1-one	Antibacterial	Well diffusion method/*Aeromonas hydrophilia*	32 µg/mL (MIC)	Chloramphenicol 32 µg/mL (MIC)	[58]	
	Antibacterial	Well diffusion method/*E. coli*	32 µg/mL (MIC)	Chloramphenicol 2 µg/mL (MIC)	[58]	
	Antibacterial	Well diffusion method/*Micrococcus luteus*	64 µg/mL (MIC)	Chloramphenicol 1 µg/mL (MIC)	[58]	
	Antibacterial	Well diffusion method/*Pseudomonas aeruginos*a	32 µg/mL (MIC)	Chloramphenicol 4 µg/mL (MIC)	[58]	
	Antibacterial	Well diffusion method/*Vibrio alginolyticus*	32 µg/mL (MIC)	Chloramphenicol 0.5 µg/mL (MIC)	[58]	
	Antibacterial	Well diffusion method/*Vibrio harveyi*	16 µg/mL (MIC)	Chloramphenicol 2 µg/mL (MIC)	[58]	
	Antibacterial	Well diffusion method/*Vibrio parahaemolyticus*	32 µg/mL (MIC)	Chloramphenicol 2 µg/mL (MIC)	[58]	
	Antifungal	Well diffusion method/*Colletotrichum gloeosprioides*	16 µg/mL (MIC)	Amphotericin B 8 µg/mL (MIC)	[58]	
	Antifungal	Well diffusion method/*Phytophthora parasitica* var. nicotianae	32 µg/mL (MIC)	Amphotericin B 16 µg/mL (MIC)	[58]	
Penicisimpin C (**159**) 3-(S)-6,8-Dihydroxy-7-methyl-3-(pent-1-enyl)isochroman-1-one	Antibacterial	Well diffusion method/*Aeromonas hydrophilia*	16 µg/mL (MIC)	Chloramphenicol 32 µg/mL (MIC)	[58]	
	Antibacterial	Well diffusion method/*E. coli*	8 µg/mL (MIC)	Chloramphenicol 2 µg/mL (MIC)	[58]	
	Antibacterial	Well diffusion method/*Micrococcus luteus*	16 µg/mL (MIC)	Chloramphenicol 1 µg/mL (MIC)	[58]	
	Antibacterial	Well diffusion method/*Pseudomonas aeruginosa*	8 µg/mL (MIC)	Chloramphenicol 4 µg/mL (MIC)	[58]	
	Antibacterial	Well diffusion method/*Vibrio alginolyticus*	16 µg/mL (MIC)	Chloramphenicol 0.5 µg/mL (MIC)	[58]	
	Antibacterial	Well diffusion method/*Vibrio harveyi*	8 µg/mL (MIC)	Chloramphenicol 2 µg/mL (MIC)	[58]	
	Antibacterial	Well diffusion method/*Vibrio parahaemolyticus*	8 µg/mL (MIC)	Chloramphenicol 2 µg/mL (MIC)	[58]	
	Antifungal	Well diffusion method/*Colletotrichum gloeosprioides*	8 µg/mL (MIC)	Amphotericin B 8 µg/mL (MIC)	[58]	
Fusarentin 6,7-dimethyl ether (**160**)	Antioxidant	ORAC	14.4 µM (IC_50_)	Trolox 1 µM (IC_50_)	[80]	
Fusarentin 6-methyl ether (**161**)	Antioxidant	DPPH	16.4 μM (IC_50_)	Ascorbic acid 21.2 μM (IC_50_)	[80]	
	Antioxidant	ORAC	1.4 µM (IC_50_)	Trolox 1 µM (IC_50_)	[80]	
Monocerin (**165**)	Antibacterial	Agar diffusion/*E. coli*	10 mm (IZD)	Penicillin 18 mm (IZD) Tetracycline 18 mm (GI)	[49]	
	Antibacterial	Agar diffusion/*Bacillus megaterium*	6 mm (GI)	Penicillin 14 mm (IZD) Tetracycline 18 mm (GI)	[49]	
	Antifungal	Agar diffusion/*Microbotryum violaceum*	23 mm (IZD)	Actidione 35 mm (IZD)	[49]	
	Antialgal	Agar diffusion/*Chlorella fusca*	8 mm (IZD)	Nystatin 20 mm (IZD) Actidione 50 mm (IZD)	[49]	
	Antibacterial	Microbroth dilution/*E. coli* (ATCC 25922)	15.62 μg/mL (MIC)	-	[59]	
	Antibacterial	*Pseudomonas aeruginosa* (ATCC 27853)	15.62 μg/mL (MIC)	-	[59]	
	Antibacterial	*S. aureus* (ATCC 25923)	15.62 μg/mL (MIC)	-	[59]	
	Antibacterial	*B. subtilis* (ATCC 6633)	15.62 μg/mL (MIC)	-	[59]	
	Antibacterial	*Salmonella Typhimurium* (ATCC14028)	31.25 μg/mL (MIC)	-	[59]	
	Antioxidant	ORAC	10.8 µM (IC_50_)	Trolox 1 µM (IC_50_)	[80]	
	Antimalarial	Microculture radioisotope/*P. falciparum* (K1)	0.68 μM (IC_50_)	Dihydroartemisinin 0.0004 µM (IC_50_)	[46]	
7-*O*-Demethylmonocerin (**166**)	Antioxidant	XXO	52.6 μM (IC_50_)	-	[80]	
	Antioxidant	DPPH	23.4 μM (IC_50_)	Ascorbic acid 21.2 μM (IC_50_)	[80]	
	Antioxidant	ORAC	11.5 µM (IC_50_)	Trolox 1 µM (IC_50_)	[80]	
	Antioxidant	DPPH	38 μM (EC_50_)	Ascorbic acid 39 μM (EC_50_)	[64]	
(12R)-12-Hydroxymonocerin (**167**)	Antifungal	Agar diffusion/*Microbotryum violaceum*	7 mm (IZD)	Actidione 35 mm (IZD)	[49]	
	Antialgal	Agar diffusion/*Chlorella fusca*	6 mm (IZD)	Nystatin 20 mm (IZD) Actidione 50 mm (IZD)	[49]	
	Antifungal	Colorimetric broth microdilution/*F. oxysporum*	20 µg/mL (MIC)	Amphotericin B 0.63 µg/mL (MIC)	[60]	
(11R)-Hydroxymonocerin (**168**)	Antimalarial	Microculture radioisotope/*P. falciparum* (K1)	7.7 μM (IC_50_)	Dihydroartemisinin 0.004 µM (IC_50_)	[46]	
(12S)-Hydroxymonocerin (**169**)	Antibacterial	Agar diffusion/*E. coli*	8 mm (IZD)	Penicillin 18 mm (IZD) Tetracycline 18 mm (GI)	[49]	
	Antibacterial	Agar diffusion/*B. megaterium*	6 mm (GI)	Penicillin 14 mm (IZD) Tetracycline 18 mm (GI)	[49]	
	Antifungal	Agar diffusion/*Microbotryum violaceum*	9 mm (IZD)	Actidione 35 mm (IZD)	[49]	
	Antialgal	Agar diffusion/*Chlorellafusca*	10 mm (IZD)	Nystatin 20 mm (IZD) Actidione 50 mm (IZD)	[49]	
Exserolide C (**177**)	Antifungal	Colorimetric broth microdilution/*F. oxysporum*	20 µg/mL (MIC)	Amphotericin B 0.63 µg/mL (MIC)	[60]	
Alternariol (**188**)	Antimicrobial	Colorimetric broth microdilution/*M. tetragenus*	50 µg/mL (MIC)	Streptomycin 3.125 µg/mL (MIC) Acheomycin 3.125 µg/mL (MIC) Ampicillin 3.125 µg/mL (MIC)	[57]	
Alternariol 5-*O*-methyl ether (**189**)	Antimicrobial	Colorimetric broth microdilution/*B. megaterium*	12.5 µg/mL (MIC)	Streptomycin 3.125 µg/mL (MIC) Acheomycin 3.125 µg/mL (MIC) Ampicillin 3.125 µg/mL (MIC)	[57]	
Cycloepoxylactone (**197**)	Antibacterial	Agar diffusion/*Bacillus megaterium*	5 mm (GI)	Penicillin 14 mm (GI) Tetracycline 18 mm (GI)	[42]	
	Antifungal	Agar diffusion/*Microbotryum violaceum*	10 mm (IZD)	Nystatin 20 mm (IZD) Actidione 50 mm (IZD)	[42]	
Exserolide F (**201**)	Antibacterial	Colorimetric broth microdilution/*B. subtilis* (ATCC 6633	20 µg/mL (MIC)	Ampicillin 1.25 µg/mL (MIC)	[60]	
	Antibacterial	*S. aureus* (CGMCC 1.2465)	5 µg/mL (MIC)	Ampicillin 0.16 µg/mL (MIC)	[60]	
	Antibacterial	*S. pneumoniae* (CGMCC 1.1692)	10 µg/mL (MIC)	Ampicillin 10 µg/mL (MIC)	[60]	
	Antibacterial	*E. coli* (CGMCC 1.2340)	20 µg/mL (MIC)	Gentamicin 2.5 µg/mL (MIC)	[60]	
Isocitreoisocoumarinol (**202**)	Antioxidant	Luciferase	0.98 mM (IC_50_)	tBHQ 4.29 mM (IC_50_)	[65]	
(+) Citreoisocoumarin (**203**)	Antioxidant	Luciferase	1.03 mM (IC_50_)	tBHQ 4.29 mM (IC_50_)	[65]	
(+)-6-Methylcitreoisocoumarin (**204**)	α-Glucosidase inhibitory	Chromogenic	38% Inhibition (200 μM)	Acarbose 19% Inhibition (200 μM)	[31]	
	Antioxidant	Luciferase	0.98 mM (IC_50_)	tBHQ 4.29 mM (IC_50_)	[65]	
Citreoisocoumarinol (**205**)	Antioxidant	Luciferase	0.91 mM (IC_50_)	tBHQ 4.29 mM (IC_50_)	[65]	
Mucorisocoumarin A (**207**)	Antioxidant	Luciferase	0.88 mM (IC_50_)	tBHQ 4.29 mM (IC_50_)	[65]	
Mucorisocoumarin B (**208**)	Antioxidant	Luciferase	1.03 mM (IC_50_)	tBHQ 4.29 mM (IC_50_)	[65]	
Peyroisocoumarin A (**209**)	Antioxidant	Luciferase	1.93 mM (IC_50_)	tBHQ 4.29 mM (IC_50_)	[65]	
Peyroisocoumarin B (**210**)	Antioxidant	Luciferase	2.95 mM (IC_50_)	tBHQ 4.29 mM (IC_50_)	[65]	
Peyroisocoumarin C (**211**)	Antioxidant	Luciferase	1.46 mM (IC_50_)	tBHQ 4.29 mM (IC_50_)	[65]	
Aspergillumarin A (**212**)	α-Glucosidase inhibitory	Chromogenic	38.1 μM (IC_50_)	Acarbose 958.3 μM (IC_50_)	[62]	
	Antibacterial	Colorimetric broth microdilution/*S. albus* (ATCC 8799)	12.5 μM (MIC_50_)	Ciprofloxacin 0.312 μM (MIC_50_)	[52]	
Aspergillumarin B (**213**)	α-Glucosidase inhibitory	Chromogenic	193.1 μM (IC_50_)	Acarbose 958.3 μM (IC_50_)	[62]	
	Antibacterial	Colorimetric broth microdilution/S. albus (ATCC 8799)	12.5 μM (MIC_50_)	Ciprofloxacin 0.312 μM (MIC_50_)	[52]	
Penicimarin B (**214**)	α-Glucosidase inhibitory	Chromogenic	431.4 μM (IC_50_)	Acarbose 958.3 μM (IC_50_)	[62]	
Penicimarin C (**215**)	α-Glucosidase inhibitory	Chromogenic	266.3 μM (IC_50_)	Acarbose 958.3 μM (IC_50_)	[62]	
(R)-3-(R)-4,5-Dihydroxypentyl)-8-hydroxyisochroman-1-one (**216**)	α-Glucosidase inhibitory	Chromogenic	162.5 μM (IC_50_)	Acarbose 958.3 μM (IC_50_)	[62]	
5,6-Dihydroxy-3-(4-ydroxypentyl)isochroman-1-one (**217**)	α-Glucosidase inhibitory	Chromogenic	142.1 μM (IC_50_)	Acarbose 958.3 μM (IC_50_)	[62]	
Dichlorodiaportin (**220**)	Anti-inflammatory	Colourmetric/NO	67.2 μM (MIC_50_)	Indomethacin 37.5 μM (MIC_50_)	[37]	
	Antibacterial	Colorimetric broth microdilution/*Staphylococcus aureus*	25 μg/mL (MIC_50_)	Ciprofloxacin 0.25 μg/mL (MIC_50_) Gentamicin 0.1 μg/mL (MIC_50_)	[37]	
	Antibacterial	Colorimetric broth microdilution/*B. subtilis*	25 μg/mL (MIC_50_)	Ciprofloxacin 0.25 μg/mL (MIC_50_) Gentamicin 0.1 μg/mL (MIC_50_)	[37]	
	Antibacterial	Colorimetric broth microdilution/*E. coli*	50 μg/mL (MIC_50_)	Ciprofloxacin 0.25 μg/mL (MIC_50_) Gentamicin 0.1 μg/mL (MIC_50_)	[37]	
	Antibacterial	Colorimetric broth microdilution/*Klebsiella* *pneumoniae*	50 μg/mL (MIC_50_)	Ciprofloxacin 0.25 μg/mL (MIC_50_) Gentamicin 0.1 μg/mL (MIC_50_)	[37]	
	Antibacterial	Colorimetric broth microdilution/*Acinetobacter calcoaceticus*	50 μg/mL (MIC_50_)	Ciprofloxacin 0.25 μg/mL (MIC_50_) Gentamicin 0.1 μg/mL (MIC_50_)	[37]	
	Antifungal	Broth dilution/*Colletotrichum musae*	150 μg/mL (IC_50_)	Carbendazim 6.25 μg/mL (IC_50_)	[66]	
	Antifungal	Broth dilution/*Rhizoctonia solani*	150 μg/mL (IC_50_)	Carbendazim 6.25 μg/mL (IC_50_)	[66]	
Desmethyldichlorodiaportin (**221**)	Anti-inflammatory	Colourmetric/NO	33.6 μM (MIC_50_)	Indomethacin 37.5 μM (MIC_50_)	[37]	
	Antibacterial	Colorimetric broth microdilution/*S. aureus*	25 μg/mL (MIC_50_)	Ciprofloxacin 0.25 μg/mL (MIC_50_) Gentamicin 0.1 μg/mL (MIC_50_)	[37]	
	Antibacterial	Colorimetric broth microdilution/*B. subtilis*	25 μg/mL (MIC_50_)	Ciprofloxacin 0.25 μg/mL (MIC_50_) Gentamicin 0.1 μg/mL (MIC_50_)	[37]	
	Antibacterial	Colorimetric broth microdilution/*E. coli*	25 μg/mL (MIC_50_)	Ciprofloxacin 0.25 μg/mL (MIC_50_) Gentamicin 0.1 μg/mL (MIC_50_)	[37]	
	Antibacterial	Colorimetric broth microdilution/*Klebsiella pneumoniae*	25 μg/mL (MIC_50_)	Ciprofloxacin 0.25 μg/mL (MIC_50_) Gentamicin 0.1 μg/mL (MIC_50_)	[37]	
	Antibacterial	Colorimetric broth microdilution/*Acinetobacter calcoaceticus*	50 μg/mL (MIC_50_)	Ciprofloxacin 0.25 μg/mL (MIC_50_) Gentamicin 0.1 μg/mL (MIC_50_)	[37]	
Peniisocoumarin D (**222**)	α-Glucosidase inhibitory	Chromogenic	41% Inhibition (200 μM)	Acarbose 19% Inhibition (200 μM)	[31]	
Peniisocoumarin E (**223**)	α-Glucosidase inhibitory	Chromogenic	158.4 μM (IC_50_)	Acarbose 958.3 μM (IC_50_)	[31]	
Peniisocoumarin F (**224**)	α-Glucosidase inhibitory	Chromogenic	110.3 μM (IC_50_)	Acarbose 958.3 μM (IC_50_)	[31]	
Peniisocoumarin G (**225**)	α-Glucosidase inhibitory	Chromogenic	40.5 μM (IC_50_)	Acarbose 958.3 μM (IC_50_)	[31]	
Peniisocoumarin H (**226**)	α-Glucosidase inhibitory	Chromogenic	43% Inhibition (200 μM)	Acarbose 19% Inhibition (200 μM)	[31]	
Peniisocoumarin I (**227**)	α-Glucosidase inhibitory	Chromogenic	78.1 μM (IC_50_)	Acarbose 958.3 μM (IC_50_)	[31]	
Peniisocoumarin J (**228**)	α-Glucosidase inhibitory	Chromogenic	45.1 μM (IC_50_)	Acarbose 958.3 μM (IC_50_)	[31]	
3-[(R)-3,3-Dichloro-2-hydroxypropyl]-8-hydroxy- 6-methoxy-1H-isochromen-1-one (**229**)	α-Glucosidase inhibitory	Chromogenic	102.4 μM (IC_50_)	Acarbose 958.3 μM (IC_50_)	[31]	
(+)-Diaporthin (**230**)	α-Glucosidase inhibitory	Chromogenic	33% Inhibition (200 μM)	Acarbose 19% Inhibition (200 μM)	[31]	
Diaportinol (**231**)	Antioxidant	Luciferase	0.85 mM (IC_50_)	tBHQ 4.29 mM (IC_50_)	[65]	
(+)-(10R)-7-Hydroxy-3-(2-hydroxy-propyl)-5,6-dimethylisochromen-1-one (**232**)	Antibacterial	Colorimetric broth microdilution/*B. subtilis* (ATCC 6633)	19.2 μg/mL (MIC_80_)	Penicillin 0.9 μg/mL (MIC_80_)	[63]	
	Antifungal	Colorimetric broth microdilution/*Trichophyton rubrum* (ATCC 28189)	32 μg/mL (MIC_80_)	Fluconazole 1 μg/mL (MIC_80_)	[63]	
Peyroisocoumarin D (**233**)	Antioxidant	Luciferase	2.28 mM (IC_50_)	tBHQ 4.29 mM (IC_50_)	[65]	
Orthosporin (**234**)	Antioxidant	Luciferase	1.58 mM (IC_50_)	tBHQ 4.29 mM (IC_50_)	[65]	
Versicoumarin A (**251**)	Anti-TMV	Half-leaf method	28.6% (Inhibition rate)	Ningnanmycin 31.5% (Inhibition rate)	[93]	
Peniisocoumarin C (**258**)	α-Glucosidase inhibitory	Chromogenic	95% Inhibition (200 μM)	Acarbose 19% Inhibition (200 μM)	[31]	
Asperisocoumarin A (**261**)	Antioxidant	DPPH	125 μM (EC_50_)	Ascorbic acid 35 μM (EC_50_)	[76]	
Asperisocoumarin B (**262**)	α-Glucosidase inhibitory	Chromogenic	87.8 μM (IC_50_)	Acarbose628.3 μM (IC_50_)	[76]	
Asperisocoumarin C (**263**)	Antioxidant	DPPH	138 μM (EC_50_)	Ascorbic acid 35 μM (EC_50_)	[76]	
Asperisocoumarin E (**265**)	α-Glucosidase inhibitory	Chromogenic	52.3 μM (IC_50_)	Acarbose 628.3 μM (IC_50_)	[76]	
Asperisocoumarin F (**266**)	α-Glucosidase inhibitory	Chromogenic	95.6 μM (IC_50_)	Acarbose 628.3 μM (IC_50_)	[76]	
Peniisocoumarin A (**267**)	α-Glucosidase inhibitory	Chromogenic	18% Inhibition (200 μM)	Acarbose 19% Inhibition (200 μM)	[31]	
Peniisocoumarin B (**268**)	α-Glucosidase inhibitory	Chromogenic	23% Inhibition (200 μM)	Acarbose 19% Inhibition (200 μM)	[31]	
Demethylcitreoviranol (**286**)	Antioxidant	Luciferase	1.06 mM (IC_50_)	tBHQ 4.29 mM (IC_50_)	[65]	
Citreoviranol (**287**)	Antioxidant	Luciferase	1.44 mM (IC_50_)	tBHQ 4.29 mM (IC_50_)	[65]	
Dichlorodiaportinolide (**290**)	Antifungal	Broth dilution/*Colletotrichum musae*	25 μg/mL (IC_50_)	Carbendazim 6.25 μg/mL (IC_50_)	[66]	
	Antifungal	Broth dilution/*Rhizoctonia solani*	6.25 μg/mL (IC_50_)	Carbendazim 6.25 μg/mL (IC_50_)	[66]	
Dichlorodiaportintone (**291**)	Anti-inflammatory	Colourmetric/NO	41.5 μM (MIC_50_)	Indomethacin 37.5 μM (MIC_50_)	[37]	
	Antibacterial	Colorimetric broth microdilution/*S. aureus*	50 μg/mL (MIC_50_)	Ciprofloxacin 0.25 μg/mL (MIC_50_) Gentamicin 0.1 μg/mL (MIC_50_)	[37]	
	Antibacterial	Colorimetric broth microdilution/*E. coli*	50 μg/mL (MIC_50_)	Ciprofloxacin 0.25 μg/mL (MIC_50_) Gentamicin 0.1 μg/mL (MIC_50_)	[37]	
	Antibacterial	Colorimetric broth microdilution/*Klebsiella pneumoniae*	50 μg/mL (MIC_50_)	Ciprofloxacin 0.25 μg/mL (MIC_50_) Gentamicin 0.1 μg/mL (MIC_50_)	[37]	
Desmethyldichlorodiaportintone (**292**)	Anti-inflammatory	Colourmetric/NO	15.8 μM (MIC_50_)	Indomethacin 37.5 μM (MIC_50_)	[37]	
Botryoisocoumarin A (**293**)	Anti-inflammatory	Colourmetric/COX-2	6.51 μM (IC_50_)	-	[36]	

**Table 3:** Cytotoxic activity of the most active fungal isocoumarins.

Compound Name	Assay, Cell Line	Cytotoxicity Results	Positive Control	Referencs
Penicisimpin A (**157**) 3-(*R*)-6,8-Dihydroxy-7-methyl-3-pentylisochroman-1-one	Brine shrimp (*Artemia salina*) lethality	7.7 µg/mL (LD_50_)	Colchicine 16.5 µg/mL (LD_50_)	[58]
Penicisimpin B (**158**) 3-(*R*)-6,8-Dihydroxy-3-pentylisochroman-1-one		36.4 µg/mL (LD_50_)	Colchicine 16.5 µg/mL (LD_50_)	[58]
Penicisimpin C (**159**) 3-(*S*)-6,8-Dihydroxy-7-methyl-3-(pent-1-enyl)isochroman-1-one		18.6 µg/mL (LD_50_)	Colchicine 16.5 µg/mL (LD_50_)	[58]
7-*O*-Demethylmonocerin (**166**)	MTT/HepG2	23.7 μM (IC_50_)	Etoposide 15.8 μM (IC_50_)	[80]
Aspergisocoumrin A (**274**)		43.70 μM (IC_50_)	Epirubicin 0.32 μM (IC_50_)	[73]
Dichlorodiaportinol A (**289**)		39.6 μg/mL (EC_50_)	Epirubicin 5.2 μg/mL(IC_50_)	[79]
(+) Citreoisocoumarin (**203**)	MTT/L5178Y	99.5% GI (10 μg/mL)	0.1% EGMME/DMSO	[40]
Desmethyldiaportinol (**219**)		7.3 μg/mL (EC_50_)	Kahalalide F 6.4 μg/mL (EC_50_)	[40]
Desmethyldichlorodiaportin (**221**)		41.4% GI (10 μg/mL)	0.1% EGMME/DMSO	[40]
6,6′-Dinor-bipenicilisorin (**259**)		13% GI (10 μg/mL)	Gerfelin 85% GI (10 μg/mL)	[81]
6,6′,9′-Trinor-bipenicilisorin (**260**)		33% GI (10 μg/mL)	Gerfelin 85% GI (10 μg/mL)	[81]
Versicoumarin A (**251**)	MTT/MCF7	3.8 μM (IC_50_)	Taxol 0.1 μM (IC_50_)	[93]
Versicoumarin D (**252**)		8.0 μM (IC_50_)	Taxol	[89]
Dichlorodiaportinol A (**289**)		17.8 μg/mL (EC_50_)	Epirubicin 5.3 μg/mL (IC_50_)	[79]
Peniisocoumarin G (**225**)	MTT/MptpB	20.1 μM (IC_50_)	Oleanolic acid 22.1 µM (IC_50_)	[31]
Versicoumarin A (**251**)	MTT/A549	4.0 μM (IC_50_)	Taxol 0.02 μM (IC_50_)	[93]
Versicoumarin D (**252**)	MTT/A549	5.8 μM (IC_50_)	Taxol	[89]
Aspergisocoumrin A (**274**)	MTS/MDA-MB-435	5.08 μM (IC_50_)	Epirubicin 0.26 μM (IC_50_)	[73]
Aspergisocoumrin B (**275**)		4.98 μM (IC_50_)	Epirubicin 0.26 μM (IC_50_)	[73]
Aspergisocoumrin A (**274**)	MTS/MCF10A	11.34 μM (IC_50_)	Epirubicin 0.13 μM (IC_50_)	[73]
Aspergisocoumrin B (**275**)		21.40 μM (IC_50_)	Epirubicin 0.13 μM (IC_50_)	[73]
Aspergisocoumrin A (**274**)	MTS/H460	21.53 μM (IC_50_)	Epirubicin 0.12 μM (IC_50_)	[73]
Isocoumarindole A (**296**)	CCK colorimetric/MIA-PACA-2	1.63 μM (IC_50_)	Gemcitabine 1.02 μM (IC_50_)	[29]
	CCK colorimetric/ASPC-1	5.53 μM (IC_50_)	Gemcitabine 20.10 μM (IC_50_)	[29]

## Abbreviations

1D: One Dimensional; 2D: Two Dimensional; A2780: Human Ovarian Cancer Cell Line; A375-S2: Human Malignant Melanoma Cell Line; A375-C5: Human Malignant Melanoma IL-1 Insensitive; A549: Human Lung Carcinoma Cell Line; AChE; Acetylcholinesterase; AGS; Gastric Adenocarcinoma Cell Line; ARE: Antioxidant Response Element; ARK5: NUAK Family SNF1-Like Kinase 1; ASPC-1: Pancreatic Adenocarcinoma Cell Line; Aurora A: Aurora A Kinase: Aurora B: Aurora B Kinase; BFHEnz: Bifunctional Hybrid Enzyme; BGC823: Kind of Human Gastric Carcinoma Cell Line; BHA: 3-*t*-Butyl-4-Hydroxyanisole; BHT: Butylated Hydroxytoluene; b-RAF: Proto-oncogene, Serine/Threonine Kinase; BT474: Breast Cancer Cell Line; CCK: Cholecystokinin; CD: Circular Dichroism; DPPH: 2,2-Diphenyl-1-picrylhydrazyl; CHCl_3_: Chloroform; CH_2_Cl_2_: Dichloromethane; CHAGO: Human Lung Bronchus Carcinoma Cell Line; COSY: Correlation Spectroscopy: COX-2: Cyclooxygenase-2; CPE: Cytopathic Effect; CYP19: Aromatase; DHICs: Dihydroisocoumarins; DMEM: Dulbecco’s Modified Eagle Medium; DMSO: Dimethyl Sulfoxide; DU145: Human Prostate Cancer Cell Line; EC_50_: Half Maximal Effective Concentration; EtOAc: Ethyl Acetate; FLT3: fms Related Tyrosine Kinase 3; GI: Growth Inhibition; H460: Human Lung Cancer Cell Line; HCT 116: Human Colon Cancer Cell Line; HeLa: Human Cervix Carcinoma Cell Line; HEK293T: Human embryonic kidney Cell Line; HEP-1: Hepatoma Cancer cell line; HepG2: Liver Hepatocellular Carcinoma Cell Line; HL-60: Human Caucasian Promyelocytic Leukemia Cell Line; HMBC: Heteronuclear Multiple Bond Correlation; HSQC: Heteronuclear Single Quantum Coherence; HuCCA-1: Human Lung Cholangiocarcinoma Cell Line; HUVEC: Human Umbilical Vein Endothelial Cell Line; HUAEC: Human Umbilical Artery Endothelial Cells; IC: Inhibitory Concentration; IC_50_: The Half Maximal Inhibitory Concentration; IGF-1R: Insulin like Growth Factor 1 Receptor; IL-6: Interleukin 6; IL-12 p40: Interleukin-12 Subunit Beta; IR: Infrared Radiation; IZD: Inhibition Zone Diameter; K562: Human Immortalised Myelogenous Leukemia Cell Line; KATO-3: Human Gastric Cancer; L5178Y: Mouse Lymphoma Cell Line; LPS: Lipopolysaccharide; LD: Lethal Dose; MABA; Microplate Alamar Blue Assay; MBC: Minimum Bactericidal Concentration; MCF-7: Human Breast Adenocarcinoma Cell Line; MCF10A: Immortalized Non-Cancer Breast Epithelial Cell Line; MDA-MB-435: Human Breast Cancer Cell Line; MeCN: Acetonitrile; MeOH: Methanol; MIC: Minimum Inhibitory Concentration; MIA-PACA-2: Pancreas Ductal Adenocarcinoma Cell Line; MOLT-3 and MOLT-4: Acute Lymphoblastic Leukemia Cell Line; Molm 13: Human Acute Myeloid Leukemia Cell Line; MptpB: Mycobacterium Protein Tyrosine Phosphatase B; MRC-5: Permanent Lung Fibroblast Cell Line; MRSA: Methicillin-Resistant *Staphylococcus aureus*: MS: Mass spectrometry; MTPA: α-Trifluoromethylphenylacetic Acid: MTS: One Solution Cell Proliferation; MTT: Microculture Tetrazolium Assay; NB4: Human Acute Promyelocytic Leukemia Cell line; NCIH460; Human Lung Cancer Cell Line; NMR: Nuclear Magnetic Resonance; NO: Nitric Oxide; NOE: Nuclear Overhauser Effect; NOESY; Nuclear Overhauser Effect Spectroscopy; Nrf2: Nuclear Factor E2-Related Factor 2; ORAC: Oxygen Radical Absorbance Capacity; PC-12: Adrenal Phaeochromocytoma Cell Line; PC-3: Human Prostate Cancer Cell Line; PDGF-Rbeta: Platelet-Derived Growth Factor Receptor Beta; PE: Petroleum Ether; PG: Propyl Gallate; PI: Partial Inhibition; PKS: Polyketide Synthetase; RAW 264.7: Mouse Macrophage; ROESY: Rotating Frame Nuclear Overhauser Effect Correlation Spectroscopy; RP-18: Reversed Phase C-18; SAK: Staphylokinase; SAM: S-Adenosyl Methionine; SMMC-7721: Hepatocellular Carcinoma Cell line; SGC-7901: Human Gastric Cancer Cell Line; SHSY5Y: Neuroblastoma Cell Line; SiO_2_ CC: Silica gel Column Chromatography; SW-620: Human Caucasian Colon Adenocarcinoma Cell Line; SW-480: Colorectal Adenocarcinoma Cell Line; TBHQ: Tertiary Butylhydroquinone; THP-1: Human Monocytic Cancer Cell Line; TMV: Tobacco Mosaic Virus; TNF-α: Tumor Necrosis Factor Alpha; TPA: 12-*O*-Tetradecanoylphorbol-13-Acetate; TLC: Thin Layer Chromatography; U2OS: Human Osteosarcoma Cell Line; U937: Human Macrophage (Mφ) Cancer Cell Line; UV: Ultraviolet; VEGF-R2: Vascular Endothelial Growth Factor A: VEGF-R3: Vascular Endothelial Growth Factor 3; VLC: Vacuum Liquid Chromatography; XO: Xanthine Oxidase; XXO: Xanthine/Xanthine Oxidase.

## References

[1] Ibrahim S.R., Abdallah H.M., Mohamed G.A., Ross S.A. (2016). Integracides HJ: New tetracyclic triterpenoids from the endophytic fungus *Fusarium* sp.. Fitoterapia.

[2] Ibrahim S.R., Mohamed G.A., Khedr A.I. (2017). γ-Butyrolactones from *Aspergillus* species: Structures, biosynthesis, and biological activities. Nat. Prod. Commun..

[3] Ibrahim S.R., Elkhayat E.S., Mohamed G.A., Khedr A.I., Fouad M.A., Kotb M.H., Ross S.A. (2015). Aspernolides F and G, new butyrolactones from the endophytic fungus *Aspergillus terreus*. Phytochem. Lett..

[4] Ibrahim S.R., Mohamed G.A., Moharram A.M., Youssef D.T. (2015). Aegyptolidines A and B: New pyrrolidine alkaloids from the fungus *Aspergillus aegyptiacus*. Phytochem. Lett..

[5] Ibrahim S.R., Mohamed G.A., Al Haidari R.A., El-Kholy A.A., Zayed M.F. (2018). Potential anti-malarial agents from endophytic fungi: A review. Mini Rev. Med. Chem..

[6] Ibrahim S.R., Mohamed G.A., Al Haidari R.A., El-Kholy A.A., Zayed M.F., Khayat M.T. (2018). Biologically active fungal depsidones: Chemistry, biosynthesis, structural characterization, and bioactivities. Fitoterapia.

[7] Tan R.X., Zou W.X. (2001). Endophytes: A rich source of functional metabolites. Nat. Prod. Rep..

[8] Gunatilaka A.L. (2006). Natural products from plant-associated microorganisms: Distribution, structural diversity, bioactivity, and implications of their occurrence. J. Nat. Prod..

[9] Ibrahim S.R., Mohamed G.A., Ross S.A. (2016). Integracides F and G: New tetracyclic triterpenoids from the endophytic fungus *Fusarium* sp.. Phytochem. Lett..

[10] Elkhayat E.S., Ibrahim S.R., Mohamed G.A., Ross S.A. (2016). Terrenolide S, a new antileishmanial butenolide from the endophytic fungus *Aspergillus terreus*. Nat. Prod. Res..

[11] Saeed A., Qasim M. (2014). Total synthesis of cytotoxic metabolite (±)-desmethyldiaportinol from *Ampelomyces* sp.. Nat. Prod. Res..

[12] Pal S., Chatare V., Pal M. (2011). Isocoumarin and its derivatives: An overview on their synthesis and applications. Curr. Org. Chem..

[13] Pal S., Pal M. (2018). Isocoumarin, Thiaisocoumarin and Phosphaisocoumarin: Natural Occurrences, Synthetic Approaches and Pharmaceutical Applications.

[14] Lutz-Kutschera G., Engelmeier D., Hadacek F., Werner A., Greger H., Hofer O. (2003). Synthesis of side chain substituted 3-butylisocoumarins and absolute configurations of natural isocoumarins from Artemisia dracunculus. Monatsh. Chem..

[15] Engelmeier D., Hadacek F., Hofer O., Lutz-Kutschera G., Nagl M., Wurz G., Greger H. (2004). Antifungal 3-butylisocoumarins from asteraceae-anthemideae. J. Nat. Prod..

[16] Barry R. (1964). Isocoumarins. Development since 1950. Chem. Rev..

[17] Oliveira C.M., Regasini L.O., Silva G.H., Pfenning L.H., Young M.C., Berlinck R.G., Bolzani V.S., Araujo A.R. (2011). Dihydroisocoumarins produced by *Xylaria* sp. and *Penicillium* sp., endophytic fungi associated with *Piper aduncum* and *Alibertia macrophylla*. Phytochem. Lett..

[18] Oliveira C.M., Silva G.H., Regasini L.O., Zanardi L.M., Evangelista A.H., Young M.C., Bolzani V.S., Araujo A.R. (2009). Bioactive metabolites produced by *Penicillium* sp. 1 and sp. 2, two endophytes associated with *Alibertia macrophylla* (Rubiaceae). Z. Naturforsch. C.

[19] Kuramata M., Fujioka S., Shimada A., Kawano T., Kimura Y. (2007). Citrinolactones A, B and C, and sclerotinin C, plant growth regulators from *Penicillium citrinum*. Biosci. Biotechnol. Biochem..

[20] Krohn K., Flörke U., Rao M.S., Steingröver K., Aust H.-J., Draeger S., Schulz B. (2001). Metabolites from fungi 15. New isocoumarins from an endophytic fungus isolated from the Canadian thistle *Cirsium arvense*. Nat. Prod. Lett..

[21] Wu C., Zhu H., van Wezel G.P., Choi Y.H. (2016). Metabolomics-guided analysis of isocoumarin production by *Streptomyces* species MBT76 and biotransformation of flavonoids and phenylpropanoids. Metabolomics.

[22] Song R.-Y., Wang X.-B., Yin G.-P., Liu R.-H., Kong L.-Y., Yang M.-H. (2017). Isocoumarin derivatives from the endophytic fungus, *Pestalotiopsis* sp.. Fitoterapia.

[23] Kurosaki F., Kizawa Y., Nishi A. (1989). Biosynthesis of dihydroisocoumarin by extracts of elicitor-treated carrot root. Phytochemistry.

[24] Turner W.B. (1971). Fungal Metabolites.

[25] Scott F.E., Simpson T.J., Trimble L.A., Vederas J.C. (1984). Biosynthesis of monocerin. Incorporation of 2 H-, 13 C-, and 18 O-labelled acetates by *Drechslera ravenelii*. J. Chem. Soc. Chem. Commun..

[26] Liu S.-S., Jiang J.-X., Huang R., Wang Y.-T., Jiang B.-G., Zheng K.-X., Wu S.-H. (2019). A new antiviral 14-nordrimane sesquiterpenoid from an endophytic fungus *Phoma* sp.. Phytochem. Lett..

[27] Krohn K., Sohrab M.H., Aust H.-J., Draeger S., Schulz B. (2004). Biologically active metabolites from fungi, 19: New isocoumarins and highly substituted benzoic acids from the endophytic fungus, *Scytalidium* sp.. Nat. Prod. Res..

[28] Prompanya C., Fernandes C., Cravo S., Pinto M., Dethoup T., Silva A., Kijjoa A. (2015). A new cyclic hexapeptide and a new isocoumarin derivative from the marine sponge-associated fungus *Aspergillus similanensis* KUFA 0013. Mar. Drugs.

[29] Chen M., Wang R., Zhao W., Yu L., Zhang C., Chang S., Li Y., Zhang T., Xing J., Gan M. (2019). Isocoumarindole A, a chlorinated isocoumarin and indole alkaloid hybrid metabolite from an endolichenic fungus *Aspergillus* sp.. Org. Lett..

[30] Chaudhary N.K., Pitt J.I., Lacey E., Crombie A., Vuong D., Piggott A.M., Karuso P. (2018). Banksialactones and Banksiamarins: Isochromanones and Isocoumarins from an Australian Fungus, *Aspergillus banksianus*. J. Nat. Prod..

[31] Cai R., Wu Y., Chen S., Cui H., Liu Z., Li C., She Z. (2018). Peniisocoumarins A–J: Isocoumarins from *Penicillium commune* QQF-3, an endophytic fungus of the mangrove plant *Kandelia candel*. J. Nat. Prod..

[32] Elsebai M.F., Ghabbour H.A. (2016). Isocoumarin derivatives from the marine-derived fungus *Phoma* sp. 135. Tetrahedron Lett..

[33] Cui H., Liu Y., Nie Y., Liu Z., Chen S., Zhang Z., Lu Y., He L., Huang X., She Z. (2016). Polyketides from the mangrove-derived endophytic fungus *Nectria* sp. HN001 and their α-glucosidase inhibitory activity. Mar. Drugs.

[34] Islam M.S., Ishigami K., Watanabe H. (2007). Synthesis of (−)-mellein,(+)-ramulosin, and related natural products. Tetrahedron.

[35] Kimura Y., Nakadoi M., Shimada A., Nakajima H., Hamasaki T. (1994). Biosyntheses of sescandelin and sescandelin B: New isocoumarin compounds produced by the fungus, *Sesquicilium candelabrum*. Biosci. Biotechnol. Biochem..

[36] Ju Z., Lin X., Lu X., Tu Z., Wang J., Kaliyaperumal K., Liu J., Tian Y., Xu S., Liu Y. (2015). Botryoisocoumarin A, a new COX-2 inhibitor from the mangrove *Kandelia candel* endophytic fungus *Botryosphaeria* sp. KcF6. J. Antibiot..

[37] Chen Y., Liu Z., Liu H., Pan Y., Li J., Liu L., She Z. (2018). Dichloroisocoumarins with potential anti-Inflammatory activity from the mangrove endophytic fungus *Ascomycota* sp. CYSK-4. Mar. Drugs.

[38] Liu Y., Chen S., Liu Z., Lu Y., Xia G., Liu H., He L., She Z. (2015). Bioactive metabolites from mangrove endophytic fungus *Aspergillus* sp. 16-5B. Mar. Drugs.

[39] Aly A.H., Edrada-Ebel R., Wray V., Müller W.E., Kozytska S., Hentschel U., Proksch P., Ebel R. (2008). Bioactive metabolites from the endophytic fungus *Ampelomyces* sp. isolated from the medicinal plant *Urospermum picroides*. Phytochemistry.

[40] Aly A.H., Edrada-Ebel R., Indriani I.D., Wray V., Müller W.E., Totzke F., Zirrgiebel U., Schächtele C., Kubbutat M.H., Lin W. (2008). Cytotoxic metabolites from the fungal endophyte *Alternaria* sp. and their subsequent detection in its host plant *Polygonum senegalense*. J. Nat. Prod..

[41] Arunpanichlert J., Rukachaisirikul V., Phongpaichit S., Supaphon O., Sakayaroj J. (2015). Meroterpenoid, isocoumarin, and phenol derivatives from the seagrass-derived fungus *Pestalotiopsis* sp. PSU-ES194. Tetrahedron.

[42] Hussain H., Akhtar N., Draeger S., Schulz B., Pescitelli G., Salvadori P., Antus S., Kurtán T., Krohn K. (2009). New bioactive 2, 3-epoxycyclohexenes and isocoumarins from the endophytic fungus *Phomopsis* sp. from Laurus azorica. Eur. J. Org. Chem..

[43] Hussain H., Krohn K., Draeger S., Meier K., Schulz B. (2009). Bioactive chemical constituents of a sterile endophytic fungus from *Meliotus dentatus*. Rec. Nat. Prod..

[44] Li Q.-Q., Dang L.-Z., Zhang Y.-P., Jiang J.-X., Zhang C.-M., Xiang N.-J., Yang H.-Y., Du G., Duan Y.-Q. (2015). Isocoumarins from the fermentation products of a plant entophytic fungus *Penicillium oxalicum*. J. Asian Nat. Prod. Res..

[45] Li W., Lee C., Bang S.H., Ma J.Y., Kim S., Koh Y.-S., Shim S.H. (2016). Isochromans and related constituents from the endophytic fungus *Annulohypoxylon truncatum* of *Zizania caduciflora* and their anti-inflammatory effects. J. Nat. Prod..

[46] Sappapan R., Sommit D., Ngamrojanavanich N., Pengpreecha S., Wiyakrutta S., Sriubolmas N., Pudhom K. (2008). 11-Hydroxymonocerin from the plant endophytic fungus *Exserohilum rostratum*. J. Nat. Prod..

[47] Cao J., Li X.-M., Li X., Li H.-L., Meng L.-H., Wang B.-G. (2019). New lactone and isocoumarin derivatives from the marine mangrove-derived endophytic fungus *Penicillium coffeae* MA-314. Phytochem. Lett..

[48] Prompanya C., Dethoup T., Bessa L., Pinto M., Gales L., Costa P., Silva A., Kijjoa A. (2014). New isocoumarin derivatives and meroterpenoids from the marine sponge-associated fungus *Aspergillus similanensis* sp. nov. KUFA 0013. Mar. Drugs.

[49] Zhang W., Krohn K., Draeger S., Schulz B. (2008). Bioactive isocoumarins isolated from the endophytic fungus *Microdochium bolleyi*. J. Nat. Prod..

[50] Hussain H., Jabeen F., Krohn K., Al-Harrasi A., Ahmad M., Mabood F., Shah A., Badshah A., Rehman N.U., Green I.R. (2015). Antimicrobial activity of two mellein derivatives isolated from an endophytic fungus. Med. Chem. Res..

[51] Zhao M., Yuan L.-Y., Guo D.-L., Ye Y., Da-Wa Z.-M., Wang X.-L., Ma F.-W., Chen L., Gu Y.-C., Ding L.-S. (2018). Bioactive halogenated dihydroisocoumarins produced by the endophytic fungus *Lachnum palmae* isolated from *Przewalskia tangutica*. Phytochemistry.

[52] Qi J., Shao C.-L., Li Z.-Y., Gan L.-S., Fu X.-M., Bian W.-T., Zhao H.-Y., Wang C.-Y. (2013). Isocoumarin derivatives and benzofurans from a sponge-derived *Penicillium* sp. fungus. J. Nat. Prod..

[53] Zhao J., Zhang Y., Wang L., Wang J., Zhang C. (2012). Bioactive secondary metabolites from *Nigrospora* sp. LLGLM003, an endophytic fungus of the medicinal plant *Moringa oleifera* Lam. World J. Microbiol. Biotechnol..

[54] Hu Z.-X., Xue Y.-B., Bi X.-B., Zhang J.-W., Luo Z.-W., Li X.-N., Yao G.-M., Wang J.-P., Zhang Y.-H. (2014). Five new secondary metabolites produced by a marine-associated fungus, *Daldinia eschscholzii*. Mar. Drugs.

[55] Kokubun T., Veitch N.C., Bridge P.D., Simmonds M.S. (2003). Dihydroisocoumarins and a tetralone from *Cytospora eucalypticola*. Phytochemistry.

[56] Huang G.-L., Zhou X.-M., Bai M., Liu Y.-X., Zhao Y.-L., Luo Y.-P., Niu Y.-Y., Zheng C.-J., Chen G.-Y. (2016). Dihydroisocoumarins from the mangrove-derived fungus *Penicillium citrinum*. Mar. Drugs.

[57] Wang X.-Z., Luo X.-H., Xiao J., Zhai M.-M., Yuan Y., Zhu Y., Crews P., Yuan C.-S., Wu Q.-X. (2014). Pyrone derivatives from the endophytic fungus *Alternaria tenuissima* SP-07 of Chinese herbal medicine *Salvia przewalskii*. Fitoterapia.

[58] Xu R., Li X.-M., Wang B.-G. (2016). Penicisimpins A-C, three new dihydroisocoumarins from *Penicillium simplicissimum* MA-332, a marine fungus derived from the rhizosphere of the mangrove plant *Bruguiera sexangula* var. *rhynchopetala*. Phytochem. Lett..

[59] Pinheiro E.A., Pina J.R., Feitosa A.O., Carvalho J.M., Borges F.C., Marinho P.S., Marinho A.M. (2017). Bioprospecting of antimicrobial activity of extracts of endophytic fungi from *Bauhinia guianensis*. Rev. Argent. Microbiol..

[60] Li R., Chen S., Niu S., Guo L., Yin J., Che Y. (2014). Exserolides A–F, new isocoumarin derivatives from the plant endophytic fungus *Exserohilum* sp.. Fitoterapia.

[61] Li S., Wei M., Chen G., Lin Y. (2012). Two new dihydroisocoumarins from the endophytic fungus *Aspergillus* sp. collected from the South China Sea. Chem. Nat. Compd..

[62] Chen S., Liu Y., Liu Z., Cai R., Lu Y., Huang X., She Z. (2016). Isocoumarins and benzofurans from the mangrove endophytic fungus *Talaromyces amestolkiae* possess α-glucosidase inhibitory and antibacterial activities. RSC Adv..

[63] Wang Y., Yang M.-H., Wang X.-B., Li T.-X., Kong L.-Y. (2014). Bioactive metabolites from the endophytic fungus *Alternaria alternata*. Fitoterapia.

[64] Pang X., Lin X., Yang J., Zhou X., Yang B., Wang J., Liu Y. (2018). Spiro-Phthalides and Isocoumarins isolated from the marine-sponge-derived fungus *Setosphaeria* sp. SCSIO41009. J. Nat. Prod..

[65] Zhao Y., Liu D., Proksch P., Yu S., Lin W. (2016). Isocoumarin derivatives from the sponge-associated fungus *Peyronellaea glomerata* with antioxidant activities. Chem. Biodivers..

[66] Li W., Xu J., Li F., Xu L., Li C. (2016). A new antifungal isocoumarin from the endophytic fungus *Trichoderma* sp. 09 of *Myoporum bontioides* A. gray. Pharmacogn. Mag..

[67] Bai M., Zheng C.-J., Huang G.-L., Mei R.-Q., Wang B., Luo Y.-P., Zheng C., Niu Z.-G., Chen G.-Y. (2019). Bioactive Meroterpenoids and Isocoumarins from the Mangrove-Derived Fungus *Penicillium* sp. TGM112. J. Nat. Prod..

[68] Choudhary M.I., Musharraf S.G., Mukhmoor T., Shaheen F., Ali S., Rahman A.-U. (2004). Isolation of bioactive compounds from *Aspergillus terreus*. Z. Naturforsch. B.

[69] Ding Z., Tao T., Wang L., Zhao Y., Huang H., Zhang D., Liu M., Wang Z., Han J. (2019). Bioprospecting of novel and bioactive metabolites from endophytic fungi Isolated from rubber tree *Ficus elastica* leaves. J. Microbiol. Biotechnol..

[70] Ma X., Liang X., Huang Z.-H., Qi S.-H. (2019). New alkaloids and isocoumarins from the marine gorgonian-derived fungus *Aspergillus* sp. SCSIO 41501. Nat. Prod. Res..

[71] Wu Z., Chen J., Zhang X., Chen Z., Li T., She Z., Ding W., Li C. (2019). Four New isocoumarins and a new natural tryptamine with antifungal activities from a mangrove endophytic fungus *Botryosphaeria ramosa* L29. Mar. Drugs.

[72] Xu Z., Wu X., Li G., Feng Z., Xu J. (2018). Pestalotiopisorin B, a new isocoumarin derivative from the mangrove endophytic fungus *Pestalotiopsis* sp. HHL101. Nat. Prod. Res..

[73] Wu Y., Chen S., Liu H., Huang X., Liu Y., Tao Y., She Z. (2019). Cytotoxic isocoumarin derivatives from the mangrove endophytic fungus *Aspergillus* sp. HN15-5D. Arch. Pharm. Res..

[74] Kongsaeree P., Prabpai S., Sriubolmas N., Vongvein C., Wiyakrutta S. (2003). Antimalarial dihydroisocoumarins produced by *Geotrichum* sp., an endophytic fungus of *Crassocephalum crepidioides*. J. Nat. Prod..

[75] Lei H., Lin X., Han L., Ma J., Ma Q., Zhong J., Liu Y., Sun T., Wang J., Huang X. (2017). New metabolites and bioactive chlorinated benzophenone derivatives produced by a marine-derived fungus *Pestalotiopsis heterocornis*. Mar. Drugs.

[76] Chen S., Cai R., Hong K., She Z. (2016). New furoisocoumarins and isocoumarins from the mangrove endophytic fungus *Aspergillus* sp. 085242. Beilstein J. Org. Chem..

[77] Yan W., Cao L.-L., Zhang Y.-Y., Zhao R., Zhao S.-S., Khan B., Ye Y.-H. (2018). New metabolites from endophytic fungus *Chaetomium globosum* CDW7. Molecules.

[78] Zhang T.-Y., Wu Y.-Y., Zhang M.-Y., Cheng J., Dube B., Yu H.-J., Zhang Y.-X. (2019). New antimicrobial compounds produced by *Seltsamia galinsogisoli* sp. nov., isolated from *Galinsoga parviflora* as potential inhibitors of FtsZ. Sci. Rep..

[79] Li C., Gong B., Cox D.G., Li C., Wang J., Ding W. (2014). Dichlorodiaportinol A–A new chlorine-containing isocoumarin from an endophytic fungus *Trichoderma* sp. 09 from *Myoporum bontioides* A. Gray and its cytotoxic activity. Pharmacogn. Mag..

[80] Tianpanich K., Prachya S., Wiyakrutta S., Mahidol C., Ruchirawat S., Kittakoop P. (2010). Radical scavenging and antioxidant activities of isocoumarins and a phthalide from the endophytic fungus *Colletotrichum* sp.. J. Nat. Prod..

[81] Ebada S.S., El-Neketi M., Ebrahim W., Mándi A., Kurtán T., Kalscheuer R., Müller W.E., Proksch P. (2018). Cytotoxic secondary metabolites from the endophytic fungus *Aspergillus versicolor* KU258497. Phytochem. Lett..

[82] Kamdem R.S., Wang H., Wafo P., Ebrahim W., Özkaya F.C., Makhloufi G., Janiak C., Sureechatchaiyan P., Kassack M.U., Lin W. (2018). Induction of new metabolites from the endophytic fungus *Bionectria* sp. through bacterial co-culture. Fitoterapia.

[83] Kumar M., Qadri M., Sharma P.R., Kumar A., Andotra S.S., Kaur T., Kapoor K., Gupta V.K., Kant R., Hamid A. (2013). Tubulin inhibitors from an endophytic fungus isolated from *Cedrus deodara*. J. Nat. Prod..

[84] Sang X.-N., Chen S.-F., An X., Chen G., Wang H.-F., Pei Y.-H. (2017). A novel 3, 4-dihydronaphthalen-1 (2 H)-one with spiro-butyrolactone and a new isocoumarin isolated from the endophytic fungus *Phoma* sp. YN02-P-3. J. Asian Nat. Prod. Res..

[85] Huang Y.-F., Li L.-H., Tian L., Qiao L., Hua H.-M., Pei Y.-H. (2006). Sg17-1-4, a novel isocoumarin from a marine fungus *Alternaria tenuis* Sg17-1. J. Antibiot..

[86] Yang S.-X., Gao J.-M., Zhang Q., Laatsch H. (2011). Toxic polyketides produced by *Fusarium* sp., an endophytic fungus isolated from *Melia azedarach*. Biorg. Med. Chem. Lett..

[87] Han Z., Mei W., Zhao Y., Deng Y., Dai H. (2009). A new cytotoxic isocoumarin from endophytic fungus *Penicillium* sp. 091402 of the mangrove plant *Bruguiera sexangula*. Chem. Nat. Compd..

[88] Huang Z., Shao C., Chen Y., She Z., Lin Y., Zhou S. (2007). A new isocoumarin from mangrove endophytic fungus (No. dz17) on the South China Sea coast. Chem. Nat. Compd..

[89] Ji B.-K., Dong W., Wang Y.-D., Zhou K., Li Y.-K., Zhou M., Du G., Hu Q.-F., Ye Y.-Q., Yang H.-Y. (2015). A new isocoumarin from fermentation products of endophytic fungus of Aspergillus versicolor. Asian J. Chem..

[90] Tsukada M., Fukai M., Miki K., Shiraishi T., Suzuki T., Nishio K., Sugita T., Ishino M., Kinoshita K., Takahashi K. (2011). Chemical constituents of a marine fungus, *Arthrinium sacchari*. J. Nat. Prod..

[91] Schmeda-Hirschmann G., Hormazabal E., Astudillo L., Rodriguez J., Theoduloz C. (2005). Secondary metabolites from endophytic fungi isolated from the Chilean gymnosperm *Prumnopitys andina* (Lleuque). World J. Microbiol. Biotechnol..

[92] Pinheiro Â., Dethoup T., Bessa J., Silva A.M., Kijjoa A. (2012). A new bicyclic sesquiterpene from the marine sponge associated fungus *Emericellopsis minima*. Phytochem. Lett..

[93] Ye Y.-Q., Xia C.-F., Yang J.-X., Qin Y., Zhou M., Gao X.-M., Du G., Yang H.-Y., Li X.-M., Hu Q.-F. (2014). Isocoumarins from the fermentation products of an endophytic fungus of *Aspergillus versicolor*. Phytochem. Lett..

[94] Zhou M., Zhou K., He P., Wang K.-M., Zhu R.-Z., Wang Y.-D., Dong W., Li G.-P., Yang H.-Y., Ye Y.-Q. (2016). Antiviral and cytotoxic isocoumarin derivatives from an endophytic fungus *Aspergillus oryzae*. Planta Med..

[95] Rukachaisirikul V., Buadam S., Sukpondma Y., Phongpaichit S., Sakayaroj J., Hutadilok-Towatana N. (2013). Indanone and mellein derivatives from the *Garcinia*-derived fungus *Xylaria* sp. PSU-G12. Phytochem. Lett..

[96] Sritharan T., Savitri Kumar N., Jayasinghe L., Araya H., Fujimoto Y. (2019). Isocoumarins and dihydroisocoumarins from the endophytic fungus *Biscogniauxia capnodes* isolated from the fruits of *Averrhoa carambola*. Nat. Prod. Commun..

[97] Kimura A., Lee J.-H., Lee I.-S., Lee H.-S., Park K.-H., Chiba S., Kim D. (2004). Two potent competitive inhibitors discriminating α-glucosidase family I from family II. Carbohydr. Res..

[98] Ibrahim S.R., Mohamed G.A., Abdel-Latif M.M., El-Messery S.M., Al Musayeib N.M., Shehata I.A. (2015). Minutaside A, new α-amylase inhibitor flavonol glucoside from *Tagetes minuta*: Antidiabetic, antioxidant, and molecular modeling studies. Starch-Stärke.

[99] Ibrahim S.R.M., Mohamed G.A., Khayat M.T.A., Ahmed S., Abo-Haded H. (2019). α-Amylase inhibition of xanthones from *Garcinia mangostana* pericarps and their possible use for the treatment of diabetes with molecular docking studies. J. Food Biochem..

[100] Ibrahim S.R.M., Mohamed G.A., Khayat M.T.A., Ahmed S., Abo-Haded H. (2019). Garcixanthone D, a new xanthone, and other xanthone derivatives from Garcinia mangostana pericarps: Their α-amylase inhibitory potential and molecular docking studies. Starch-Stärke.

[101] Lebovitz H.E. (1997). Alpha-glucosidase inhibitors. Endocrinol. Metab. Clin. N. Am..

[102] Indrianingsih A.W., Tachibana S. (2017). α-Glucosidase inhibitor produced by an endophytic fungus, *Xylariaceae* sp. QGS 01 from *Quercus gilva* Blume. Food Sci. Hum. Wellness.

[103] Xu Y., Wang C., Liu H., Zhu G., Fu P., Wang L., Zhu W. (2018). Meroterpenoids and Isocoumarinoids from a *Myrothecium* Fungus Associated with *Apocynum venetum*. Mar. Drugs.

[104] Liao M.-F., Wang K., Ren J.-W., Liu L., Cai L., Han J.-J., Liu H.-W. (2019). 2 H-Pyranone and isocoumarin derivatives isolated from the plant pathogenic fungus *Leptosphaena maculans*. J. Asian Nat. Prod. Res..

[105] Murray A.P., Faraoni M.B., Castro M.J., Alza N.P., Cavallaro V. (2013). Natural AChE inhibitors from plants and their contribution to Alzheimer’s disease therapy. Curr. Neuropharmacol..

[106] Patel S.S., Raghuwanshi R., Masood M., Acharya A., Jain S.K. (2018). Medicinal plants with acetylcholinesterase inhibitory activity. Rev. Neurosci..

[107] Manning G., Whyte D.B., Martinez R., Hunter T., Sudarsanam S. (2002). The protein kinase complement of the human genome. Science.

[108] Liu M., Zhao G., Cao S., Zhang Y., Li X., Lin X. (2017). Development of certain protein kinase inhibitors with the components from traditional Chinese medicine. Front. Pharmacol..

[109] Fan N.W., Chang H.S., Cheng M.J., Hsieh S.Y., Liu T.W., Yuan G.F., Chen I.S. (2014). Secondary metabolites from the endophytic fungus *Xylaria cubensis*. Helv. Chim. Acta.

[110] Sommart U., Rukachaisirikul V., Sukpondma Y., Phongpaichit S., Sakayaroj J., Kirtikara K. (2008). Hydronaphthalenones and a dihydroramulosin from the endophytic fungus PSU-N24. Chem. Pharm. Bull..

[111] Tansuwan S., Pornpakakul S., Roengsumran S., Petsom A., Muangsin N., Sihanonta P., Chaichit N. (2007). Antimalarial benzoquinones from an endophytic fungus, *Xylaria* sp.. J. Nat. Prod..

[112] Duan Y.-Q., Dang L.-Z., Jiang J.-X., Zhang Y.-P., Xiang N.-J., Yang H.-M., Du G., Yang H.-Y., Li Q.-Q. (2018). Anti-tobacco Mosaic virus isocoumarins from the fermentation products of the endophytic fungus Aspergillus versicolor. Chem. Nat. Compd..

[113] Ramos H.P., Simão M.R., de Souza J.M., Magalhães L.G., Rodrigues V., Ambrósio S.R., Said S. (2013). Evaluation of dihydroisocoumarins produced by the endophytic fungus Arthrinium state of *Apiospora montagnei* against *Schistosoma mansoni*. Nat. Prod. Res..

[114] Evidente A., Punzo B., Andolfi A., Cimmino A., Melck D., Luque J. (2010). Lipophilic phytotoxins produced by *Neofusicoccum parvum*, a grapevine canker agent. Phytopathol. Mediterr..

[115] Nakashima K.-i., Tomida J., Hirai T., Morita Y., Kawamura Y., Inoue M. (2017). A new isocoumarin derivative from an endophytic fungus Thielavia sp. isolated from Crassula ovata. Heterocycles Int. J. Rev. Commun. Heterocycl. Chem..

[116] Jiao Y., Zhang X., Wang L., Li G., Zhou J.-C., Lou H.-X. (2013). Metabolites from *Penicillium* sp., an endophytic fungus from the liverwort *Riccardia multifida* (L.) S. Gray. Phytochem. Lett..

[117] Krohn K., Kock I., Elsässer B., Flörke U., Schulz B., Draeger S., Pescitelli G., Antus S., Kurtán T. (2007). Bioactive natural products from the endophytic fungus *Ascochyta* sp. from *Meliotus dentatus*–configurational assignment by solid-State CD and TDDFT calculations. Eur. J. Org. Chem..

[118] Wang F., Han S., Hu S., Xue Y., Wang J., Xu H., Chen L., Zhang G., Zhang Y. (2014). Two new secondary metabolites from *Xylaria* sp. cfcc 87468. Molecules.

[119] Sumarah M.W., Puniani E., Blackwell B.A., Miller J.D. (2008). Characterization of polyketide metabolites from foliar endophytes of *Picea glauca*. J. Nat. Prod..

[120] Qian C.-D., Fu Y.-H., Jiang F.-S., Xu Z.-H., Cheng D.-Q., Ding B., Gao C.-X., Ding Z.-S. (2014). Lasiodiplodia sp. ME4-2, an endophytic fungus from the floral parts of *Viscum coloratum*, produces indole-3-carboxylic acid and other aromatic metabolites. BMC Microbial..

[121] Tian J.-F., Yu R.-J., Li X.-X., Gao H., Hu D., Guo L.-D., Tang J.-S., Yao X.-S. (2015). Cyclohexenones and isocoumarins from an endophytic fungus of *Sarcosomataceae* sp.. J. Asian Nat. Prod. Res..

[122] Hu Q.-F., Xing H.-H., Wang Y.-D., Yu Z.-H., Yan K.-L., Zhou K., Dong W., Zhou M., Yang H.-Y., Zhu D.-L. (2017). Prenylated isocoumarins from the fermentation products of the endophytic fungus *Aspergillus versicolor* and their anti-tobacco mosaic virus activities. Chem. Nat. Compd..

[123] Luo J., Liu X., Li E., Guo L., Che Y. (2013). Arundinols A–C and arundinones A and B from the plant endophytic fungus *Microsphaeropsis arundinis*. J. Nat. Prod..

[124] Arunpanichlert J., Rukachaisirikul V., Sukpondma Y., Phongpaichit S., Tewtrakul S., Rungjindamai N., Sakayaroj J. (2010). Azaphilone and isocoumarin derivatives from the endophytic fungus *Penicillium sclerotiorum* PSU-A13. Chem. Pharm. Bull..

[125] Rukachaisirikul V., Rodglin A., Sukpondma Y., Phongpaichit S., Buatong J., Sakayaroj J. (2012). Phthalide and isocoumarin derivatives produced by an *Acremonium* sp. isolated from a mangrove *Rhizophora apiculata*. J. Nat. Prod..

[126] Zhou M., Lou J., Li Y.-K., Wang Y.-D., Zhou K., Ji B.-K., Dong W., Gao X.-M., Du G., Hu Q.-F. (2017). Versicolols A and B, two new prenylated isocoumarins from endophytic fungus *Aspergillus versicolor* and their cytotoxic activity. Arch. Pharm. Res..

[127] Huang H., Li Q., Feng X., Chen B., Wang J., Liu L., She Z., Lin Y. (2010). Structural elucidation and NMR assignments of four aromatic lactones from a mangrove endophytic fungus (No. GX4-1B). Magn. Reson. Chem..

[128] Ariefta N.R., Kristiana P., Aboshi T., Murayama T., Tawaraya K., Koseki T., Kurisawa N., Kimura K.-i., Shiono Y. (2018). New isocoumarins, naphthoquinones, and a cleistanthane-type diterpene from Nectria pseudotrichia 120-1NP. Fitoterapia.

[129] Wang Z., Fan P., Xue T.-D., Meng L.-L., Gao W.-B., Zhang J., Zhao Y.-x., Luo D.-Q. (2018). Two new isocoumarin derivatives from an endophytic fungi *Pestalotiopsis coffeae* isolated from a mangrove *Fishtail Palm*. Nat. Prod. Commun..

[130] Fang Z.F., Yu S.S., Zhou W.Q., Chen X.G., Ma S.G., Li Y., Qu J. (2012). A new isocoumarin from metabolites of the endophytic fungus *Alternaria tenuissima* (Nees & T. Nees: Fr.) Wiltshire. Chin. Chem. Lett..

[131] Shi T., Qi J., Shao C.-L., Zhao D.-L., Hou X.-M., Wang C.-Y. (2017). Bioactive diphenyl ethers and isocoumarin derivatives from a gorgonian-derived fungus *Phoma* sp.(TA07-1). Mar. Drugs.

[132] Hsiao Y., Cheng M.-J., Chang H.-S., Wu M.-D., Hsieh S.-Y., Liu T.-W., Lin C.-H., Yuan G.-F., Chen I.-S. (2016). Six new metabolites produced by *Colletotrichum aotearoa* 09F0161, an endophytic fungus isolated from *Bredia oldhamii*. Nat. Prod. Res..

[133] Wijeratne E.K., Paranagama P.A., Gunatilaka A.L. (2006). Five new isocoumarins from Sonoran desert plant-associated fungal strains *Paraphaeosphaeria quadriseptata* and *Chaetomium chiversii*. Tetrahedron.

