# Correction: Noor et al. Naturally Occurring Isocoumarins Derivatives from Endophytic Fungi: Sources, Isolation, Structural Characterization, Biosynthesis, and Biological Activities. *Molecules* 2020, *25*, 395

**DOI:** 10.3390/molecules31132239

**Published:** 2026-06-25

**Authors:** Ahmad Omar Noor, Diena Mohammedallam Almasri, Alaa Abdullah Bagalagel, Hossam Mohamed Abdallah, Shaimaa Gamal Abdallah Mohamed, Gamal Abdallah Mohamed, Sabrin Ragab Mohamed Ibrahim

**Affiliations:** 1 Pharmacy Practice Department, Faculty of Pharmacy, King Abdulaziz University, Jeddah 21589, Saudi Arabia; aonoor@kau.edu.sa (A.O.N.); dalmasri@kau.edu.sa (D.M.A.); abagalagel@kau.edu.sa (A.A.B.); 2 Department of Natural Products and Alternative Medicine, Faculty of Pharmacy, King Abdulaziz University, Jeddah 21589, Saudi Arabia; hmafifi2013@gmail.com (H.M.A.); gamals2001@yahoo.com (G.A.M.); 3 Department of Pharmacognosy, Faculty of Pharmacy, Cairo University, Cairo 11562, Egypt; 4 Faculty of Dentistry, British University, El Sherouk City, Suez Desert Road, Cairo 11837, Egypt; shaimaag1973@gmail.com; 5 Pharmacognosy Department, Faculty of Pharmacy, Al-Azhar University, Assiut Branch, Assiut 71524, Egypt; 6 Department of Pharmacognosy and Pharmaceutical Chemistry, College of Pharmacy, Taibah University, Al Madinah Al-Munawwarah 30078, Saudi Arabia; 7 Department of Pharmacognosy, Faculty of Pharmacy, Assiut University, Assiut 71526, Egypt

In the original publication [1], there was an error in Section 5.4, “α-Glucosidase, Acetylcholinesterase (AChE), and Protein Kinase Inhibitory Activities”. In the narrative description of α-glucosidase inhibitory activity, compound **224** was inadvertently included in the stronger/powerful activity group. The numerical data in Table 2 are correct and remain unchanged; the error is limited to the narrative grouping in the text.

A correction has been made to Section 5.4, “α-Glucosidase, Acetylcholinesterase (AChE), and Protein Kinase Inhibitory Activities”:

The α-glucosidase inhibitory capacities of **204**, **222**–**230**, **258**, **267**, and **268** which were biosynthesized by *Penicillium commune* were evaluated. Compounds **225**, **227**, **228**, and **258** possessed powerful activity (IC_50_ 38.1–78.1 μM) than acarbose (IC_50_ 478.4 μM). However, compounds **223**, **224**, and **229** were moderately active (IC_50_s 102.4–158.4 μM) [31].

The authors state that the scientific conclusions are unaffected. This correction was approved by the Academic Editor. The original publication has also been updated.
